# Aggregation-prone TDP-43 sequesters and drives pathological transitions of free nuclear TDP-43

**DOI:** 10.1007/s00018-023-04739-2

**Published:** 2023-03-17

**Authors:** Sean S. Keating, Adekunle T. Bademosi, Rebecca San Gil, Adam K. Walker

**Affiliations:** Neurodegeneration Pathobiology Laboratory, Clem Jones Centre for Ageing Dementia Research, Queensland Brain Institute, The University of Queensland, St. Lucia, QLD 4072 Australia

**Keywords:** Acetylated TDP-43, Endogenous gene tagging, LLPS, TDP-43 loss of function, Motor neuron disease

## Abstract

Aggregation of the RNA-binding protein, TDP-43, is the unifying hallmark of amyotrophic lateral sclerosis and frontotemporal dementia. TDP-43-related neurodegeneration involves multiple changes to normal physiological TDP-43, which undergoes nuclear depletion, cytoplasmic mislocalisation, post-translational modification, and aberrant liquid–liquid phase separation, preceding inclusion formation. Along with toxic cytoplasmic aggregation, concurrent depletion and dysfunction of normal nuclear TDP-43 in cells with TDP-43 pathology is likely a key potentiator of neurodegeneration, but is not well understood. To define processes driving TDP-43 dysfunction, we used CRISPR/Cas9-mediated fluorescent tagging to investigate how disease-associated stressors and pathological TDP-43 alter abundance, localisation, self-assembly, aggregation, solubility, and mobility dynamics of normal nuclear TDP-43 over time in live cells. Oxidative stress stimulated liquid–liquid phase separation of endogenous TDP-43 into droplet-like puncta, or spherical shell-like anisosomes. Further, nuclear RNA-binding-ablated or acetylation-mimicking TDP-43 readily sequestered and depleted free normal nuclear TDP-43 into dynamic anisosomes, in which recruited endogenous TDP-43 proteins remained soluble and highly mobile. Large, phosphorylated inclusions formed by nuclear or cytoplasmic aggregation-prone TDP-43 mutants also caused sequestration, but rendered endogenous TDP-43 immobile and insoluble, indicating pathological transition. These findings suggest that RNA-binding deficiency and post-translational modifications including acetylation exacerbate TDP-43 aggregation and dysfunction by driving sequestration, mislocalisation, and depletion of normal nuclear TDP-43 in neurodegenerative diseases.

**Supplementary Information:**

The online version contains supplementary material available at 10.1007/s00018-023-04739-2.

## Introduction

Pathological aggregation of TAR DNA-binding protein 43 (TDP-43) in neurons is a key hallmark of almost all amyotrophic lateral sclerosis (ALS) and approximately half of frontotemporal dementia (FTD) cases [1, 2]. TDP-43 is an essential DNA-/RNA-binding protein that predominantly resides in the nucleus and physiologically regulates stability, transport, splicing, and translation of many thousands of mRNA transcripts [3–6]. In disease, TDP-43 undergoes progressive changes including nuclear depletion, cytoplasmic mislocalisation, misfolding, post-translational modification, aberrant liquid–liquid phase separation, and assembly into distinct pathological species before forming inclusions [7]. Cytoplasmic accumulation of TDP-43 has been regarded as a primary driver of neuronal toxicity and dysfunction [8, 9], however, loss of normal nuclear TDP-43 has also been reported even in the absence of cytoplasmic TDP-43 inclusions in post-mortem FTD [10, 11] and ALS brains [12]. Indeed, TDP-43 nuclear depletion can independently mediate neurodegeneration [13], via dysregulation of TDP-43-dependent transcription [14] and mRNA splicing functions [3, 15, 16]. As nuclear loss and cytoplasmic accumulation of TDP-43 occur concurrently in many disease models [13, 17], the driving mechanisms and relative contributions of these processes to neuronal dysfunction or toxicity remain elusive.

Post-translational modifications (PTMs) are a major feature of TDP-43 pathology in ALS and FTD, and likely play diverse roles in TDP-43 dysfunction, aggregation propensity, and neurotoxicity. For example, phosphorylation is a widely recognised hallmark of pathology progression [18–20]. Less is understood regarding the pathogenic role of aberrant acetylation of TDP-43, which marks cytoplasmic inclusions in ALS spinal cord and is thought to affect RNA binding functions [21–23]. TDP-43 acetylation sites are found within the RNA-recognition motifs (RRMs), namely lysine residues 145 and 192 [21, 22, 24]. Mechanisms and consequences for TDP-43 acetylation have been investigated with acetylation-mimicking TDP-43 ‘2KQ’ variants, in which lysine-to-glutamine amino acid substitution at these RNA-binding residues replicates changes in electrostatic charge thought to be mediated by acetylation [21, 24, 25]. Indeed, acetylation of TDP-43 RRMs leads to substantially decreased RNA affinity and loss of splicing activity [21, 22]. Acetylation-mimicking TDP-43-2KQ mutants recapitulate native interactions of acetylated wild-type TDP-43 proteins, and further promote putative acetylation while forming disease-reminiscent insoluble and phosphorylated cytoplasmic inclusions [21, 22].

RNA deficiency promotes TDP-43 instability [26, 27], and aberrant liquid–liquid phase separation (LLPS), forming assemblies which may seed pathological aggregation [25, 28–34]. TDP-43 LLPS occurs physiologically, but can be enhanced in response to cellular stress or RNA dyshomeostasis, giving rise to diverse biomolecular condensates, characterised by ‘liquid droplet-like’ properties of subcellular mobility, fusion, fission, and dynamic exchange of comprising proteins with surrounding intracellular TDP-43 [25, 28, 30, 32, 33, 35–38]. The effects of RNA binding on TDP-43 assembly and aggregation, independent of other pathological modifications, have been studied using TDP-43 ‘FL’ mutants in which 4 or 5 essential RNA-binding phenylalanine residues in the RRMs are mutated to leucine, preventing RNA interactions [21, 25, 31, 39, 40]. RNA-binding-ablated FL mutants demonstrate increased aggregation propensity and toxicity [21, 25, 32, 39], reminiscent of truncated TDP-43 species lacking RRMs, or TDP-43 containing ALS-associated RRM mutations [29, 41–43]. Importantly, mutant RNA-binding-ablated TDP-43-5FL and acetylation-mimicking TDP-43-2KQ proteins over-expressed in the nucleus have recently been shown to undergo a distinct type of LLPS in association with molecular chaperones, readily assembling into dynamic spherical shell-like structures, termed ‘anisosomes’ [25]. TDP-43 anisosomes exclude RNA, and exhibit a ‘liquid-like’ core void of TDP-43 occupied by phase-separated heat-shock protein 70 (Hsp70), which maintains the structure and solubility of these assemblies [25]. While direct links to ALS and FTD pathology remain to be established, it is thought that a loss of chaperone ATPase activity through ageing or other stressors may drive disassembly or solidification of TDP-43 anisosomes, seeding aggregation [25]. Anisosome formation with wild-type TDP-43 may also be induced by proteolytic stress and histone deacetylase inhibition [25]. However, it remains unclear whether other disease-associated stressors trigger assembly of anisosomes comprising endogenous normal TDP-43 in human ALS and FTD.

Among additional disease-associated cellular stressors, oxidative stress is observed in brain and spinal cord of ALS and FTD patients [34, 44–47], and induces redistribution or loss of nuclear TDP-43 as well as inclusion formation in various disease models [48–50, 52–55]. The relationship between oxidative stress, pathological aggregation and nuclear depletion of TDP-43 is also linked to LLPS [31, 34, 37]. For example, under oxidative stress, cells form stress granules (SGs) that limit non-essential protein translation, involving LLPS-mediated recruitment of TDP-43 and other RNA-binding proteins [44, 45, 48, 56–58]. TDP-43 may also undergo SG-independent LLPS under oxidative stress to form nuclear or cytoplasmic liquid droplet-like ‘puncta’ that do not associate with RNA substrates or SG markers [32, 33, 37]. Chronic assembly of both TDP-43-positive SGs [34, 48, 59], and RNA-/SG-independent TDP-43 structures [24, 25, 32, 33, 59] results in conversion of reversible dynamic liquid-like structures into gel-like or solid immobile assemblies. Aberrant LLPS or disruption of protein dynamics may therefore lead to irreversible pathological transitions, denoted by loss of protein mobility. Interestingly, TDP-43 acetylation may be promoted by oxidative stress, and acetylation-mimicking TDP-43 forms cytoplasmic inclusions lacking SG markers [21, 24], which may be related to aberrant SG-independent LLPS.

Depletion of normal nuclear TDP-43 in disease could be driven by accumulation of TDP-43 protein through changes in endogenous TDP-43 expression, or sequestration into pathological inclusions or other phase-separated TDP-43 assemblies. Auto-regulation of *TARDBP* expression is mediated by TDP-43 binding its own mRNA [5, 30, 38], as seen in disease models with decreased endogenous TDP-43 protein upon over-expression of wild-type or cytoplasmic TDP-43 [13, 17, 60–62]. Additionally, exogenous wild-type TDP-43 is incorporated into cytoplasmic TDP-43 structures in cells [33, 62–64], resembling pathological inclusions observed in ALS and FTLD-TDP that contain full-length natively-folded TDP-43 [21, 26, 27, 29, 65]. However, it is unclear whether normal endogenous TDP-43 is sequestered into pathological TDP-43 structures, and whether such sequestration could deplete overall nuclear levels and impair the dynamic properties of endogenous TDP-43.

In this study, we therefore sought to determine how disease-related stressors, and RNA-binding ablation or acetylation of pathological TDP-43, affect endogenous normal TDP-43 levels, localisation, self-assembly, and mobility dynamics in the nucleus and cytoplasm of cells. We found that oxidative stress caused CRISPR-tagged endogenous TDP-43 proteins to assemble into various biomolecular condensates, including SGs, dynamic liquid-like nuclear droplets, or anisosomes, over time. Notably, over-expression and aggregation of RNA-binding-ablated, or acetylation-mimicking TDP-43 drove nuclear depletion of normal endogenous TDP-43, via concentration-dependent sequestration into inclusions, puncta, or anisosomes. Sequestration into large, phosphorylated nuclear or cytoplasmic inclusions formed by acetylated TDP-43 led to insolubility and immobility of normal TDP-43, indicating pathological transition. Alternatively, recruitment to dynamic acetylated TDP-43 anisosomes caused the most dramatic depletion of free normal nuclear TDP-43 but did not impair its mobility. Our findings therefore suggest that both acetylation and RNA-binding ablation increase TDP-43 aggregation propensity and cause loss of free normal nuclear TDP-43 by promoting its mislocalisation and sequestration into pathological assemblies, thereby perpetuating TDP-43 dysfunction and aggregation in ALS and FTD.

## Results

### Generation and validation of TDP-43-reporter cell lines using CRISPR/Cas9-mediated multiple allele fluorescence tagging of the endogenous *TARDBP* locus

**Fig. 1:**
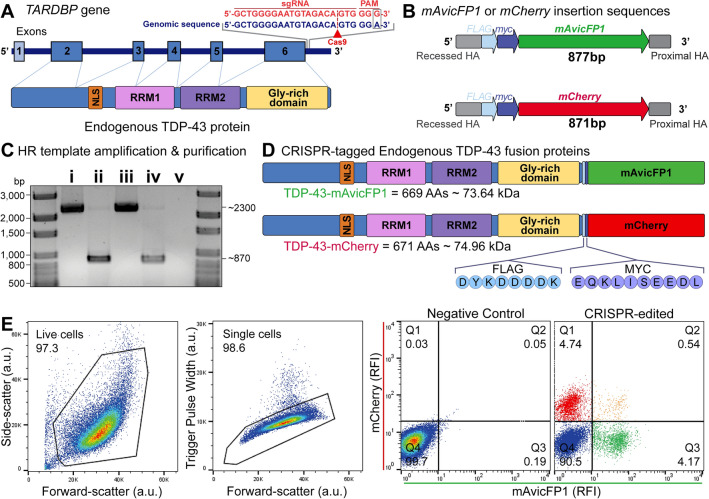
CRISPR/Cas9-mediated gene editing for single and dual insertion of *mAvicFP1* or *mCherry* fluorescent tags to endogenous *TARDBP* C-termini*.* **a** Schematic of endogenous *TARDBP* gene and protein structure, including the single guide RNA (sgRNA) target site and mutated protospacer adjacent motif (PAM; red text) that guides Cas9-mediated (red arrowhead) DNA double-stranded breaks (DSBs) at the C-terminus of *TARDBP* (blue text). **b** Schematic of DNA donor insertion sequence design and **c** DNA electrophoresis of **i** *mAvicFP1* plasmid, **ii** *mAvicFP1* PCR amplicon donor template, **iii** *mCherry *plasmid, **iv** *mCherry *PCR amplicon donor template, and **v** non-template control. **d** Schematic of the expected protein products of CRISPR/Cas9-mediated gene insertion, endogenous TDP-43-mAvicFP1 and TDP-43-mCherry fusion proteins, including amino acid (AA) length and predicted protein size. **e** Fluorescence-activated cell sorting of nucleoporated HEK293T cells. Left to right: plot of forward- and side-scatter resolves live cells (a.u., arbitrary units), plot of forward-scatter and trigger pulse width resolves single cells. Representative plots of mAvicFP1 and mCherry relative fluorescence intensity (RFI) from negative control and polyclonal CRISPR-edited samples. Quadrant gating was set using the negative control

To visualise and quantify the spatiotemporal dynamics of endogenous TDP-43, we inserted fluorescent tags to the *TARDBP* C-terminus in single or multiple alleles using CRISPR/Cas9-mediated homology-directed repair (Fig. 1a). We exploited the HEK293T hypertriploid karyotype [66] (i.e., multiple copies of the *TARDBP* allele), to generate three distinct types of fluorescently-tagged *TARDBP* cell lines, by mixing *mAvicFP1* and *mCherry* DNA donor templates in a single reaction (Fig. 1b,c; Supplementary Fig. S1). mAvicFP1 is a recently discovered fluorescent-protein, derived from *Aequorea victoria*, which has 80% homology to traditional GFP, but produces brighter chromophores [67]. Our approach resulted in a polyclonal population of cells with expression of TDP-43-mAvicFP1 or TDP-43-mCherry, either alone or in combination, from donor integration across multiple *TARDBP* alleles (Fig. 1d). Fluorescence-activated cell sorting revealed 4.74% of cells were mAvicFP1-positive, 4.17% were mCherry-positive, and 0.54% were both mAvicFP1- and mCherry-positive, or ‘dual-tagged’, with the remainder (90.5%) unedited (Fig. 1e).

**Fig. 2:**
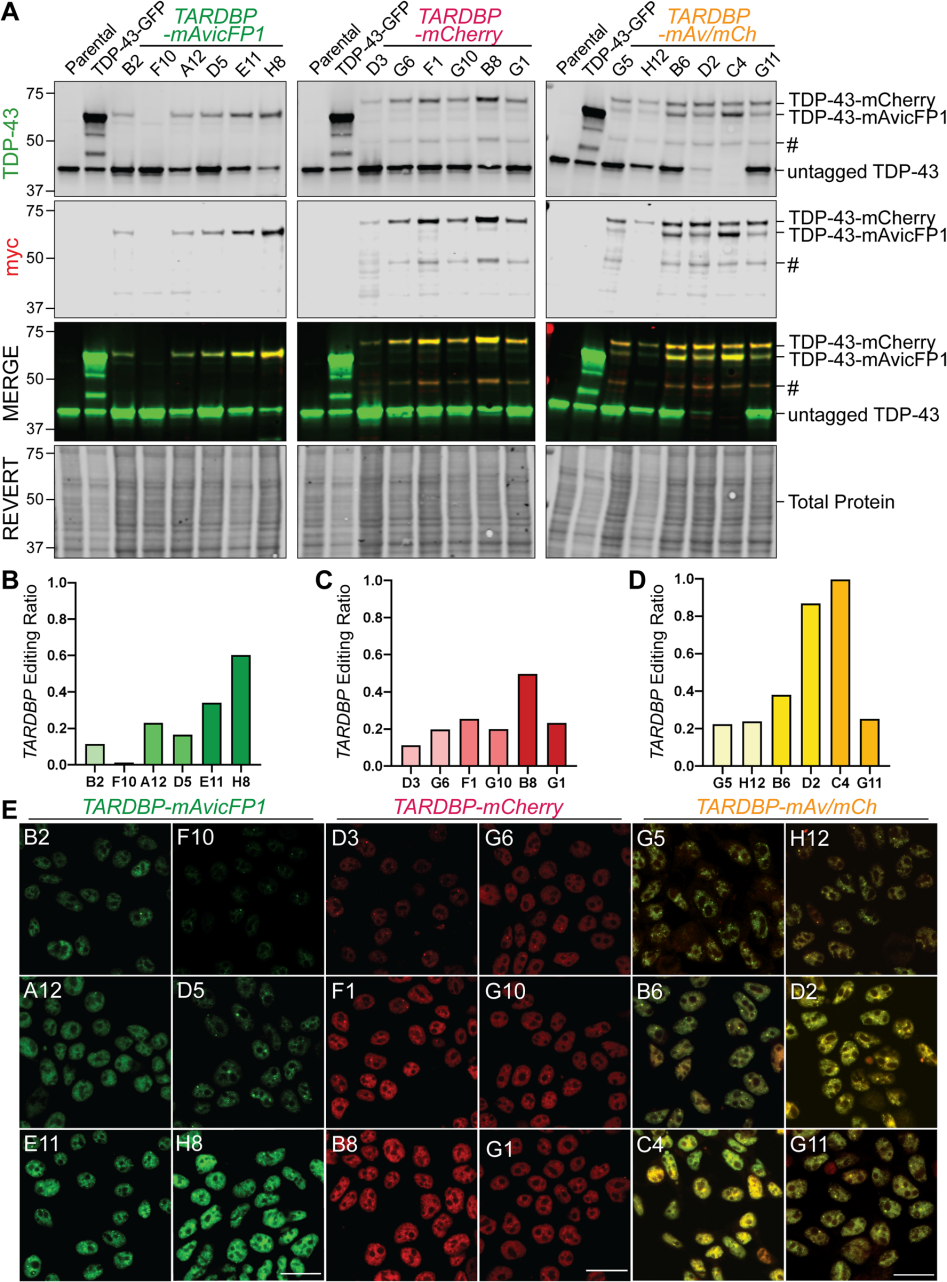
Expression of high-molecular weight TDP-43 protein verifies successful CRISPR/Cas9-mediated gene insertion of *mAvicFP1* and/or *mCherry* to *TARDBP* alleles. Six monoclonal cell lines of varying intensity were selected for validation of mAvicFP1, mCherry, or dual-tag insertion. **a** Immunoblotting for TDP-43 and myc revealed the abundance of ‘untagged’ (43 kDa) and ‘tagged’ TDP-43 (72–74 kDa), which were quantified by densitometry analysis. CRISPR-editing ratios for **b** *TARDBP-mAvicFP1*, **c** *TARDBP-mCherry*, or **d** *TARDBP-mAvicFP1/-mCherry* monoclonal cell lines were calculated by dividing the total protein-normalised signals of high-molecular weight tagged TDP-43 by the combined signals of all TDP-43 bands, as described in Supplementary Fig. S2. ‘#’ Represents a lower-molecular-weight band positive for endogenous mCherry-tagged TDP-43. **e** Representative confocal images captured at 63 × magnification depict relative fluorescence intensities of *TARDBP*-tagged cell lines and clone names are indicated in the upper left corner. Note that *TARDBP-mAv/mCh* images are displayed as a merge of separate mAvicFP1 and mCherry channels (scale = 20 µm)

To validate the efficiency and accuracy of multi-allelic CRISPR/Cas9-mediated gene insertion, we analysed RIPA-soluble protein and genomic DNA from six candidate monoclonal cell lines with a range of fluorescence intensities for each of *TARDBP*-*mAvicFP1*, *TARDBP*-*mCherry*, or dual-tagged lines. Immunoblotting for total TDP-43 revealed multiple bands that differed in molecular weight, including ‘untagged’ TDP-43 (43 kDa) or high-molecular-weight ‘tagged’ TDP-43-mAvicFP1 (~ 72 kDa) and TDP-43-mCherry (~ 74 kDa) fusion proteins (Fig. 2a). Probing for the myc tag, which was inserted in the linker sequence, confirmed that higher-molecular-weight TDP-43 bands were CRISPR-edited fluorescently tagged fusion proteins. To estimate the proportion of *TARDBP* loci with or without insertion, we calculated an ‘editing ratio’ for each clone, by dividing the combined densitometry signals of 72/74 kDa ‘tagged’ TDP-43 by the sum of all TDP-43 bands, including ‘untagged’ 43 kDa species (Fig. 2b–d, Supplementary Fig. S2). For example, an editing ratio of 1.0 indicates complete CRISPR insertion across all alleles, as seen with *TARDBP-mAvicFP1/-mCherry* clone C4 (Fig. 2d), which also showed the highest fluorescence intensity (Fig. 2e). *TARDBP*-mCherry clone B8 had an editing ratio of ~ 0.5, suggesting that half of *TARDBP* alleles possessed an *mCherry* tag (Fig. 2c). *TARDBP* editing ratio generally correlated with fluorescence intensity of monoclonal cell lines observed by confocal microscopy, which also revealed the expected sub-nuclear distribution of TDP-43 with exclusion from nucleoli (Fig. 2e). We observed that not all clones expressed equal total endogenous TDP-43 levels as measured by immunoblot and speculate that differential allele-specific expression may underly clone-to-clone variability. Finally, we performed Sanger sequencing on genomic DNA from top candidate monoclonal cell lines of the highest editing efficiency, namely *TARDBP-mAvicFP1(H8)*, *TARDBP-mCherry(B8)*, *TARDBP-mAv/-mCh(C4)*. Indeed, complete DNA donor templates, including *FLAG-myc* linker and *mAvicFP1* or *mCherry* sequences, were precisely inserted at the intended C-terminal *TARDBP* Exon 6 locus for all clones (Supplementary Fig. S3). These three validated lines were used for subsequent downstream analyses.

### Fluorescently-tagged endogenous TDP-43 exhibits characteristic protein localisation, self-assembly, and mobility dynamics under physiological conditions or oxidative stress

**Fig. 3:**
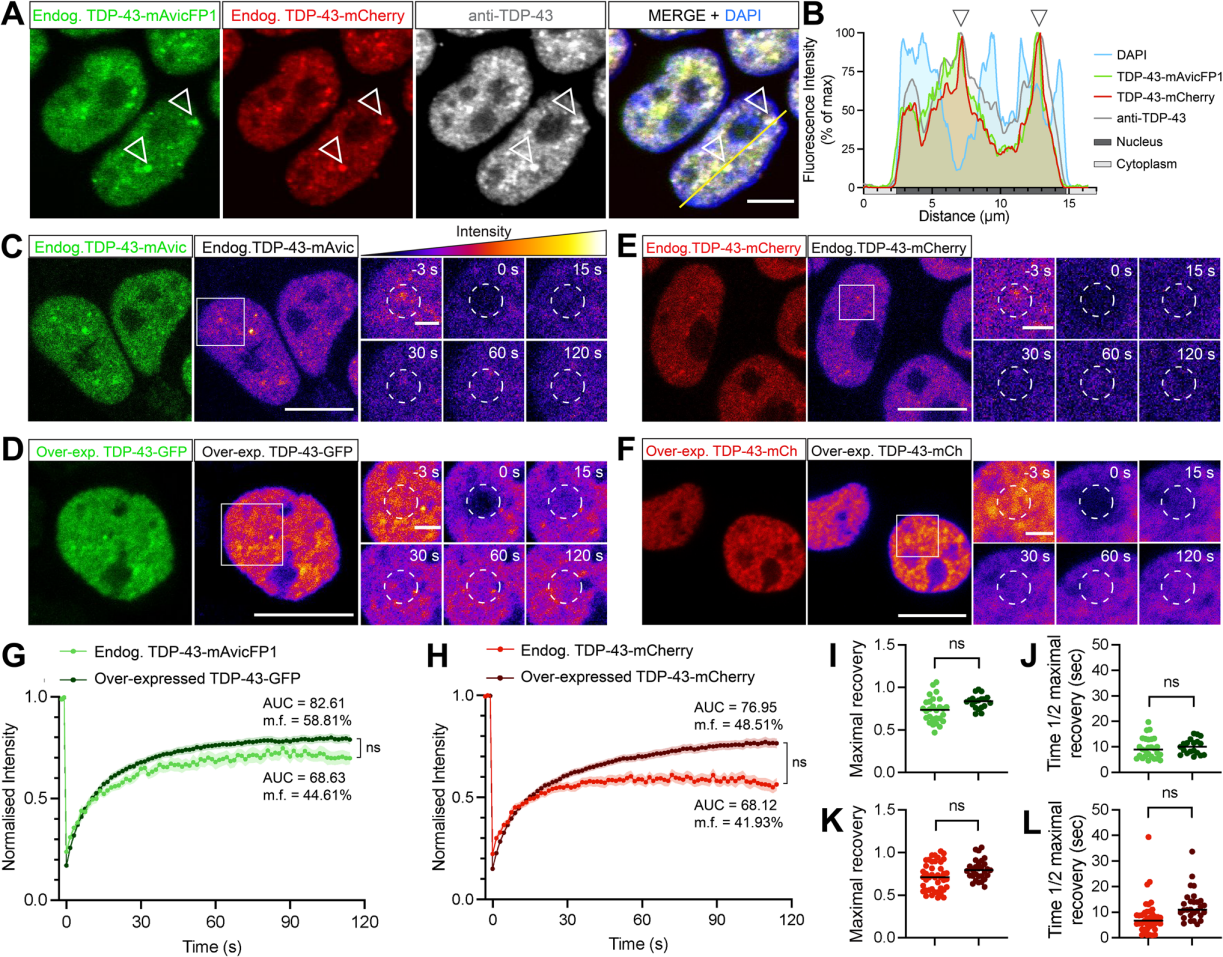
CRISPR-tagged endogenous TDP-43-mAvicFP1/-mCherry fusion proteins demonstrate nuclear localisation and high protein mobility under physiological conditions. **a** CRISPR-tagged TDP-43-mAvicFP1 and TDP-43-mCherry proteins co-localise strongly with an antibody that detects full-length TDP-43, exhibiting diffuse but slightly speckled nuclear distribution, indicated by arrowheads. Scale bar = 5 µm. **b** Intensity profile displaying spatial alignment of TDP-43-mAvicFP1, TDP-43-mCherry and anti-TDP-43 immunofluorescence signals within nuclei. **c**–**f** FRAP was performed to quantify the mobility of nuclear endogenous TDP-43 in *TARDBP-mAvicFP1* cells or *TARDBP-mCherry* cells. The two large left panels (scale bar = 10 µm) show initial snapshots of **c** endogenous TDP-43-mAvicFP1, **d** over-expressed TDP-43-GFP, **e** endogenous TDP-43-mCherry, and **f** over-expressed TDP-43-mCherry, depicted with the correct emission wavelength (green or red) on the left and pseudocoloured “fire” LUT to visualize the scale of intensity on the right. Image intensities were re-scaled between conditions to allow for side-by-side comparison of fluorescence recovery. Smaller panels display a zoomed-in view of the bleached region of interest outlined by white dashed circles, captured at −3, 0, 15, 30, 60 and 120 s post-bleaching (scale bar = 2 µm). **g**, **h** Normalised intensity over time post-bleaching, with data points along line graphs representing mean values of *n* = 3 biologically independent repeats, each including at least 5 cells on average, and the shaded area denotes standard error about the mean. Mobility fraction (m.f.) and area-under-the-curve (AUC) values were calculated for the recovery profile of each individual cell and averaged across each biological replicate before taking the overall means as displayed on the graphs. AUC was compared between groups for statistical comparison of overall recovery profile via two-tailed *t*-tests (ns = not significant; *P* > 0.05). **i**, **k** Maximal fluorescence recovery level relative to pre-bleaching intensity, for mAvicFP1/GFP and mCherry, respectively. **j**, **l** Time taken to recover to half of the maximal level, for mAvicFP1/GFP and mCherry, respectively. Each point represents individual cell FRAP recordings across multiple cells in each replicate and black lines represent mean of *n* = 3 independent repeats as above, which were analysed via two-tailed *t*-tests (ns = not significant; *P* > 0.05)

To determine whether characteristic changes in TDP-43 localisation, dynamics, and aggregation under physiological conditions and disease-associated stress were recapitulated in our CRISPR-tagged endogenous TDP-43 cell lines, we performed a series of imaging assays. We further validated normal nuclear localisation and complete colocalisation of the inserted mAvicFP1/mCherry fluorescent tags and endogenous TDP-43, by immunostaining double-tagged *TARDBP-mAvicFP1/-mCherry(C4)* cells with an antibody that detects full-length TDP-43 (Fig. 3a,b). Fluorescence recovery after photobleaching (FRAP) in single-tagged *TARDBP-mAvicFP1(H8)* or *TARDBP-mCherry(B8)* lines demonstrated similar overall mobility dynamics compared to commonly studied diffuse nuclear exogenous over-expressed wild-type (WT) TDP-43-mGFP or TDP-43-mCherry (Fig. 3c–l). Area-under-the-curve (AUC) and mobility fraction (m.f.) analyses, indicating the overall proportion of highly mobile proteins within the nucleus, showed a trend for decreased mobility of endogenous fusion proteins relative to their over-expressed forms but these did not reach statistical significance (AUC = 67.80 and 82.61, m.f. = 44.61 and 58.81%, respectively, for -mAvicFP1/GFP [Fig. 3g]; AUC = 68.12 and 76.95, m.f. = 41.93 and 48.51%, respectively, for -mCherry [Fig. 3h]). Maximal fluorescence recovery levels relative to pre-bleaching intensity were not significantly different between endogenous and over-expressed forms of TDP-43-mAvicFP1/GFP (0.74 and 0.83, respectively [Fig. 3i]), or endogenous and over-expressed forms of TDP-43-mCherry (0.76 and 0.80, respectively [Fig. 3k]). Regarding recovery rate kinetics, there were no significant differences in time taken to reach half maximal recovery between endogenous and over-expressed TDP-43 (8.71 s and 9.81 s, respectively, for -mAvicFP1/GFP [Fig. 3j]; 8.5 s and 11.49 s, respectively, for -mCherry [Fig. 3l]). Therefore, CRISPR-tagged endogenous TDP-43 proteins demonstrate similar properties to commonly studied over-expressed TDP-43 fusion proteins, thereby validating these cell lines for further studies of TDP-43 protein dynamics.

**Fig. 4:**
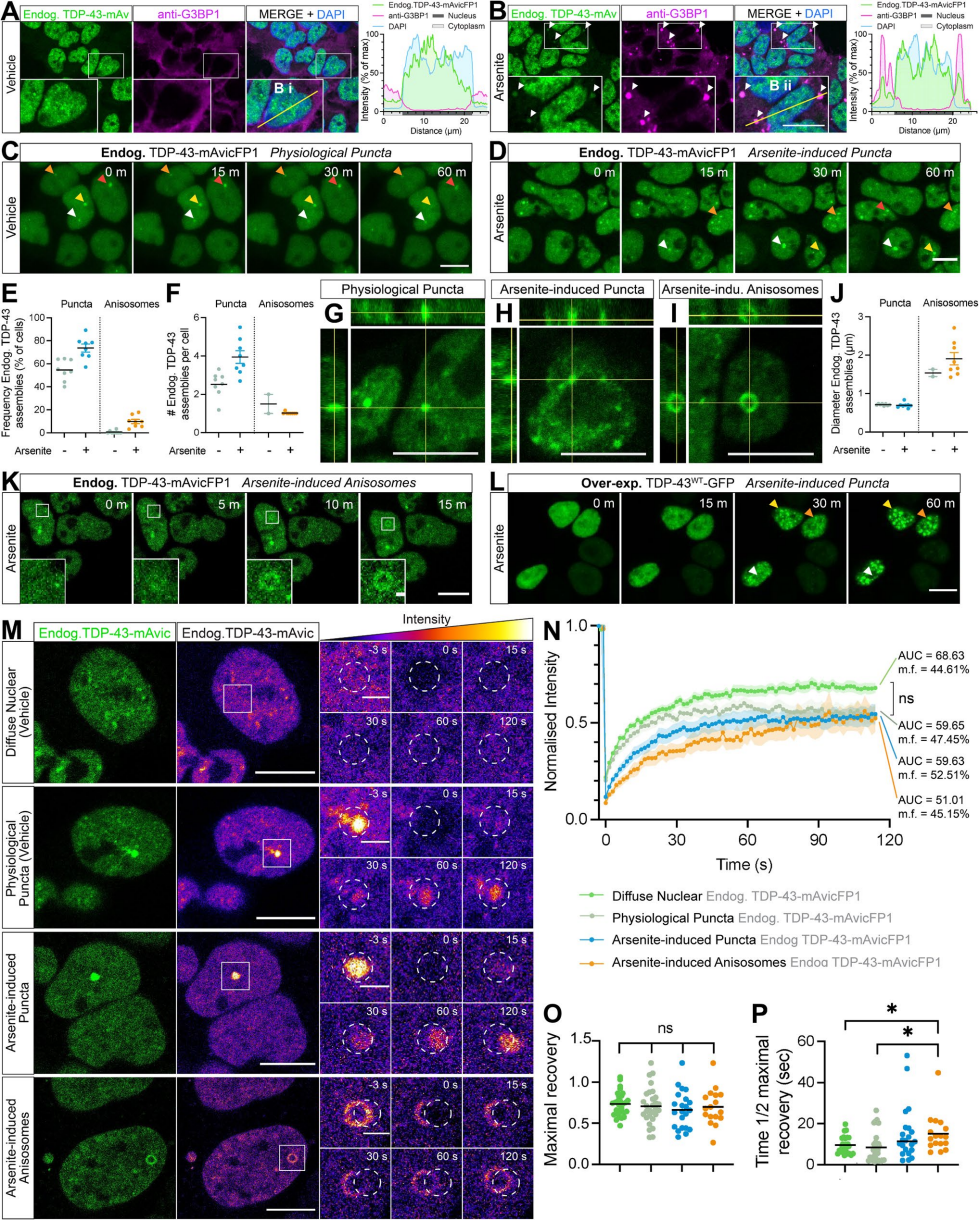
Endogenous CRISPR-tagged normal nuclear TDP-43 undergoes physiological or stress-induced self-assembly via liquid–liquid phase separation to form cytoplasmic stress granules or dynamic nuclear puncta and anisosomes in real-time. **a**, **b** Representative confocal micrographs with Z-stack maximum projection and intensity profiles of anti-G3BP1 labelling and TDP-43-mAvicFP1 in HEK293T:*TARDBP-mAvicFP1* cells treated with vehicle or 50 µM sodium arsenite for 1 h. Inset zoomed view of cells measured for fluorescence intensity profiles; scale = 10 µm. **c** Live-cell imaging displays formation of stable physiological puncta with 1 h vehicle treatment, or **d** increased formation of nuclear puncta induced by 50 µM arsenite. **e** Frequency of cells with endogenous TDP-43-mAvicFP1 nuclear puncta and **f** number of puncta per cell. Data points represent the mean of all cells within an image, totalling 8 images per group. **g** 3-dimensional orthogonal projections of physiological nuclear puncta **h** arsenite-induced puncta, and **i** arsenite-induced anisosomes comprising endogenous TDP-43-mAvicFP1. **j** Diameter size in µm of puncta and anisosomes. Data points representing ‘per-image’ means. **k** Live-cell imaging of endogenous TDP-43-mAvicFP1 anisosome formation with 50 µm arsenite treatment. **l** Live-cell imaging of parental HEK293T cells over-expressing TDP-43^WT^-GFP with 50 µm arsenite treatment; scale large panels = 10 µm; zoomed panels = 1 µm. **m** FRAP quantification of endogenous TDP-43-mAvicFP1 mobility within distinct nuclear assemblies. Two large left panels depict endogenous TDP-43-mAvicFP1 singal with correct emission (green) on the left and pseudocoloured “fire” LUT to visualise intensity scale on the right; scale = 10 µm. Image intensities re-scaled between conditions to allow for side-by-side comparison of fluorescence recovery. Smaller panels display zoomed view with photo-bleached ROI outlined by white dashed circles, captured at −3, 0, 15, 30, 60 and 120 s post-bleaching; scale = 2 µm. **n** FRAP analysis displays normalised intensity during recovery over 120 s, along with mobility fraction (m.f.) values and area-under-the-curve (AUC) averaged for each cell, which was used for statistical comparison. Coloured lines represent mean of *n* = 3 biologically independent repeats, each striving for at least 5 cells, with shaded areas denoting standard error about the mean. **o** Maximal fluorescence recovery level relative to pre-bleaching intensity, and **p** time taken to recover to half of the maximal level for each of the TDP-43-mAvicFP1 assemblies. The same colour scheme as that of the intensity line graphs was used, with black lines representing the mean across *n* = 3 biologically independent repeats, and each point denoting individual cells. All data analysed with one-way ANOVA multiple comparisons with Tukey’s post hoc test, relative to the ‘diffuse nuclear endogenous TDP-43-mAvicFP1’ control (*P < 0.05)

Liquid–liquid phase separation (LLPS) is a key feature of physiological TDP-43 function, but aberrant or chronic LLPS-mediated TDP-43 assembly may seed aggregation. As TDP-43 LLPS has commonly been studied using over-expression constructs, truncated mutants, optogenetic clustering methods, or recombinant proteins in vitro, we sought to characterise LLPS structures comprising endogenous normal TDP-43 protein in live cells. As an essential RNA-binding protein, TDP-43 has previously been shown to be a major component of SGs that assemble under oxidative stress [32, 48, 59, 68]. Indeed, fixed-cell immunostaining for the SG marker, G3BP1, in *TARDBP-mAvicFP1* cells under physiological conditions demonstrated absence of cytoplasmic SGs with diffusely distributed endogenous TDP-43 in the nucleus (Fig. 4a). As expected, treatment with the oxidative stressor, 50 µM sodium arsenite, for 1 h induced formation of distinct rounded G3BP1-positive and endogenous TDP-43-positive SGs of ~ 2–4 µm in diameter (Fig. 4b). Live-cell imaging of vehicle-treated cells also revealed small nuclear physiological endogenous TDP-43 ‘puncta’, defined as dynamic ‘droplet-like’, homogenous, rounded, particles of 0.5–2.5 µm in diameter (Fig. 4c, g). Arsenite-mediated oxidative stress increased the percentage of cells with nuclear endogenous TDP-43 puncta by approximately 20%, and the number of puncta per cell by 50% (Fig. 4d–f, h). However, arsenite-induced cytoplasmic endogenous TDP-43 SGs could not be detected under these live-cell imaging parameters, likely because CRISPR-tagged TDP-43-mAvicFP1 protein at endogenous levels exhibits low fluorescence intensity overall, and only a small portion of the cellular TDP-43 pool is expected to be recruited to SGs [69]. Endogenous TDP-43-mAvicFP1 in SGs was therefore only resolved in fixed cells with longer exposure, higher laser power imaging.

Interestingly, arsenite treatment in live *TARDBP-mAvicFP1* cells also induced formation of other LLPS assemblies reminiscent of the recently characterised TDP-43 ‘anisosomes’, which are spherical shell-like RNA-deficient structures with a core void of TDP-43 and of ~ 1–4 µm in diameter (larger than the small dense puncta analysed separately above) (Fig. 4i–k). Anisosome formation has been primarily characterised with acetylation-mimicking or RNA-binding-ablated TDP-43 mutants, which encapsulate a liquid core of phase-separated heat-shock protein 70 (Hsp70) that maintains structure and solubility [25]. Anisosomes containing endogenous normal RNA-binding competent TDP-43 formed in 10% of arsenite-treated cells, wherein most cells contained just one anisosome, compared to 0.5% of vehicle-treated cells exhibiting spontaneous anisosome formation (Fig. 4e, f). Treatment with sodium arsenite yielded anisosomes that were on average 2 µm in diameter (Fig. 4j). Endogenous TDP-43 anisosomes formed rapidly (within 15 min, Fig. 4k) and remained stable throughout arsenite treatment (Supplementary Fig. S4A). To determine whether over-expressed wild-type TDP-43 similarly formed anisosomes, we also analysed exogenous TDP-43^WT^-GFP LLPS assemblies in parental HEK293T cells treated with arsenite. Oxidative stress increased formation of bright exogenous TDP-43^WT^-GFP puncta over time, but remarkably, did not induce anisosome formation (Fig. 4l). This difference between endogenous and over-expressed TDP-43 self-assembly may highlight the dependency of TDP-43 self-assembly patterns on protein abundance, indicating that abnormally high levels of TDP-43 protein may inhibit anisosome formation, instead possibly favouring other forms of LLPS or aggregation. This suggests that LLPS-mediated formation of stress-induced anisosomes and other dynamic structures composed of wild-type TDP-43 may be best studied in models that visualise physiological TDP-43.

We next used FRAP to determine the relative mobility dynamics of the distinct nuclear assemblies of endogenous TDP-43, namely physiological puncta, arsenite-induced puncta, and arsenite-induced anisosomes (Fig. 4m). Endogenous TDP-43 within each of these assemblies exhibited dynamic exchange and/or recruitment of TDP-43 molecules over time, showing no significant differences in area-under-the-curve (AUC), or mobility fraction (m.f.) analyses (Fig. 4n), and comparable maximal fluorescence recovery levels, to diffusely distributed nuclear TDP-43 (Fig. 4o). However, endogenous TDP-43 anisosomes showed a significantly decreased rate of recovery, measured as the time to reach half maximal intensity post-bleaching (t_1/2_ = 19.53 s), compared to diffuse and physiological puncta of endogenous TDP-43 (t_1/2_ = 7.96 s) (Fig. 4p).

Together, these results demonstrate that CRISPR/Cas9-tagged endogenous TDP-43-mAvicFP1 and TDP-43-mCherry proteins exhibit characteristic TDP-43 localisation and mobility, however there were some key differences in the propensity for LLPS assembly under oxidative stress compared to over-expressed wild-type TDP-43. Notably, we showed that under oxidative stress, over-expressed TDP-43^WT^-GFP demonstrated increased formation of nuclear puncta compared to endogenous TDP-43-mAvicFP1, but did not form anisosomes which were observed in 10% of *TARDBP-mAvicFP1* cells (Fig. 4l). This indicates that over-expressed proteins may not always faithfully recapitulate self-association states of TDP-43, which may be influenced by protein concentration. Endogenous TDP-43 anisosomes were dynamic, yet demonstrated a slower fluorescence recovery rate than diffuse or physiological puncta of endogenous TDP-43 (Fig. 4m–p), indicating decreased rate of recruitment. Therefore, both cellular stress conditions and the distinct structures of phase-separated TDP-43 assemblies affects the availability and mobility dynamics of normal nuclear TDP-43.

### Pathological accumulation of RNA-binding-ablated or acetylation-mimic TDP-43 sequesters and depletes free normal nuclear TDP-43 in a concentration-dependent manner

**Fig. 5:**
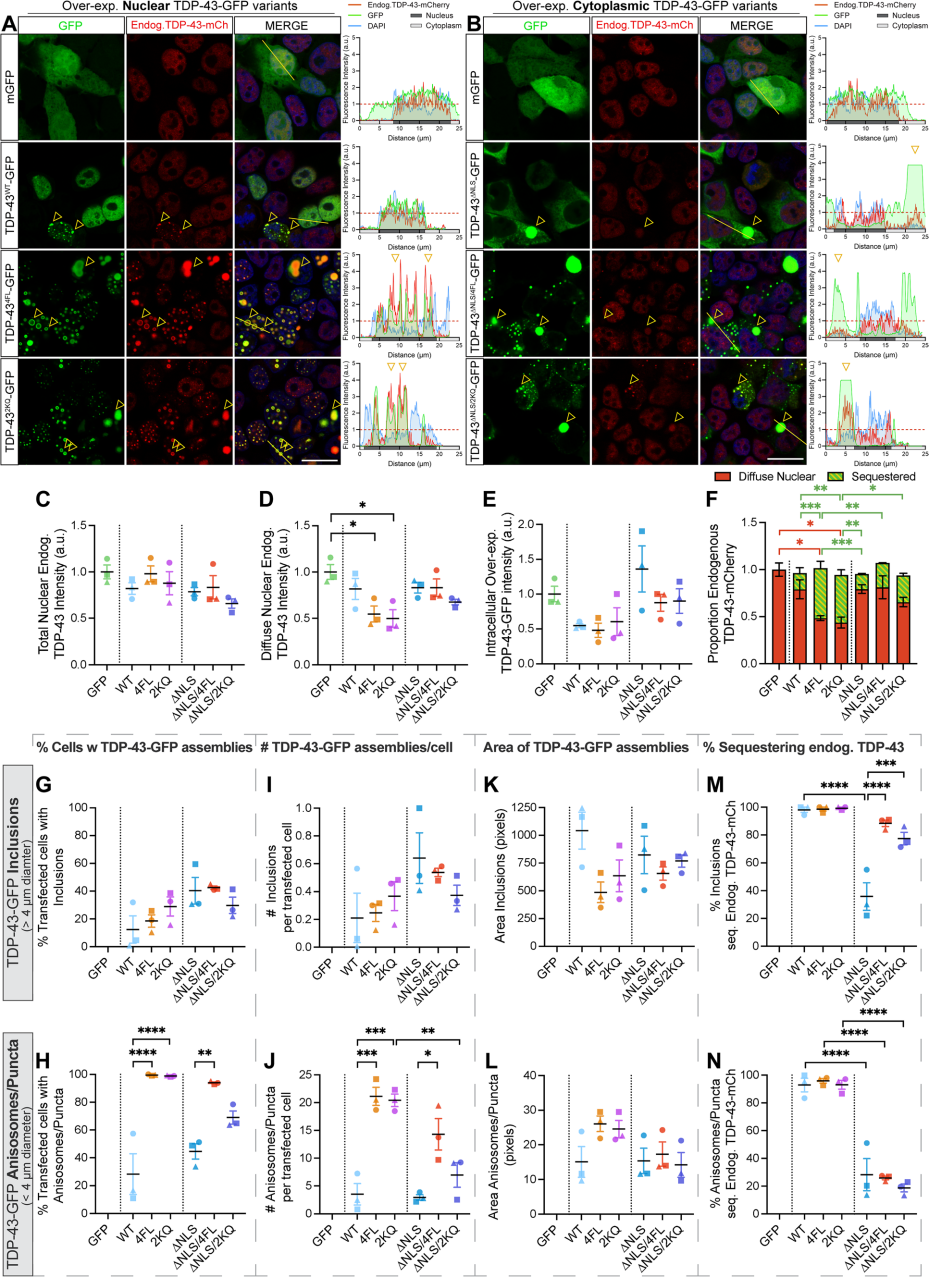
Nuclear or cytoplasmic RNA-binding-ablated or acetylation-mimicking TDP-43 decreases endogenous TDP-43 levels and recruits endogenous TDP-43 to puncta and inclusions. **a**, **b** Confocal images of HEK293T:*TARDBP-mCherry* cells over-expressing **a** nuclear or **b** cytoplasmic TDP-43-GFP variants after 48 h, and assemblies that sequester endogenous TDP-43-mCherry (yellow arrowheads). Right-hand panels display relative fluorescence intensity profiles of example cells (left-to-right along yellow line), with yellow arrowheads demonstrating position of sequestering assemblies. Red lines denote the mean intensity level of each channel in GFP-transfected control cells, to which other intensities are normalised. **c**–**f** CellProfiler image segmentation analysis was used to quantify intensities of **c** total nuclear or **d** diffuse nuclear endogenous TDP-43-mCherry proteins, and **e** intracellular levels of over-expressed TDP-43-GFP protein variants in transfected cells. Each point represents the mean relative fold change from GFP-transfected control cells, sampling cells from 8 regions-of-interest at × 63 magnification, for each of *n* = 3 biologically independent repeats, denoted by different point shapes. **f** Stacked bar graph demonstrating the proportions of endogenous TDP-43 diffusely distributed in the nucleus (red) or sequestered into nuclear or cytoplasmic TDP-43-GFP aggregates (green). **g**–**n** CellProfiler image segmentation was used to quantify parameters of aggregates formed by over-expression of TDP-43-GFP variants. Inclusions were defined as large bright speckles with intensity above 60% of saturation point and minimum diameter of 4 µm. Anisosomes/puncta were defined as speckles with intensity above 25% of saturation point and diameter of 0.5–4 µm. **g**, **h** Proportion of transfected cells with inclusions or puncta. **i**, **j** Number of inclusions or puncta per transfected cell. **k**, **l** Area of inclusions or puncta. **m**, **n** Proportion of inclusions or puncta sequestering endogenous TDP-43, respectively. Inclusions or puncta sequestering endogenous TDP-43-mCherry defined as those with endogenous TDP-43-mCherry intensity above the lower quartile intensity of diffusely-distributed nuclear endogenous TDP-43-mCherry in GFP-transfected control cells. Cell numbers and replicates as per (C-E). Statistically significant differences between the means were analysed as one-way ANOVA multiple comparisons with Tukey’s post hoc test (*P < 0.05, **P < 0.01, ***P < 0.001, ****P < 0.0001)

Another key feature of disease is the depletion of normal nuclear TDP-43, which is observed in cells that exhibit pathological aggregation, but can also independently mediate neurodegeneration. To determine whether the progressive accumulation of different pathological TDP-43 assemblies causes loss of free nuclear TDP-43 in single cells, we over-expressed nuclear wild-type (WT), RNA-binding-ablated (4FL; F147L/F149L/F229L/F231L) or acetylation-mimic (2KQ; K145Q/K192Q) TDP-43 mutants, as well as cytoplasmically-mislocalised variants of each mutant, harbouring disrupted nuclear localisation signals (∆NLS; K82A/R83A/K84A), in HEK293T:*TARDBP-mCherry* cell lines (Fig. 5a, b). In duplicate experiments, parental HEK293T cells lacking CRISPR-tagged endogenous TDP-43-mCherry transfected with TDP-43-GFP variants were used as imaging controls to ensure no cross-over of fluorescence emission and for setting intensity thresholds during quantitative image analysis (Supplementary Fig. S5). Confocal imaging and unbiased automated CellProfiler image segmentation analysis (Supplementary Fig. S6) demonstrated that over-expression of TDP-43-GFP variants led to slight, but not statistically significant, decreases in total nuclear endogenous TDP-43-mCherry signal (Fig. 5c), compared to GFP-transfected control cells.

We observed that the distribution of endogenous TDP-43-mCherry was altered and co-localised with mutant TDP-43-GFP assemblies formed by each of the nuclear or cytoplasmic variants, with different tendencies (yellow arrowheads and intensity profiles; Fig. 5a, b). This indicated sequestration, which showed an extremely dramatic effect in cells over-expressing nuclear RNA-binding ablated (4FL) or acetylation-mimicking (2KQ) mutants that formed numerous TDP-43 anisosomes. We performed detailed image segmentation analysis of individual TDP-43-GFP assemblies, categorised into either ‘inclusions’ or ‘anisosomes/puncta’ based on size (Supplementary Fig. S6). ‘Inclusions’ were defined as solid, fluorescence-saturated, large amorphous structures of > 4 µm diameter (Supplementary Fig. S6). Both ‘anisosomes’ and ‘puncta’ are categorised here as smaller rounded structures of 0.5–4 µm diameter, but critically differ in their localisation and morphology (Supplementary Fig. S6). ‘Anisosomes’ were dynamic spherical shell-like structures with a core void of TDP-43 formed in the nucleus by RNA-binding-ablated 4FL and acetylation-mimicking 2KQ TDP-43 mutants. ‘Puncta’ were ‘droplet-like’, demonstrating homogenous TDP-43 intensity throughout, predominantly observed in the cytoplasm, or occasionally in the nucleus for WT TDP-43. All TDP-43-GFP variants demonstrated variable tendencies for forming large inclusions (Fig. 5g), and the number of inclusions per transfected cell (Fig. 5i), which were slightly higher for cytoplasmic mutants, with no difference in size of inclusions formed (Fig. 5k). However, nuclear RNA-binding-ablated 4FL or acetylation-mimicking 2KQ showed dramatically increased formation (Fig. 5h, j) and size (Fig. 5l) of anisosomes compared to puncta formed by other variants. Our results support previous work [25], showing that nuclear RNA-binding-ablated and acetylation-mimicking TDP-43 consistently form anisosomes, which we observed in ~ 100% of transfected cells, each containing approximately 20 anisosomes (Supplementary Fig. S7B-D).

Specific and rapid sequestration to anisosomes was verified by three-dimensional orthogonal projections and timelapse imaging, illustrating complete colocalisation of endogenous TDP-43-mCherry throughout the anisosome “shell” (Supplementary Fig. S4B, C). Due to technical limitations for antibody penetration of condensed protein assemblies, we were unable to observe endogenous Hsp70 immunostaining within the core of anisosomes that contained endogenous TDP-43 (Supplementary Fig. S4E), as reported previously [25]. However, we verified the Hsp70-dependent nature of anisosome formation and sequestration using an Hsp70 ATPase inhibitor, VER155088, which causes rapid disassembly of acetylation-mimicking TDP-43-GFP anisosomes that led to restoration of diffuse nuclear endogenous TDP-43-mCherry levels and localisation over time (Supplementary Fig. S4D, E).

To determine the relative amount of endogenous TDP-43 sequestered by mutant TDP-43-GFP assemblies, we measured mCherry intensity within areas occupied by inclusions or anisosomes/puncta. In addition, measurement of mCherry intensity inside the nucleus, but excluding assemblies, quantified the functional pool of ‘diffuse’ endogenous TDP-43-mCherry remaining. Over-expression of each of the variants led to slight decreases in levels of diffuse nuclear endogenous TDP-43, but the most dramatic was caused by 4FL or 2KQ mutants, demonstrating a significant 50% depletion (Fig. 5d). We noted cell-to-cell variability in overall levels of over-expressed TDP-43-GFP variants (Fig. 5e), so therefore we conducted linear regressions analysis of mCherry and GFP fluorescent intensities of individual cells to determine the direct relationship between exogenous TDP-43 and diffuse endogenous TDP-43 levels (Supplementary Fig. S7E). We found clear negative correlations between over-expressed TDP-43-GFP and diffuse nuclear endogenous TDP-43-mCherry intensities, indicating a concentration-dependent effect where high intracellular levels of exogenous TDP-43-GFP further depletes diffuse endogenous normal nuclear TDP-43 (Supplementary Fig. S7E). Notably, RNA-binding-ablated 4FL or acetylation-mimicking 2KQ showed a steeper linear regression slope suggesting that these mutants drive greater depletion of diffuse endogenous TDP-43 for a given level of GFP expression compared to WT TDP-43-GFP (Supplementary Fig. S7E). On the other hand, there was a positive correlation between intensity of TDP-43-GFP assemblies and endogenous TDP-43-mCherry sequestered for all mutants investigated (Supplementary Fig. S7F, G). This substantiates specific and concentration-dependent sequestration of endogenous TDP-43-mCherry by pathological TDP-43 structures. Nuclear RNA-binding-ablated 4FL or acetylation-mimicking 2KQ TDP-43-GFP mutations increased the amount of endogenous TDP-43 sequestration (Supplementary Fig. S7H, I), compared to WT TDP-43-GFP for a given expression level (Supplementary Fig. S7F, G). The sum of ‘diffuse’ and ‘sequestered’ protein levels demonstrated that total cellular abundance of endogenous TDP-43 was unaffected, and that this re-distribution into exogenous TDP-43-GFP assemblies accounts for the depletion of free diffuse normal nuclear endogenous TDP-43 (Fig. 5f).

Assemblies sequestering endogenous TDP-43 were categorised as those with TDP-43-mCherry signals above lower quartile intensity of diffuse nuclear endogenous TDP-43-mCherry in GFP-control-transfected cells (Supplementary Fig. S6). Virtually all nuclear inclusions formed by WT, 4FL or 2KQ variants (> 97.6%) sequestered endogenous TDP-43-mCherry, whereas only 35.0% of cytoplasmic ∆NLS inclusions recruited TDP-43-mCherry (Fig. 5m). However, with cytoplasmic RNA-binding ablation or acetylation-mimicking mutations, 88.5% and 77.5% of ∆NLS/4FL or ∆NLS/2KQ TDP-43 inclusions sequestered endogenous TDP-43, respectively (Fig. 5m), indicating that disease-associated pathological changes increase the sequestration of normal nuclear TDP-43 to cytoplasmic inclusions. Finally, WT puncta or anisosomes formed by 4FL and 2KQ variants in the nucleus almost always (> 92.8%) recruited endogenous TDP-43-mCherry (Fig. 5n). In contrast, corresponding cytoplasmic variants showed significantly lower proportion of TDP-43-mCherry sequestration to puncta (∆NLS = 28.3%, ∆NLS/4FL = 26.0%, ∆NLS/2KQ = 18.7%) (Fig. 5n).

These results show that nuclear RNA-binding-ablated or acetylation-mimicking mutant TDP-43 proteins form an increased number and size of anisosomes compared to WT TDP-43-GFP puncta. Nuclear puncta or anisosomes formed by pathological TDP-43-GFP sequester and deplete diffuse nuclear endogenous TDP-43 in a concentration-dependent manner (Fig. 5d, f, Supplementary Fig. S7E). RNA-binding-ablated or acetylation-mimicking mutations in the cytoplasm also increased sequestration of endogenous TDP-43, compared to ∆NLS-only mutants (Fig. 5f, Supplementary Fig S7F-I). Together these findings suggest that ALS- and FTD-relevant changes in TDP-43 RNA-binding capacity or acetylation enhance propensity for self-association into puncta and depletes free nuclear TDP-43. Immunostaining for total TDP-43 also revealed that anti-TDP-43 encircled TDP-43-GFP aggregates but was unable to stain the core, while fluorescent signals from endogenous TDP-43-mCherry featured throughout these structures (Supplementary Fig. S8), as shown previously [25]. Anti-TDP-43 antibodies are unlikely to penetrate inclusions due to the technical limitations caused by the density of these structures. However, the finding of CRISPR-tagged endogenous TDP-43 throughout the inclusions provides evidence that free normal nuclear TDP-43 is not only sequestered to the surface, but likely also ‘co-aggregates’ with pathological TDP-43 proteins, being incorporated early during inclusion formation and potentially interacting with smaller species preceding inclusions [7]. Importantly, sequestration into large inclusions, puncta, or anisosomes may mark critical molecular events by which normal nuclear TDP-43 becomes mislocalised, dysfunctional, and pathological in cells following initiation of TDP-43 aggregation.

### Free normal nuclear TDP-43 is sequestered into acetylation-mimicking TDP-43 inclusions, becoming insoluble and immobile

**Fig. 6:**
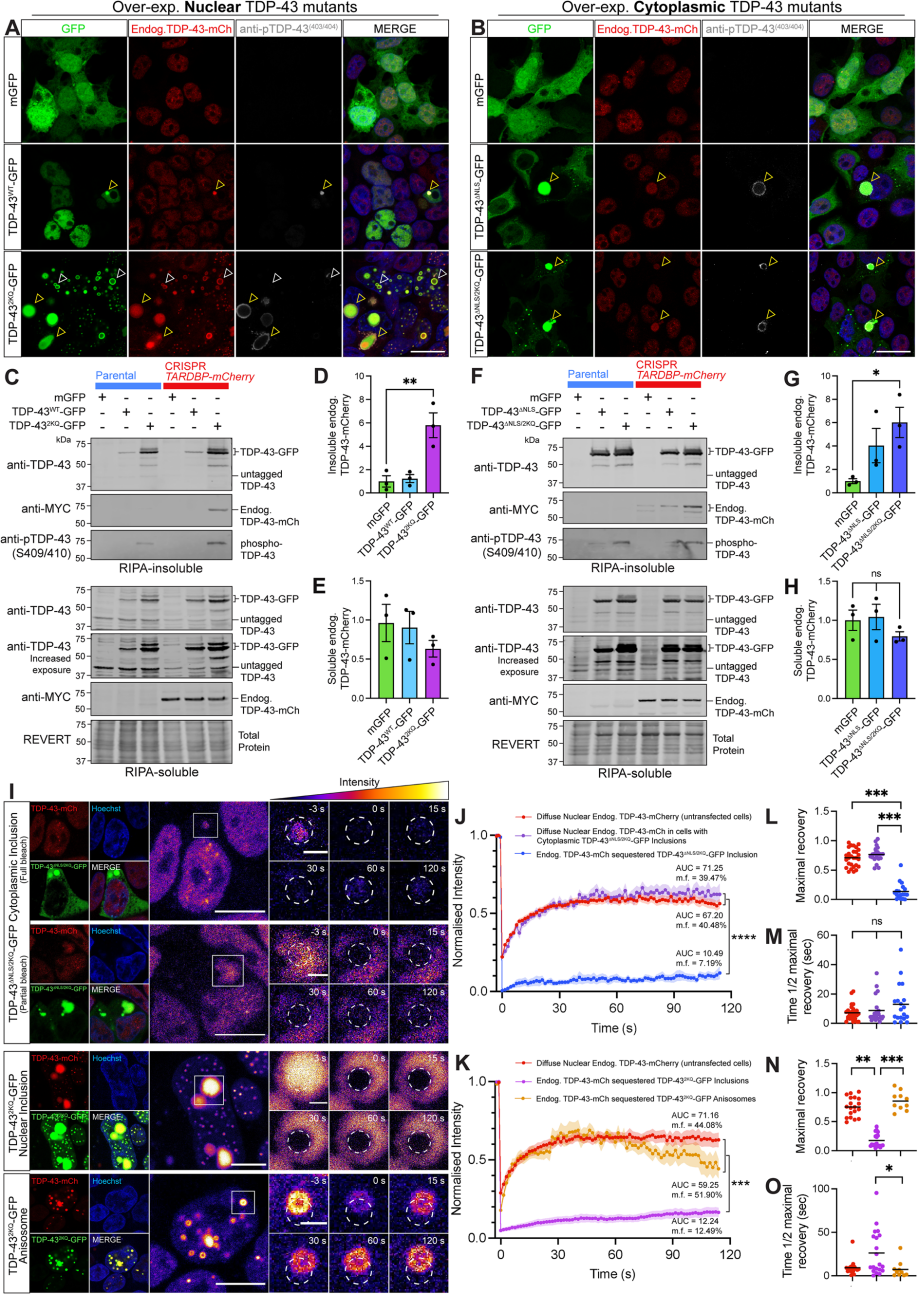
Sequestered endogenous TDP-43 becomes insoluble and immobile in nuclear and cytoplasmic inclusions, however retains high protein mobility when recruited to nuclear anisosomes. **a**, **b** Representative confocal images of CRISPR *TARDBP-mCherry* cells expressing **a** nuclear or **b** cytoplasmic wild-type or acetylation-mimicking mutant TDP-43-GFP, or mGFP control, 48 h post-transfection. Yellow arrowheads indicate TDP-43-GFP inclusions comprising endogenous TDP-43-mCherry and immunostained with phosphorylated TDP-43. White arrowheads indicate acetylated TDP-43 anisosomes which sequestered endogenous TDP-43-mCherry but lacked phosphorylated TDP-43 (scale = 20 µm). **c**, **f** Immunoblotting of CRISPR *TARDBP-mCherry* cells expressing **c** nuclear or **f** cytoplasmic mutants demonstrates changes in endogenous TDP-43-mCherry levels within **d**, **g** RIPA-insoluble and **e**, **h** soluble protein fractions, respectively, quantified by densitometry analysis. **i** FRAP was used to quantify the mobility of endogenous TDP-43-mCherry proteins recruited to distinct nuclear or cytoplasmic structures formed by the over-expression of acetylation-mimic TDP-43-GFP mutants. Scale bars on left-hand panels = 10 µm; zoomed right-hand panels = 2 µm. Pseudocolouring demonstrates changes in endogenous TDP-43-mCherry intensity, which was quantified within the bleaching region-of-interest (white dotted circle) for 120 s post-bleaching. FRAP analysis of the normalised intensity during the recovery period for cells expressing **j** cytoplasmic or **k** nuclear acetylation-mimic mutant TDP-43-GFP, along with mobility fraction (m.f.) values and area-under-the-curve (AUC) averaged for each cell, which was used for statistical comparison. **l**, **n** Maximal fluorescence intensity observed at the final time-point (120 s), and **m**, **o** time taken to recover to half of the maximal fluorescence level for nuclear and cytoplasmic TDP-43 mutants respectively. Same colour scheme as the intensity line graphs, with each point representing a single cell value, and the black line representing the mean across *n* = 3 biologically independent repeats. Statistically significant differences between the means were analysed using one-way ANOVA multiple comparisons with Tukey’s post hoc test (*P < 0.05; **P < 0.01)

As acetylation, along with other PTMs, is a key feature of TDP-43 pathology in ALS and FTD, we next sought to determine whether sequestration of endogenous TDP-43 to acetylation-mimicking mutant TDP-43 structures altered its solubility or mobility, indicating dysfunction and pathological transition. We performed phospho-TDP-43 (403/404) immunostaining on *TARDBP-mCherry* cells expressing nuclear or cytoplasmic acetylation-mimicking TDP-43-GFP proteins to further characterise the sequestration of endogenous TDP-43 into puncta and inclusions. Confocal imaging demonstrates that a majority of large nuclear or cytoplasmic acetylation-mimicking mutant TDP-43 inclusions that sequester normal TDP-43-mCherry were highly phosphorylated (Fig. 6a, b, yellow arrowheads). Smaller cytoplasmic puncta formed by acetylated TDP-43 showed less TDP-43-mCherry sequestration and were occasionally phosphorylated (Fig. 6b). Nuclear anisosomes that readily recruited free normal nuclear TDP-43 were never phosphorylated (Fig. 6a, yellow arrowheads). The tendency for large, phosphorylated, acetylation-mimicking TDP-43 inclusions to recruit endogenous TDP-43 prompts investigation to determine whether this sequestration constitutes a pathological transition of normal TDP-43 protein, leading to insolubility and immobility.

We therefore analysed the levels of detergent-soluble and -insoluble levels of normal endogenous TDP-43-mCherry in cells over-expressing these aggregation-prone TDP-43-GFP mutants (Fig. 6c, f). Over-expression of either nuclear or cytoplasmic acetylation-mimicking TDP-43-GFP resulted in high levels of insoluble phospho-TDP-43, which correlated with significant six-fold increases of insoluble endogenous TDP-43 levels, detected by immunoblotting for the myc tag incorporated as a linker for mCherry in CRISPR editing (Fig. 6d, g). Soluble endogenous TDP-43-mCherry levels were not significantly altered (Fig. 6e, h). These results were replicated using a suite of myc-tagged TDP-43 mutants to show that the formation of assemblies, phosphorylation of inclusions, and sequestration of endogenous TDP-43 into the insoluble protein fraction is independent of the fluorescent tag on the over-expression constructs (Supplementary Fig. S9). Therefore, large, phosphorylated pathology-mimicking nuclear and cytoplasmic inclusions formed by acetylation-mimicking TDP-43 sequester normal TDP-43 and render it insoluble.

To further investigate the concentration-dependent effects of exogenous TDP-43-GFP over-expression on inclusion size and phosphorylation status of individual assemblies, we titrated increasing levels of mutant TDP-43. HEK293T:TARDBP-mCherry cells were transfected with ‘low’, ‘medium’, or ‘high’ amounts of DNA (0.25 μg, 0.5 μg, 1 μg, respectively, per coverslip) for the expression of nuclear or cytoplasmic acetylation-mimicking mutant TDP-43-2KQ-GFP or TDP-43-ΔNLS/2KQ-GFP, before anti-pTDP-43^(409/410)^ immunostaining, and confocal image segmentation analysis (Supplementary Fig. S10A). This revealed that low, medium, and high levels of over-expression produce a similar proportion of phospho-TDP-43-positive assemblies (Supplementary Fig. S10B), and that overall, the assemblies formed at each expression level were of similar size (Supplementary Fig. S10C). Importantly, we show that the acetylation-mimicking mutations and size of the assemblies, rather than over-expression level (increasing amounts of DNA transfected), was the important factor in determining phosphorylation state (Supplementary Fig. S10B,D).

We next investigated whether this sequestration alters the mobility dynamics of normal TDP-43-mCherry (Fig. 6i). As expected, FRAP analysis revealed that the incorporation of endogenous TDP-43 into acetylated cytoplasmic TDP-43 inclusions abolishes its mobility, in comparison to diffusely distributed nuclear TDP-43 (Fig. 6j). Either complete or partial bleaching of endogenous TDP-43 within nuclear or cytoplasmic inclusions both showed minimal fluorescence recovery, indicating almost no dynamic exchange with surrounding cytoplasmic proteins, or within the inclusion itself, respectively. Specifically, endogenous TDP-43 sequestered into large cytoplasmic TDP-43-ΔNLS/2KQ-GFP inclusions showed an AUC of 10.5 a.u. and m.f. of 7.2%, a maximal recovery of 12.6% (Fig. 6l) and time to reach half maximal (t_1/2_) of 17.2 s (Fig. 6m). In some cells, acetyl-mimicking TDP-43-GFP in the nucleus formed large inclusions that recruited endogenous TDP-43-mCherry, which also showed severe mobility impairment (Fig. 6k), demonstrated by AUC of 12.2 a.u., m.f. of 12.5, and significant decreases in maximal recovery of 14.7% (Fig. 6n), and t_1/2_ of 26.2 s (Fig. 6o). In contrast, endogenous TDP-43-mCherry incorporated more frequently into nuclear TDP-43-2KQ-GFP anisosomes and retained high mobility and rapid rate of recruitment (Fig. 6k), with AUC of 59.3 a.u., m.f. of 51.9%, maximal recovery of 37.0% (Fig. 6n) and t_1/2_ of 6.3 s (Fig. 6o). Dynamic properties of endogenous TDP-43 in anisosomes did not greatly differ overall from diffusely distributed endogenous TDP-43 in untransfected cells. This was likely due to the high concentration of nuclear acetylation-mimicking TDP-43 which formed anisosomes that readily recruited endogenous TDP-43. We note that the variability through time and decline following peak fluorescence recovery for acetylated TDP-43 anisosomes (Fig. 6k) is likely due to movement of these structures within the nucleus partially out of the FRAP region-of-interest. Together, these findings suggest that endogenous TDP-43 is specifically sequestered into a variety of pathological TDP-43 structures to significantly alter the solubility and mobility dynamics of endogenous TDP-43. Endogenous TDP-43 incorporated into large, phosphorylated nuclear or cytoplasmic acetyl-mimicking TDP-43 inclusions correlated strongly with decreased solubility, along with a dramatic loss of mobility, while endogenous TDP-43 within anisosomes comprised of acetyl-mimicking TDP-43 remained highly mobile.

## Discussion

It has remained unclear whether the accumulation of pathological or phase-separated TDP-43 proteins in ALS and FTD can directly divert normal TDP-43 away from its functional pool, to contribute to TDP-43 nuclear depletion and loss-of-function or exacerbate aggregation. Here, we found that introduction of aggregation-prone RNA-binding-ablated or acetylation-mimicking TDP-43 progressively decreases free nuclear TDP-43 abundance in a concentration-dependent manner. The most dramatic effect was observed with LLPS-mediated formation of numerous dynamic TDP-43 anisosomes by exogenous acetylated TDP-43 in the nucleus, which readily sequestered over half of total endogenous TDP-43 levels. Anisosome-dependent sequestration thereby caused dramatic mislocalisation and loss of TDP-43 availability, however endogenous TDP-43 confined to these shell-like assemblies remained soluble and highly mobile. On the other hand, fewer large inclusions formed by acetylation-mimicking TDP-43 in the nucleus or cytoplasm recruited a smaller proportion of endogenous TDP-43, but sequestration rendered it insoluble and immobile, denoting pathological transition. Endogenous wild-type TDP-43 also formed diverse highly dynamic biomolecular condensates via LLPS under various conditions. Indeed, disease-associated oxidative stress increased the formation of nuclear liquid droplet-like puncta, and induced formation of dynamic endogenous TDP-43 anisosomes, which notably were not formed by over-expressed wild-type TDP-43. These findings suggest mechanisms in which disease-associated stress or RNA-deficient, post-translationally modified TDP-43 assemblies potentiate dysfunction and aggregation by depleting free normal TDP-43 from the nucleus to potentially pathological structures. Importantly, normal TDP-43 becomes readily confined to liquid–liquid phase separated structures that may form dynamically in disease conditions. Our results emphasise the importance of concurrent changes occurring to normal nuclear TDP-43 prior to aggregation, and novel interactions with diverse pathological assemblies that may drive TDP-43-related neurodegeneration in ALS and FTD.

A loss of free nuclear TDP-43 in disease may be triggered by cellular stressors that induce nuclear-to-cytoplasmic redistribution or phase transitions of endogenous TDP-43 into condensed assemblies. Phase-separated TDP-43 proteins comprising stress-induced liquid droplets, SGs, and anisosomes likely remain soluble and dynamic, however confinement within these reversible structures may alter physiological TDP-43-RNA or -protein interactions [25, 68]. Furthermore, chronic induction, abnormal assembly, or aberrant phase transitions of TDP-43 condensates may seed intranuclear or cytoplasmic aggregation, as observed in degenerating neurons [24, 25, 32, 33, 59, 70]. Critically, TDP-43 LLPS has largely been studied by over-expressing TDP-43 constructs with mutations, truncations, or optogenetic clustering tags that promote self-assembly.

In this study we sought to characterise LLPS-related structures formed by endogenous TDP-43, to more closely study the physiological context. We consistently observed distinct liquid droplet-like ‘puncta’ under physiological conditions, but found that arsenite-mediated oxidative stress increased their formation, and also induced cytoplasmic G3BP1-positive TDP-43-positive SGs, aligning with previous studies of exogenous TDP-43 LLPS [31–34, 37, 71]. Strikingly, we found that oxidative stress increased LLPS-mediated assembly of rare endogenous TDP-43 anisosomes in live cells, the formation of which has been primarily characterised for over-expressed RNA-binding-ablated or acetylation-mimicking TDP-43 variants [25]. Interestingly, similar spherical shell-like structures have been shown to be formed by poly(A) RNA in spinal muscular atrophy models [72]. In previous studies, endogenous TDP-43 anisosome formation has only been demonstrated in post-mortem rat brains stressed with a combined treatment of histone deacetylase and proteasome inhibitors [25]. Notably, we found that arsenite-mediated oxidative stress did not cause over-expressed WT TDP-43 to form anisosomes in this experimental paradigm. We speculate that with over-expression in our cell lines, excessive WT TDP-43 in the nucleus may favour formation of other assemblies or disease-reminiscent inclusions, rather than dynamic anisosomes. However, there may be cell-type-specific differences in over-expression and modulators of TDP-43 LLPS-mediated assembly between these immortalised cell lines and other neuronal models. For example, previous work has shown that it is indeed possible for over-expressed WT TDP-43 to form anisosomes in neuron-like SH-SY5Y cells and iPSC-derived motor neurons, following synergistic histone deacetylase and proteasome inhibition [25]. Therefore, further analysis of endogenous-tagged TDP-43 LLPS in human neurons is warranted. Other work has reported arsenite stress-induced co-condensation of exogenous WT TDP-43-HA with over-expressed RFP-Hsp70 or RFP-Hsc70 proteins into numerous small nuclear puncta [37], however shell-like anisosomes were not observed under these conditions. These data suggest that diverse cellular stressors can stimulate formation of anisosomes comprising normal endogenous RNA-binding-competent WT TDP-43. For endogenous TDP-43 anisosome formation as seen in our study, oxidative stress may trigger cellular responses altering substrate RNA availability, and thereby TDP-43-RNA interactions, leading to changes in TDP-43 stability that trigger LLPS-mediated assembly into dynamic RNA-deficient anisosomes [73, 74]. It remains unclear what other cellular stressors trigger endogenous TDP-43 anisosome formation, and whether confinement of RNA-binding-competent TDP-43 into anisosomes impacts its RNA-regulatory capacity and thereby constitutes loss of functional nuclear TDP-43.

Accumulation of pathological TDP-43 itself likely drives nuclear TDP-43 depletion by decreasing endogenous TDP-43 expression via auto-regulation [5, 30, 38], or inducing mislocalisation by disrupting processes such as nucleocytoplasmic transport [75]. However, potentially independent of these mechanisms, we found striking changes in endogenous TDP-43 distribution within cells over-expressing exogenous TDP-43-GFP in a concentration-dependent manner. Notably, endogenous TDP-43-mCherry was sequestered into assemblies formed by pathological TDP-43-GFP variants, becoming diverted from physiological nuclear localisation and function, suggesting that a self-perpetuating cycle of pathological conversion may drive TDP-43 dysfunction in disease. Indeed, single-cell analysis revealed that nuclear RNA-binding-ablated 4FL or acetylation-mimicking 2KQ variants in particular showed exaggerated effects, with the formation of dynamic shell-like anisosome structures sequestering the largest proportion of endogenous TDP-43 for a given amount of mutant TDP-43-GFP pathology. In the cytoplasm, we similarly found that the addition of pathology-mimicking 4FL or 2KQ mutations increased inclusion and puncta formation over and above the ∆NLS-only variant, however with only occasional recruitment of endogenous TDP-43, indicating compartment-specific tendencies for sequestration. While over-expression of WT, or other TDP-43-GFP variants did not significantly alter total endogenous TDP-43 levels in this cell system (likely due to technical considerations including low and variable transfection efficiency in some variants), we showed that the dramatic redistribution into mutant inclusions, anisosomes, and/or puncta completely accounted for the loss of diffuse nuclear endogenous TDP-43. Our study therefore highlights that concentration-dependent sequestration of normal TDP-43 to pathological structures over time, which is exacerbated by acetylation and RNA-binding impairment, may drive nuclear depletion of TDP-43 in ALS and FTD. Such sequestration, or ‘co-aggregation’, of over-expressed WT TDP-43 has been demonstrated upon introduction of disordered or mislocalised TDP-43 proteins, with scarce evidence of endogenous human TDP-43 being sequestered in cell lines [33, 61, 64, 76]. Previous literature and our current findings indicate that RNA molecules may not be required for TDP-43 inclusion formation, as many forms of TDP-43 with truncation or mutation of the RRMs are highly aggregation-prone [21, 25, 29, 32, 39–43], and we have shown here that RNA-binding-deficient TDP-43 mutants readily form inclusions and recruit endogenous nuclear TDP-43. Previous work has shown that both acetylation-mimicking and RNA-binding site TDP-43 mutants bind substantially less RNA and have dramatically decreased RNA splicing activity [21]. Contrastingly, recent studies have shown that RNA-binding deficient TDP-35-4FL C-terminal fragments do not sequester endogenous TDP-43, and instead that TDP-43-RNA interactions may promote sequestration of normal RNA-binding proteins to aggregation-prone forms of TDP-43 [64, 77]. However, these results are not directly comparable to our study as mutant TDP-43 C-terminal fragments likely exhibit different aggregation properties compared to our full-length TDP-43 mutants [64, 77]. Therefore, the role of RNA-binding capacity in aggregation and endogenous TDP-43 sequestration requires further investigation.

Since we found that pathological acetylated or RNA-binding-ablated TDP-43 assemblies could recruit normal nuclear TDP-43 in live cells, we also sought to determine whether this sequestration caused transition to a pathological state. Acetylation-mimicking TDP-43 anisosomes that readily recruited a majority of endogenous TDP-43 were predominantly phospho-TDP-43-negative (Fig. 6a), and sequestered proteins remains highly mobile (Fig. 6i, k, n, o). Large nuclear or cytoplasmic inclusions formed by acetylation-mimicking TDP-43 that contained endogenous TDP-43 were often phosphorylated, which has been previously linked to acetylation [22], and is reminiscent of end-stage disease pathology [78]. Furthermore, expression of acetylation-mimicking TDP-43 increased levels of insoluble endogenous TDP-43 and phosphorylated TDP-43, concurrent with a slight decrease in soluble endogenous TDP-43. We demonstrated that phosphorylation state and sequestration of endogenous TDP-43 to acetylated TDP-43 assemblies was indeed determined by aggregation-prone mutant characteristics and the size of inclusions formed, and not over-expression level (Supplementary Fig. S8). Importantly, sequestration of normal TDP-43 to large pathology-reminiscent nuclear or cytoplasmic inclusions led to complete loss of protein mobility, likely indicating irreversible pathological transitions and loss-of-function, despite no change in total endogenous TDP-43 levels. Previous work has demonstrated co-precipitation of endogenous TDP-43 with disease-associated TDP-43 C-terminal fragments [64], with others showing that over-expression of cytoplasmic ∆NLS mutant TDP-43 increases insoluble levels of endogenous TDP-43 [61]. We now further report sequestration of endogenous normal nuclear TDP-43 to various RNA-deficient inclusions, anisosomes, or puncta in either the nucleus or cytoplasm of live cells, and show that this recruitment alters dynamic properties of endogenous TDP-43 to conform to exogenous mutant TDP-43 proteins that rapidly form these structures. Overall, these findings indicate that TDP-43 acetylation increases both nuclear and cytoplasmic aggregation propensity, and also promotes sequestration and pathological transitions of normal TDP-43, to potentiate disease-associated TDP-43 nuclear depletion, dysfunction, and pathology formation in ALS and FTD.

While more dramatic sequestration was caused by dynamic acetylated TDP-43 anisosomes, the pathophysiological implications and overall relevance to human disease pathology are yet to be established. Normal TDP-43 within anisosomes retained liquid-like properties and dynamic exchange with the free nuclear pool of TDP-43, corresponding with high mobility of over-expressed TDP-43^2KQ^-mClover anisosomes previously characterised [25]. However, it is unknown whether normal TDP-43 without prior post-translational modification or RNA-binding impairment recruited to anisosomes can interact with RNA, and has been difficult to determine through post-mortem studies whether acetylated TDP-43 anisosomes inhibit or seed TDP-43 aggregation, or whether anisosomes indeed form in human ALS and FTD [25]. In either case, we have demonstrated that sequestration—even to dynamic assemblies transiently formed under disease-associated stress or RNA-binding impairment in the nucleus—could be an early disease mechanism of TDP-43 dysfunction before extensive aggregation, by critically disrupting normal nuclear TDP-43 localisation, availability, and physiological interactions. Importantly, this process could drive loss-of-function mechanisms of neurodegeneration, independently of changes to overall levels of normal nuclear TDP-43.

It is important to still note sequestration to cytoplasmic acetylated TDP-43 inclusions as a key factor in potentiating dysfunction and aggregation of endogenous TDP-43. Although it did not occur as readily as that of nuclear assemblies, cytoplasmic sequestration caused insolubility and loss of protein mobility for endogenous normal nuclear TDP-43, still exerting a considerable effect on cell-wide TDP-43 solubility and dynamics. The difference in sequestration tendency for cytoplasmic assemblies may simply be a result of the relative exposure of endogenous TDP-43 proteins over time, through only a small amount of nucleocytoplasmic shuttling. In fact, this may be even more reminiscent of ALS and FTD, diseases of ageing which involve decades of slow pathology accumulation throughout the brain and spinal cord [79, 80]. Combination with other disease-associated cellular stressors that drive TDP-43 to the cytoplasm [7, 81], could therefore exacerbate this sequestration and be a strong driver of pathology formation in humans.

Due to the constraints of our models and experimental paradigms, we were unable to fully evaluate the functional consequences for the sequestration of endogenous normal TDP-43 to both large pathology-reminiscent inclusions, and the dynamic shell-like anisosome assemblies, which should be of great interest to the field. Outstanding questions in the field include the relative contributions and mechanisms of TDP-43 loss-of-function compared to concurrent toxic aggregation, and establishing this in disease-relevant models. Important future directions could apply functional read-outs for RNA-binding activity when TDP-43 is confined to different pathological assemblies, in particular anisosomes which remain incompletely characterised. Ultimately this is difficult to discern in this study due to potential interference of exogenous TDP-43 variants, that necessarily be introduced to form such assemblies, with endogenous TDP-43 RNA substrates. Future experiments that employ the CRISPR-edited cell lines developed in this study could be used to conduct single-cell analyses to interrogate changes in cells with different TDP-43 assemblies. This could reveal disease-relevant mechanisms of RNA dyshomeostasis that further place endogenous TDP-43 sequestration in a pathological context, for example with increasing evidence for the role of TDP-43 in cryptic exon splicing and downstream toxicity or dysfunction related to cryptic exon inclusion [82].

Studying endogenous TDP-43 protein over time is advantageous for elucidating disease-relevant mechanisms driving depletion and aggregation of the native TDP-43 protein, which becomes pathological in almost all cases of ALS and half of FTD [1]. Our CRISPR-tagging approach allows for live-cell visualisation of endogenous TDP-43 in real-time, overcoming limitations of over-expression studies, end-point analyses and traditional staining techniques, to reveal assembly and composition of complex TDP-43 structures. CRISPR/Cas9-mediated fluorescent tagging of endogenous *TARDBP* has been reported in HEK293T cells for live-cell imaging [77], in human neuroblastoma cells for FRAP [33] or in iPSCs for real-time quantification of nuclear endogenous TDP-43 [33, 77, 83]. Our unique CRISPR method, whereby one multiplexed editing reaction mediated insertion of two different fluorescent tags across different *TARDBP* alleles, demonstrates potential for differential fluorescence tagging of multiple distinct endogenous proteins for concurrent intracellular analysis. Recently, CRISPR-tagged endogenous myc-TDP-43 zebrafish models have been developed [84], supporting feasibility of fluorescently-tagged endogenous TDP-43 in vivo models. To our knowledge, this is the first endogenous-tagging application of mAvicFP1 [67], which favourably exhibits high fluorescence intensity and photostability, and is amenable to single-molecule super-resolution microscopy [85]. Future directions in the use of such *TARDBP-mAvicFP1/-mCherry* cell models include CRISPR-screening applications, live-cell single-molecule imaging or differential analysis of specific mutations of endogenous TDP-43. Finally, as we characterised dynamic TDP-43 structures in live cells (e.g. anisosomes) that were not formed by over-expressed TDP-43, it will be interesting to see whether additional novel dynamic TDP-43 assemblies could be discovered with high-resolution real-time imaging.

## Conclusions

To interrogate molecular mechanisms by which normal nuclear TDP-43 protein becomes mislocalised and aggregated in disease, this study sought to investigate whether disease-associated stressors or pathology-mimicking TDP-43 variants alter endogenous TDP-43 abundance, localisation, self-assembly, aggregation, and mobility dynamics over time. Overall, our results indicate that complex dynamic processes control self-assembly and recruitment of normal TDP-43 to a diverse array of both nuclear and cytoplasmic structures related to disease. In particular, RNA-binding-ablated or acetylation-mimicking TDP-43 modifications enhanced aggregation propensity, leading to more dramatic sequestration and depletion of diffuse nuclear endogenous TDP-43 for a given pathology load. Sequestration into either disease-reminiscent inclusions or dynamic phase-separated structures directly modified solubility and mobility dynamics of endogenous TDP-43 proteins in real-time, revealing important disease mechanisms that may perpetuate TDP-43 dysfunction and TDP-43 aggregation in ALS and FTD.

## Materials and methods

Chemicals were sourced from Sigma unless otherwise stated.

### CRISPR/Cas9-mediated fluorescent protein knock-in to endogenous *TARDBP* genomic locus

CRISPR/Cas9 gene-editing was used to generate endogenous TDP-43-tagged HEK293T cell lines with the insertion of fluorescent tags, mAvicFP1 and mCherry, at the *TARDBP* C-terminus. The coding sequences for mAvicFP1, a monomeric, bright and photostable green fluorescent protein [67], or the red fluorescent protein mCherry, were integrated immediately upstream of the exon 6 STOP codon, following a dual DYK*-* and myc-tag linker sequence (Fig. 1a-d). Gene insertion was mediated by homologous recombination of the PCR amplicon DNA donor template, which contained the fluorescent tag insertion sequence flanked by homology arms (HAs) that specifically anneal to the C-terminal Cas9-cleaved *TARDBP *sequence. 

#### Single guide RNA and donor template primer design

sgRNA oligonucleotides and double-stranded DNA (dsDNA) donor templates for mAvicFP1/mCherry insertion were designed using the program *Benchling* and synthesised by *GenScript*. The CRISPR RNA (crRNA) target sequence (5ʹ-GCTGGGGAATGTAGACAGTG-3 ʹ) was designed to anneal to the *TARDBP* C-terminus and direct Cas9-mediated DSB 3 base-pairs (bp) upstream of the PAM site. Donor templates featured the insertion sequences of *mAvicFP1* or *mCherry*, preceded by a mutation of the PAM-site (GGG > GGA) to prevent future Cas9-DSBs after successful gene-editing, and followed by *FLAG* and *myc* 'linkers' to separate fluorescent protein tags from TDP-43 to ensure normal folding. Insertion sequences were flanked by 50 bp recessed or 56 bp proximal HAs that were complementary to the endogenous *TARDBP* C-terminal sequence, to specifically direct insertion 6 bp upstream of the DSB. The donor templates were designed such that *FLAG* and *myc* sequences not only serve as a protein linker, but can be detected with FLAG or myc antibodies for downstream analysis of the edited protein.

#### PCR amplification of donor DNA templates for homology-directed repair

Donor templates for homology-directed repair were produced as linear DNA fragments from PCR amplification, which has been shown to produce greater editing efficiency compared to circular DNA constructs, and allow for higher DNA template concentrations to be applied [86]. Donor template PCR products were purified using the QIAquick PCR Purification kit (QIAGEN, 28104), eluted in minimum volumes of 15 µL to maximise DNA concentration. The expected DNA band size of these amplicons (mAvicFP1 877 bp, mCherry 871 bp) was confirmed by analysing donor template PCR products on a 1% (w/v) agarose gel at 85 V for 1 h.

#### Ribonucleoprotein (RNP) complex assembly and electroporation delivery

Functional sgRNAs were formed by hybridisation of crRNA and trans-activating CRISPR RNA (tracrRNA) (5ʹ-AGCAUAGCAAGUUAAAAUAAGGCUAGUCCGUUAUCAACUUGAAAAAGUGGCACCGAGUCGGUGCUUU-3ʹ), whereby lyophilised crRNA and tracrRNA oligonucleotides were each reconstituted to 100 µM in sterile Tris–EDTA, and then combined in equimolar ratio to a final concentration of 40 µM, diluted with duplex buffer (100 mM potassium acetate, 30 mM HEPES, pH 7.5). This duplex was heated at 95 °C for 5 min and cooled at room temperature for 2 h, then diluted to 10 µM in Tris–EDTA. RNP complexes were created by mixing 1.625 µL cr:tracrRNA duplex (10 µM) with 2.6 µL purified NLS-Cas9-NLS protein (6.25 µM) gently by pipetting, and incubating for 10–60 min.

300,000 HEK293T cells were aliquoted for each electroporation condition (DNA-free negative control, *mAvicFP1/mCherry* PCR-amplicon donor templates and pEGFP-N1 positive control). The RNP complex and donor DNA was delivered to the cells by electroporation using the Neon Transfection System 100 µL kit (ThermoFisher, MPK10025) with Electrolytic Buffer-E2. Cells were resuspended in Buffer-R and transferred to the corresponding RNP complex tubes. 2.6 µg of each dsDNA donor template was used. HEK293T cells/RNP complexes/donor templates were mixed and aspirated into a 100 µL Neon tip and electroporated with 1200 V, 2 pulses, 30 ms pulse width. The pipette was immediately removed from the device and cells transferred to a 12-well plate prepared with 1 mL warm media, and incubated for 3 days.

### Fluorescence-activated cell sorting (FACS) isolation of successfully edited cells and selection of monoclonal cell lines

CRISPR/Cas9-edited cells were obtained using the BD-Influx Cell Sorter with 488 nm and 561 nm lasers for excitation of mAvicFP1 and mCherry, respectively. Quandrant gating defined cell populations that were mAvicFP1-positive, mCherry-positive, and ‘dual-tagged’ mAvicFP1-positive/mCherry-positive, which were seeded as single cells into 96-well plates. Single cells were allowed to grow into monoclonal colonies and scanned for mAvicFP1 and mCherry fluorescence intensity using high-content microscopy, which distinguished low-, medium-, and high-intensity TDP-43-tagged cell lines. High-intensity cell lines *TARDBP-mAvicFP1(H8)*, *TARDBP-mCherry(B8)*, and *TARDBP-mAv/-mCh(C4)* were utilised for subsequent experiments throughout this study.

### Sanger sequencing

Genomic DNA was extracted from approximately 1,000,000 cells of the CRISPR-edited *TARDBP-mAvicFP1(H8)*, *TARDBP-mCherry(B8)*, or *TARDBP-mAv/-mCh(C4)* HEK293T lines with a Bioline Isolate II Genomic DNA Kit (#BIO-52066). For each cell line, a ~ 900 bp ‘genotyping zone’, comprising the C-terminal *TARDBP* fluorescent tag insertion site was amplified by PCR, with specific primers annealing to the upstream *TARDBP* exon 6 sequence and *mAvicFP1* or *mCherry* sequence. PCR products were analysed by DNA electrophoresis on a 1% agarose gel, before gel purification of the expected genotyping template bands, using a Bioline Isolate II Gel and PCR Kit (#BIO52059). Forward or reverse sequencing primers were each combined with 75 ng of gel-purified genomic DNA genotyping sequences, and supplied to Australian Genome Research Facility for Sanger sequencing.

### Cell culture and transfection

Human Embryonic Kidney (HEK293T/17; ATCC^®^-CRL-11268) cells were maintained in Dulbecco’s Modified Eagle Medium/Ham F12 media (DMEM/F12) with 10% (v/v) foetal-calf-serum (Gibco). Cells were incubated at 37 °C, 5% CO_2_ and passaged at 80% confluency, and tested regularly for mycoplasma contamination. Cells were transfected using Lipofectamine 2000 (Invitrogen) and plasmid DNA according to the manufacturer’s instructions. Plasmids included human pLenti-C-mGFP, pLenti-TDP-43^WT^-C-mGFP, and pLenti-TDP-43^4FL^-C-mGFP, pLenti-TDP-43^2KQ^-C-mGFP, pLenti-TDP-43^∆NLS^-C-mGFP, pLenti-TDP-43^∆NLS/4FL^-C-mGFP, or pLenti-TDP-43^∆NLS/2KQ^-C-mGFP. Cells were seeded onto coverslips in 24-well plates at a density of 25,000 cells/well and incubated for 24 h. For transfections, 0.5 µg DNA and 0.75 µL Lipofectamine 2000 were used in a total of 100 µL OptiMEM (Gibco) per well. Cells were incubated for 48 h to allow protein expression from the plasmid DNA before analysis by immunoblot, live-cell imaging, or immunocytochemistry.

### Plasmids

C-terminal GFP-tagged wild-type hTDP-43 construct was generated by Origene, from a pLenti-C-mGFP backbone [87]. Mutant hTDP-43 constructs were then generated through modification of pLenti-hTDP-43^WT^-mGFP by GenScript: pLenti-hTDP-43^4FL^-mGFP (F147L, F149L, F229L, F231L), pLenti-hTDP-43^2KQ^-mGFP (K145Q, K192Q), pLenti-hTDP-43^ΔNLS^-mGFP (K82A, R83A, K84A), pLenti-hTDP-43^ΔNLS/4FL^-mGFP (K82A, R83A, K84A, F147L, F149L, F229L, F231L), pLenti-hTDP-43^ΔNLS/2KQ^-mGFP (K82A, R83A, K84A, K145Q, K192Q). All constructs generated and used in this work were verified by Sanger sequencing.

### Immunoblotting

At experiment endpoints, cells were harvested and washed in ice-cold PBS, before soluble and insoluble protein fractions were extracted by sequential RIPA-buffered (50 mM Tris [pH 7.5], 150 mM NaCl, 1% [v/v] NP-40, 0.5% [w/v] sodium deoxycholate, 0.1% [w/v] SDS, 1 mM EDTA, 1 mM PMSF, 1 × protease/phosphatase inhibitor cocktail) and urea-buffered protein lysis, Precellys homogenisation and ultracentrifugation. RIPA-soluble protein extracts were quantified using a BCA Protein Quantification Kit (Pierce) and plates were analysed on a ClarioStar Optima plate reader (BMG Lab Technologies). 50 µg of RIPA-soluble protein and an equivalent volume of the corresponding urea-soluble fraction were analyzed by 12% SDS-PAGE, run for 150 V for 60 min and then transferred to a nitrocellulose membrane (Li-Cor) at 100 V for 90 min. Total protein was quantified with Revert 700 total protein stain (#912-11011, Li-Cor) according to the manufacturer’s instructions, before membranes were blocked in 5% (w/v) BSA Tris-buffered saline (0.5 M Tris-base, 1.5 M NaCl) with 0.05% (v/v) Tween-20 (TBS-T). Blots were immunolabelled overnight at 4ºC with primary antibodies, including a rabbit anti-TDP-43 polyclonal antibody (PAb) (1:2000, #10782-2-AP, ProteinTech), mouse anti-myc monoclonal antibody (MAb) (1:500, #2276S [Clone 9B11], Cell Signaling Technologies), rat anti-phospho TDP-43 (ser409/410) MAb (1:1000, #829901 [Clone 1D3], BioLegend [Previously Covance #SIG-39852]), mouse anti-FLAG MAb (1:1000, #F1804 [Clone M2], Sigma), and rabbit anti-mCherry PAb (1:1000, #ab167453, Abcam). Secondary antibodies included goat anti-mouse IRDye-800, goat anti-rabbit IRDye-800, goat anti-rat IRDye800, goat anti-mouse IRDye-680 or goat anti-rabbit IRDye-680 conjugated secondary PAbs (Li-Cor). Signals were detected by imaging with the Li-Cor Odyssey Clx Infrared Imaging System, and quantified using Li-Cor Image Studio version 3.1.

### Live-cell imaging

In all live-cell imaging experiments, cell nuclei were stained with Hoechst (1:10,000, #33342, Thermo Fisher) for 5 min and washed with fresh media prior to imaging. For time-course imaging of acetylation-mimicking TDP-43 anisosome formation, mobility, fusion and disassembly, *TARDBP-mCherry(B8)* cells seeded at 30,000 cells/well in 24-well plates were transfected to over-express mutant TDP-43-2KQ-GFP mutants for 48 h, and transferred to the CELENA X live-cell imaging microscope (Logos Biosystems), within an insulated chamber at 37 °C and 5% CO_2_. Brightfield, mCherry and GFP channels were imaged at 20X magnification for 20 ROIs, every 10 min for 2 h, with laser auto-focus in every ROI. For 1 h continuous imaging of *TARDBP-mAvicFP1(H8)* cells treated with or without sodium arsenite, 20,000 cells/dish seeded in Cellvis glass-bottom 20 mm dishes (#D29-20-1.5-N) were transferred to the Zeiss LSM 710 confocal microscope and identified with a Plan-Apochromat 63 × oil objective (1.4 NA, 190 µm WD, 0.19 µm/pixel), blue laser diode for DAPI (405 nm), argon laser for AlexaFluor488 (488 nm), and 561 nm laser for AlexaFluor555 excitation. During imaging, cells were contained within an insulated chamber at 37 °C and 5% CO_2_. The GFP channel was imaged every 1 min for 1 h, at 63X magnification, with Z-stacks of 6–8 slices separated by the recommended optimal distance. Image processing was completed with FIJI, including maximum Z-projections.

### Fluorescence recovery after photobleaching (FRAP)

For FRAP imaging of *TARDBP-mAvicFP1(H8)* cells treated with or without sodium arsenite or *TARDBP-mCherry(B8)* cells over-expressing TDP-43-GFP mutants, cells seeded at 20,000 cells/dish in Cellvis glass-bottom 20 mm dishes (#D29-20-1.5-N) were transferred to the Zeiss LSM 710 confocal microscope, were cells were contained within an insulated chamber at 37 °C and 5% CO_2_. Cells were identified using a Plan-Apochromat 63 × 1.4NA oil objective on the Zeiss LSM 710 confocal microscope, and Zeiss ZEN software was used for data acquisition. FRAP acquisition was performed on cells within a field of view including adjacent cells of similar intensity (to control for photo-bleaching). A baseline of 3 images was acquired (using 4% argon 488 or 561 nm laser intensity) prior to bleaching. Approximately 2.5 μm regions of interest on the cells were bleached for ~ 3 s using 100% argon 488 or 561 nm laser intensity, to completely deplete fluorescence intensity of endogenous TDP-43 within structures of interest. The image scan speed was set at 10. Data presented are representative of three or more biologically independent repeats, striving for at least 5 individual cells per replicate. Acquired data normalised using the web-based resource tool, EasyFRAP [88], correcting to both the background intensity level (a region outside cell boundaries) and the surrounding diffuse protein pool (within nucleus or cytoplasm, depending on subcellular location of the structure of interest). Mobile fractions (m.f.) and recovery time constants (τ) were calculated using Zen software from regions of interest of curve-fitted data.

### Immunofluorescence staining

Cells on glass coverslips were fixed with 4% paraformaldehyde in phosphate-buffered saline (PBS) for 10 min at room temperature, before blocking and permeabilisation with 3% BSA in PBS-T (+ 0.1% Triton-X100) for 1 h at room temperature. Primary antibodies were diluted in blocking and permeabilisation buffer, including a rabbit anti-TDP-43 PAb (1:1000, #10782-2-AP, ProteinTech), rabbit anti-phospho TDP-43 (ser403/404) PAb (1:1000, CAC-TIP-PTD-P05, CosmoBio), rat anti-phospho TDP-43 (ser409/410) MAb (1:250, #829901 [Clone 1D3], BioLegend [Previously Covance #SIG-39852]), rabbit anti-G3BP1 PAb [1:500, #13057-2-AP, ProteinTech], mouse anti-FLAG MAb (1:1000, #F1804 [Clone M2], Sigma), and applied overnight at room temperature. Secondary antibodies were similarly prepared, including goat anti-Rabbit H+L AlexaFluor647 (1:500, A-21245, Life Tech), and applied for 1.5 h at room temperature. Cells were counterstained with DAPI (1:1000, #62248, Thermo Fisher) for 10 min at room temperature, mounted onto microscope slides with ProLong Gold Antifade Mountant (#P36934, ThermoFisher), and allowed to cure overnight at room temperature before microscopy.

### Confocal microscopy

Confocal images were captured using either the Zeiss Laser Scanning Microscope (LSM) 510 META or Zeiss LSM 710. Both are equipped with a 63 × oil objective (1.4 NA, 190 µm WD, 0.19 µm/pixel), blue laser diode for DAPI (405 nm), argon laser for AlexaFluor488 (488 nm), 561 nm laser for AlexaFluor555, and HeNe 633 nm laser for AlexaFluor647 excitation. Images were acquired using Zen software and all acquisition parameters were applied consistently for all samples in an experimental set. Image processing and intensity profile analyses were completed with FIJI. Automated and unbiased single-cell segmentation analyses were conducted using CellProfiler version 4.2.1 [89] using custom pipelines.

### Statistical analysis

Data were represented as mean ± standard error about the mean (SEM). GraphPad Prism 9 was used for statistical analysis and preparation of graphs, with *P* values < 0.05 deemed statistically significant. Live-cell imaging of nuclear TDP-43-mCherry expression over time included *n* = 3 independent repeats, recording 12 ROIs at 20 × magnification for each experimental condition. FRAP experiments also included *n* = 3 independent repeats, with at least 5 cells per replicate for each of the distinct TDP-43 assemblies studied. All immunoblot experiments included *n* = 3 independent repeats. Individual data points for each independent replicate are shown overlaid with a line at the mean. Statistically significant differences between the means were determined using a two-tailed paired *t*-test (where indicated in figure legends) for comparisons between only two groups, or a one-way ANOVA with a Tukey’s post hoc test for multiple comparisons in experiments with several groups.

### Supplementary Information

Below is the link to the electronic supplementary material.

Supplementary file1 (XLSX 503 KB)

Supplementary file2 (XLSX 79 KB)

Supplementary file3 (XLSX 250 KB)

Supplementary file4 (XLSX 76734 KB)

Supplementary file5 (MP4 16010 KB)

Supplementary file6 (MP4 9739 KB)

Supplementary file7 (MP4 23411 KB)

Supplementary file8 (PDF 9613 KB)

## Data Availability

Supporting data are available as electronic supplementary material.

## References

[1] Ling SC, Polymenidou M, Cleveland DW (2013). Converging mechanisms in ALS and FTD: disrupted RNA and protein homeostasis. Neuron.

[2] Neumann M, Sampathu DM, Kwong LK, Truax AC, Micsenyi MC, Chou TT (2006). Ubiquitinated TDP-43 in frontotemporal lobar degeneration and amyotrophic lateral sclerosis. Science.

[3] Polymenidou M, Lagier-Tourenne C, Hutt KR, Huelga SC, Moran J, Liang TY (2011). Long pre-mRNA depletion and RNA missplicing contribute to neuronal vulnerability from loss of TDP-43. Nat Neurosci.

[4] Tollervey JR, Curk T, Rogelj B, Briese M, Cereda M, Kayikci M (2011). Characterizing the RNA targets and position-dependent splicing regulation by TDP-43. Nat Neurosci.

[5] Ayala YM, De Conti L, Avendaño-Vázquez SE, Dhir A, Romano M, D'Ambrogio A (2011). TDP-43 regulates its mRNA levels through a negative feedback loop. Embo J.

[6] Buratti E, Baralle FE (2010). The multiple roles of TDP-43 in pre-mRNA processing and gene expression regulation. RNA Biol.

[7] Keating SS, San Gil R, Swanson MEV, Scotter EL, Walker AK (2022). TDP-43 pathology: from noxious assembly to therapeutic removal. Progr Neurobiol.

[8] Barmada SJ, Skibinski G, Korb E, Rao EJ, Wu JY, Finkbeiner S (2010). Cytoplasmic mislocalization of TDP-43 is toxic to neurons and enhanced by a mutation associated with familial amyotrophic lateral sclerosis. J Neurosci.

[9] Dyer MS, Reale LA, Lewis KE, Walker AK, Dickson TC, Woodhouse A (2021). Mislocalisation of TDP-43 to the cytoplasm causes cortical hyperexcitability and reduced excitatory neurotransmission in the motor cortex. J Neurochem.

[10] Vatsavayai SC, Yoon SJ, Gardner RC, Gendron TF, Vargas JNS, Trujillo A (2016). Timing and significance of pathological features in C9orf72 expansion-associated frontotemporal dementia. Brain.

[11] Nana AL, Sidhu M, Gaus SE, Hwang J-HL, Li L, Park Y (2019). Neurons selectively targeted in frontotemporal dementia reveal early stage TDP-43 pathobiology. Acta Neuropathol.

[12] Braak H, Del Tredici K (2018). Anterior cingulate cortex TDP-43 pathology in sporadic amyotrophic lateral sclerosis. J Neuropathol Exp Neurol.

[13] Igaz LM, Kwong LK, Lee EB, Chen-Plotkin A, Swanson E, Unger T (2011). Dysregulation of the ALS-associated gene TDP-43 leads to neuronal death and degeneration in mice. J Clin Investig.

[14] Liu EY, Russ J, Cali CP, Phan JM, Amlie-Wolf A, Lee EB (2019). Loss of nuclear TDP-43 Is associated with decondensation of LINE retrotransposons. Cell Rep.

[15] Wu LS, Cheng WC, Chen CY, Wu MC, Wang YC, Tseng YH (2019). Transcriptomopathies of pre- and post-symptomatic frontotemporal dementia-like mice with TDP-43 depletion in forebrain neurons. Acta Neuropathol Commun.

[16] Jeong YH, Ling JP, Lin SZ, Donde AN, Braunstein KE, Majounie E (2017). Tdp-43 cryptic exons are highly variable between cell types. Mol Neurodegener.

[17] Walker AK, Spiller KJ, Ge G, Zheng A, Xu Y, Zhou M (2015). Functional recovery in new mouse models of ALS/FTLD after clearance of pathological cytoplasmic TDP-43. Acta Neuropathol.

[18] Eck RJ, Kraemer BC, Liachko NF (2021). Regulation of TDP-43 phosphorylation in aging and disease. Geroscience.

[19] Neumann M, Kwong LK, Lee EB, Kremmer E, Flatley A, Xu Y (2009). Phosphorylation of S409/410 of TDP-43 is a consistent feature in all sporadic and familial forms of TDP-43 proteinopathies. Acta Neuropathol.

[20] Hasegawa M, Arai T, Nonaka T, Kametani F, Yoshida M, Hashizume Y (2008). Phosphorylated TDP-43 in frontotemporal lobar degeneration and amyotrophic lateral sclerosis. Ann Neurol.

[21] Cohen TJ, Hwang AW, Restrepo CR, Yuan CX, Trojanowski JQ, Lee VM (2015). An acetylation switch controls TDP-43 function and aggregation propensity. Nat Commun.

[22] Wang P, Wander CM, Yuan C-X, Bereman MS, Cohen TJ (2017). Acetylation-induced TDP-43 pathology is suppressed by an HSF1-dependent chaperone program. Nat Commun.

[23] Kametani F, Obi T, Shishido T, Akatsu H, Murayama S, Saito Y (2016). Mass spectrometric analysis of accumulated TDP-43 in amyotrophic lateral sclerosis brains. Sci Rep.

[24] Chen Y, Cohen TJ (2019). Aggregation of the nucleic acid–binding protein TDP-43 occurs via distinct routes that are coordinated with stress granule formation. J Biol Chem.

[25] Yu H, Lu S, Gasior K, Singh D, Vazquez-Sanchez S, Tapia O (2021). HSP70 chaperones RNA-free TDP-43 into anisotropic intranuclear liquid spherical shells. Science.

[26] Zacco E, Martin SR, Thorogate R, Pastore A (2018). The RNA-recognition motifs of TAR DNA-binding protein 43 may play a role in the aberrant self-assembly of the protein. Front Mol Neurosci.

[27] Flores BN, Li X, Malik AM, Martinez J, Beg AA, Barmada SJ (2019). An intramolecular salt bridge linking TDP43 RNA binding, protein stability, and TDP43-dependent neurodegeneration. Cell Rep.

[28] Pérez-Berlanga M, Wiersma VI, Zbinden A, De Vos L, Wagner U, Foglieni C et al (2022) TDP-43 oligomerization and RNA binding are codependent but their loss elicits distinct pathologies. bioRxiv: 2022.05.23.493029

[29] Chen HJ, Topp SD, Hui HS, Zacco E, Katarya M, McLoughlin C (2019). RRM adjacent TARDBP mutations disrupt RNA binding and enhance TDP-43 proteinopathy. Brain.

[30] Hallegger M, Chakrabarti AM, Lee FCY, Lee BL, Amalietti AG, Odeh HM (2021). TDP-43 condensation properties specify its RNA-binding and regulatory repertoire. Cell.

[31] Maharana S, Wang J, Papadopoulos DK, Richter D, Pozniakovsky A, Poser I (2018). RNA buffers the phase separation behavior of prion-like RNA binding proteins. Science.

[32] Mann JR, Gleixner AM, Mauna JC, Gomes E, DeChellis-Marks MR, Needham PG (2019). RNA binding antagonizes neurotoxic phase transitions of TDP-43. Neuron.

[33] Gasset-Rosa F, Lu S, Yu H, Chen C, Ze M, Guo L (2019). Cytoplasmic TDP-43 de-mixing independent of stress granules drives inhibition of nuclear import, loss of nuclear TDP-43, and cell death. Neuron.

[34] McGurk L, Gomes E, Guo L, Mojsilovic-Petrovic J, Tran V, Kalb RG (2018). Poly(ADP-Ribose) prevents pathological phase separation of TDP-43 by promoting liquid demixing and stress granule localization. Mol Cell.

[35] Molliex A, Temirov J, Lee J, Coughlin M, Kanagaraj Anderson P, Kim Hong J (2015). Phase separation by low complexity domains promotes stress granule assembly and drives pathological fibrillization. Cell.

[36] Li HR, Chiang WC, Chou PC, Wang WJ, Huang JR (2018). TAR DNA-binding protein 43 (TDP-43) liquid–liquid phase separation is mediated by just a few aromatic residues. J Biol Chem.

[37] Gu J, Wang C, Hu R, Li Y, Zhang S, Sun Y (2021). Hsp70 chaperones TDP-43 in dynamic, liquid-like phase and prevents it from amyloid aggregation. Cell Res.

[38] Koehler LC, Grese ZR, Bastos ACS, Mamede LD, Heyduk T, Ayala YM (2022). TDP-43 oligomerization and phase separation properties are necessary for autoregulation. Front Neurosci.

[39] Takahashi M, Kitaura H, Kakita A, Kakihana T, Katsuragi Y, Onodera O (2022). USP10 inhibits aberrant cytoplasmic aggregation of TDP-43 by promoting stress granule clearance. Mol Cell Biol.

[40] Ma XR, Prudencio M, Koike Y, Vatsavayai SC, Kim G, Harbinski F (2022). TDP-43 represses cryptic exon inclusion in the FTD–ALS gene UNC13A. Nature.

[41] Yang C, Tan W, Whittle C, Qiu L, Cao L, Akbarian S (2010). The C-terminal TDP-43 fragments have a high aggregation propensity and harm neurons by a dominant-negative mechanism. PLoS ONE.

[42] Shenoy J, El Mammeri N, Dutour A, Berbon M, Saad A, Lends A (2020). Structural dissection of amyloid aggregates of TDP-43 and its C-terminal fragments TDP-35 and TDP-16. FEBS J.

[43] Wei Y, Lim L, Wang L, Song J (2017). ALS-causing cleavages of TDP-43 abolish its RRM2 structure and unlock CTD for enhanced aggregation and toxicity. Biochem Biophys Res Commun.

[44] Liu-Yesucevitz L, Bilgutay A, Zhang Y-J, Vanderweyde T, Citro A, Mehta T (2010). Tar DNA binding protein-43 (TDP-43) associates with stress granules: analysis of cultured cells and pathological brain tissue. PLoS ONE.

[45] Colombrita C, Zennaro E, Fallini C, Weber M, Sommacal A, Buratti E (2009). TDP-43 is recruited to stress granules in conditions of oxidative insult. J Neurochem.

[46] McGurk L, Lee VM, Trojanowski JQ, Van Deerlin VM, Lee EB, Bonini NM (2014). Poly-A binding protein-1 localization to a subset of TDP-43 inclusions in amyotrophic lateral sclerosis occurs more frequently in patients harboring an expansion in C9orf72. J Neuropathol Exp Neurol.

[47] Bentmann E, Neumann M, Tahirovic S, Rodde R, Dormann D, Haass C (2012). Requirements for stress granule recruitment of fused in sarcoma (FUS) and TAR DNA-binding protein of 43 kDa (TDP-43). J Biol Chem.

[48] Ratti A, Gumina V, Lenzi P, Bossolasco P, Fulceri F, Volpe C (2020). Chronic stress induces formation of stress granules and pathological TDP-43 aggregates in human ALS fibroblasts and iPSC-motoneurons. Neurobiol Dis.

[49] Dewey CM, Cenik B, Sephton CF, Dries DR, Mayer P, Good SK (2011). TDP-43 is directed to stress granules by sorbitol, a novel physiological osmotic and oxidative stressor. Mol Cell Biol.

[50] Goh CW, Lee IC, Sundaram JR, George SE, Yusoff P, Brush MH (2018). Chronic oxidative stress promotes GADD34-mediated phosphorylation of the TAR DNA-binding protein TDP-43, a modification linked to neurodegeneration. J Biol Chem.

[51] Wang W, Wang L, Lu J, Siedlak SL, Fujioka H, Liang J (2016). The inhibition of TDP-43 mitochondrial localization blocks its neuronal toxicity. Nat Med.

[52] Lei Y, Zhang Z-F, Lei R-X, Wang S, Zhuang Y, Liu A-C (2018). DJ-1 suppresses cytoplasmic TDP-43 aggregation in oxidative stress-induced cell injury. J Alzheimers Dis.

[53] Zuo X, Zhou J, Li Y, Wu K, Chen Z, Luo Z (2021). TDP-43 aggregation induced by oxidative stress causes global mitochondrial imbalance in ALS. Nat Struct Mol Biol.

[54] Iguchi Y, Katsuno M, Takagi S, Ishigaki S, Niwa J, Hasegawa M (2012). Oxidative stress induced by glutathione depletion reproduces pathological modifications of TDP-43 linked to TDP-43 proteinopathies. Neurobiol Dis.

[55] Ayala V, Granado-Serrano AB, Cacabelos D, Naudí A, Ilieva EV, Boada J (2011). Cell stress induces TDP-43 pathological changes associated with ERK1/2 dysfunction: implications in ALS. Acta Neuropathol.

[56] Mazan-Mamczarz K, Lal A, Martindale JL, Kawai T, Gorospe M (2006). Translational repression by RNA-binding protein TIAR. Mol Cell Biol.

[57] Khalfallah Y, Kuta R, Grasmuck C, Prat A, Durham HD, Vande Velde C (2018). TDP-43 regulation of stress granule dynamics in neurodegenerative disease-relevant cell types. Sci Rep.

[58] Fang MY, Markmiller S, Vu AQ, Javaherian A, Dowdle WE, Jolivet P (2019). Small-molecule modulation of TDP-43 recruitment to stress granules prevents persistent TDP-43 accumulation in ALS/FTD. Neuron.

[59] Zhang P, Fan B, Yang P, Temirov J, Messing J, Kim HJ (2019). Chronic optogenetic induction of stress granules is cytotoxic and reveals the evolution of ALS-FTD pathology. Elife.

[60] Watkins JA, Alix JJP, Shaw PJ, Mead RJ (2021). Extensive phenotypic characterisation of a human TDP-43Q331K transgenic mouse model of amyotrophic lateral sclerosis (ALS). Sci Rep.

[61] Winton MJ, Igaz LM, Wong MM, Kwong LK, Trojanowski JQ, Lee VMY (2008). Disturbance of nuclear and cytoplasmic TAR DNA-binding protein (TDP-43) induces disease-like redistribution, sequestration, and aggregate formation. J Biol Chem.

[62] Porta S, Xu Y, Restrepo CR, Kwong LK, Zhang B, Brown HJ (2018). Patient-derived frontotemporal lobar degeneration brain extracts induce formation and spreading of TDP-43 pathology in vivo. Nat Commun.

[63] Lu S, Hu J, Aladesuyi B, Goginashvili A, Vazquez-Sanchez S, Diedrich J (2021). Heat shock chaperone HSPB1 regulates cytoplasmic TDP-43 phase separation and liquid-to-gel transition. Nat Cell Biol.

[64] Che M-X, Jiang L-L, Li H-Y, Jiang Y-J, Hu H-Y (2015). TDP-35 sequesters TDP-43 into cytoplasmic inclusions through binding with RNA. FEBS Lett.

[65] Maurel C, Madji-Hounoum B, Thepault R-A, Marouillat S, Brulard C, Danel-Brunaud V (2018). Mutation in the RRM2 domain of TDP-43 in Amyotrophic Lateral Sclerosis with rapid progression associated with ubiquitin positive aggregates in cultured motor neurons. Amyotroph Lateral Scler Frontotemporal Degener.

[66] Lin Y-C, Boone M, Meuris L, Lemmens I, Van Roy N, Soete A (2014). Genome dynamics of the human embryonic kidney 293 lineage in response to cell biology manipulations. Nat Commun.

[67] Lambert GG, Depernet H, Gotthard G, Schultz DT, Navizet I, Lambert T (2020). *Aequorea*’s secrets revealed: new fluorescent proteins with unique properties for bioimaging and biosensing. PLoS Biol.

[68] Lee Y-B, Scotter EL, Lee D-Y, Troakes C, Mitchell J, Rogelj B (2022). Cytoplasmic TDP-43 is involved in cell fate during stress recovery. Hum Mol Genet.

[69] Walker AK, Soo KY, Sundaramoorthy V, Parakh S, Ma Y, Farg MA (2013). ALS-associated TDP-43 induces endoplasmic reticulum stress, which drives cytoplasmic TDP-43 accumulation and stress granule formation. PLoS ONE.

[70] Li YR, King OD, Shorter J, Gitler AD (2013). Stress granules as crucibles of ALS pathogenesis. J Cell Biol.

[71] Wang C, Duan Y, Duan G, Wang Q, Zhang K, Deng X (2020). Stress induces dynamic, cytotoxicity-antagonizing TDP-43 nuclear bodies via paraspeckle LncRNA NEAT1-mediated liquid–liquid phase separation. Mol Cell.

[72] Narcís JO, Tapia O, Tarabal O, Piedrafita L, Calderó J, Berciano MT (2018). Accumulation of poly(A) RNA in nuclear granules enriched in Sam68 in motor neurons from the SMNΔ7 mouse model of SMA. Sci Rep.

[73] Markmiller S, Sathe S, Server KL, Nguyen TB, Fulzele A, Cody N (2021). Persistent mRNA localization defects and cell death in ALS neurons caused by transient cellular stress. Cell Rep.

[74] Cohen TJ, Hwang AW, Unger T, Trojanowski JQ, Lee VMY (2012). Redox signalling directly regulates TDP-43 via cysteine oxidation and disulphide cross-linking. EMBO J.

[75] Chou C-C, Zhang Y, Umoh ME, Vaughan SW, Lorenzini I, Liu F (2018). TDP-43 pathology disrupts nuclear pore complexes and nucleocytoplasmic transport in ALS/FTD. Nat Neurosci.

[76] Zhang Y-J, Caulfield T, Xu Y-F, Gendron TF, Hubbard J, Stetler C (2013). The dual functions of the extreme N-terminus of TDP-43 in regulating its biological activity and inclusion formation. Hum Mol Genet.

[77] Jiang L-L, Guan W-L, Wang J-Y, Zhang S-X, Hu H-Y (2022). RNA-assisted sequestration of RNA-binding proteins by cytoplasmic inclusions of the C-terminal 35-kDa fragment of TDP-43. J Cell Sci.

[78] Brettschneider J, Del Tredici K, Toledo JB, Robinson JL, Irwin DJ, Grossman M (2013). Stages of pTDP-43 pathology in amyotrophic lateral sclerosis. Ann Neurol.

[79] McAleese KE, Walker L, Erskine D, Thomas AJ, McKeith IG, Attems J (2017). TDP-43 pathology in Alzheimer’s disease, dementia with Lewy bodies and ageing. Brain Pathol.

[80] Uchino A, Takao M, Hatsuta H, Sumikura H, Nakano Y, Nogami A (2015). Incidence and extent of TDP-43 accumulation in aging human brain. Acta Neuropathol Commun.

[81] Suk TR, Rousseaux MWC (2020). The role of TDP-43 mislocalization in amyotrophic lateral sclerosis. Mol Neurodegener.

[82] Bademosi AT, Walker AK (2022). Cryptic inclusions UNCover losses driving neurodegeneration. Trends Genet.

[83] Chua JP, Bedi K, Paulsen MT, Ljungman M, Tank EMH, Kim ES (2022). Myotubularin-related phosphatase 5 is a critical determinant of autophagy in neurons. Curr Biol.

[84] Petel Légaré V, Rampal CJ, Gurberg TJN, Harji ZA, Allard-Chamard X, Rodríguez EC (2022). Development of an endogenously myc-tagged TARDBP (TDP-43) zebrafish model using the CRISPR/Cas9 system and homology directed repair. Comp Biochem Physiol B Biochem Mol Biol.

[85] Gavrikov AS, Baranov MS, Mishin AS (2020). Live-cell nanoscopy with spontaneous blinking of conventional green fluorescent proteins. Biochem Biophys Res Commun.

[86] Paix A, Folkmann A, Goldman DH, Kulaga H, Grzelak MJ, Rasoloson D (2017). Precision genome editing using synthesis-dependent repair of Cas9-induced DNA breaks. Proc Natl Acad Sci.

[87] Atkinson R, Leung J, Bender J, Kirkcaldie M, Vickers J, King A (2021). TDP-43 mislocalization drives neurofilament changes in a novel model of TDP-43 proteinopathy. Dis Model Mech.

[88] Koulouras G, Panagopoulos A, Rapsomaniki MA, Giakoumakis NN, Taraviras S, Lygerou Z (2018). EasyFRAP-web: a web-based tool for the analysis of fluorescence recovery after photobleaching data. Nucleic Acids Res.

[89] McQuin C, Goodman A, Chernyshev V, Kamentsky L, Cimini BA, Karhohs KW (2018). Cell profiler 3.0: next-generation image processing for biology. PLoS Biol.

