# Supplementary material for: Aggregation-prone TDP-43 sequesters and drives pathological transitions of free nuclear TDP-43
Source: Cell Mol Life Sci. 2023 Mar 17;80(4):95. doi: 10.1007/s00018-023-04739-2 (PMC10023653; doi:10.1007/s00018-023-04739-2)

Aggregation-prone TDP-43 sequesters and drives pathological transitions of free nuclear TDP-43

Author list and affiliations:

Sean S. Keating[1]

Adekunle T. Bademosi[1],

Rebecca San Gil[1,*]

Adam K. Walker[1,*]

[1]Neurodegeneration Pathobiology Laboratory, Queensland Brain Institute, The University of Queensland, St Lucia, QLD 4072, Australia

* corresponding authors: r.sangil@uq.edu.au, adam.walker@uq.edu.au

Supplementary Materials

A mAvicFP1 insertion HR template

5’-
ATGGAGGCTTTGGCTCAAGCATGGATTCTAAGTCTTCTGGCTGGGGAATGGACTACAAAGACGATGACGACAAGGAA
CAAAAACTCATCTCAGAAGAGGATCTGATGAGCAAAGGAGCAGAGCTTTTCAACGGGATTGTTCCAATTCTTATTGAA
TTAAATGGTGATGTTCACGGACACAAATTCTCTGTTAGAGGAGAGGGAGAAGGCGATGCCGGATCTGGAAAAATTGA
AATCAAATTTGTTTGCACTACTGGTACACTTCCTGTGCCATGGCCCACCCTTGTCACCACATTGTGTTATGGTGTCCA
GTGCTTCACTCGATACCCAGAACACATGAAACAGCATGATTTCTACAAGAGTGCCATGCCAGACGGTTACATACAAAA
AAGAACTATCAGTTTCCAAGACGACGGGCACTACAAGACACGTGCTGAAGTCAAGTTTGAAGGTGATACCCTTGTTAA
TAGAATCGAGTTAAAAGGTATTGATTTTAAAGAAGATGGAAACATTCTTGGAAATAAAATGGAATACAACTATAACTCA
CATTCTGTATACGTCTTGTCAGACAAAGCAAACAATGGAATCAAAGTGAACTTCAAGATTAGACACAACCTTAAAGGT
GAAGGTATACAACTTGCAGACCACGATCAACAAAATATTCCTATTGGTGATGGTCCTGTTCTTCTACCAGACTATCATT
ACCTCTCCACGCAAACTAAAATAACTAAAGATCCTAATGAAAAGAGAGACCACATGAACCTGGTCGAGTTTGTGACAG
CTTGTGGAATCACCCATGGCATGGATGAGTTGTACAAGTAATAGACAGTGGGGTTGTGGTTGGTTGGTATAGAATGG
TGGGAATTCAAATTTTTCTA-3’

B mCherry insertion HR template

5’-
ATGGAGGCTTTGGCTCAAGCATGGATTCTAAGTCTTCTGGCTGGGGAATGGACTACAAAGACGATGACGACAAGGAA
CAAAAACTCATCTCAGAAGAGGATCTGATGGTGAGCAAGGGCGAGGAGGATAACATGGCCATCATCAAGGAGTTCAT
GCGCTTCAAGGTGCACATGGAGGGCTCCGTGAACGGCCACGAGTTCGAGATCGAGGGCGAGGGCGAGGGCCGCC
CCTACGAGGGCACCCAGACCGCCAAGCTGAAGGTGACCAAGGGTGGCCCCCTGCCCTTCGCCTGGGACATCCTGT
CCCCTCAGTTCATGTACGGCTCCAAGGCCTACGTGAAGCACCCCGCCGACATCCCCGACTACTTGAAGCTGTCCTTC
CCCGAGGGCTTCAAGTGGGAGCGCGTGATGAACTTCGAGGACGGCGGCGTGGTGACCGTGACCCAGGACTCCTCC
CTGCAGGACGGCGAGTTCATCTACAAGGTGAAGCTGCGCGGCACCAACTTCCCCTCCGACGGCCCCGTAATGCAGA
AGAAGACCATGGGCTGGGAGGCCTCCTCCGAGCGGATGTACCCCGAGGACGGCGCCCTGAAGGGCGAGATCAAGC
AGAGGCTGAAGCTGAAGGACGGCGGCCACTACGACGCTGAGGTCAAGACCACCTACAAGGCCAAGAAGCCCGTGC
AGCTGCCCGGCGCCTACAACGTCAACATCAAGTTGGACATCACCTCCCACAACGAGGACTACACCATCGTGGAACA
GTACGAACGCGCCGAGGGCCGCCACTCCACCGGCGGCATGGACGAGCTGTACAAGTAATAGACAGTGGGGTTGTG
GTTGGTTGGTATAGAATGGTGGGAATTCAAATTTTTCTA-3’

C HR template PCR amplification primers

- FWD: 5’-ATGGAGGCTTTGGCTCAA-3’
- RVS: 5’-ATCTTTTAAACTTAAGGG-3’

Supplementary Fig. S1 (relating to Fig. 1): *TARDBP* C-terminal fluorescent tag homologous recombination (HR) DNA templates for CRISPR/Cas9 homology-directed repair. (A) *mAvicFP1* (green) and **(B)** *mCherry* (red) tag sequences, preceded by *FLAG* (blue) and *MYC* (purple) linkers, flanked by homology arms (grey). **(C)** PCR primers for amplifcation of HR template DNA fragments.

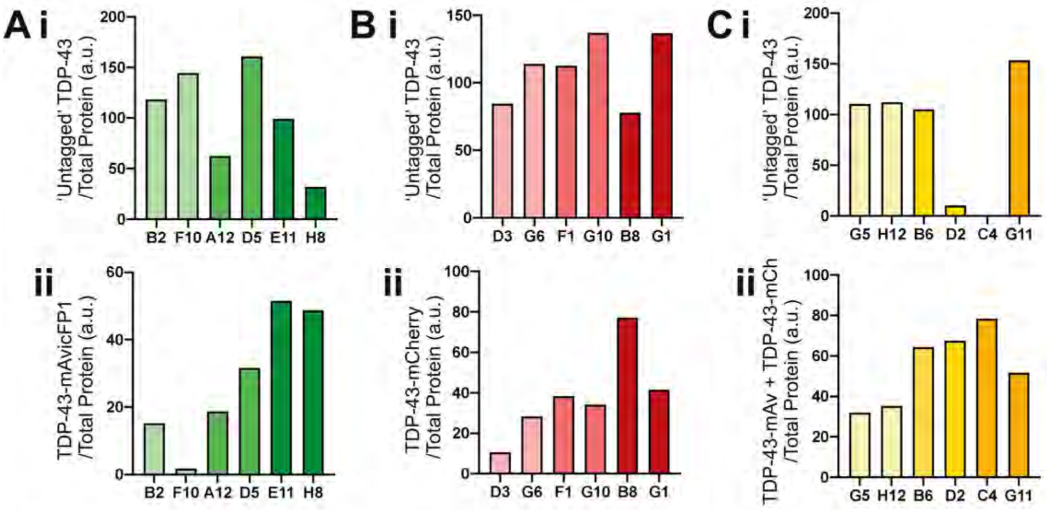

D

$$TARDBP \text{ editing ratio} = \frac{\text{'Tagged' TDP-43 (72/74 kDa)}}{\text{'Tagged' + 'Untagged' TDP-43 (43 kDa)}}$$

Supplementary Fig. S2 (relating to Fig. 2): Quantification of *TARDBP* ‘editing ratios’ from top candidate CRISPR-tagged endogenous TDP-43 monoclonal HEK293 cell lines. Densitometry analysis for **(i)** ‘untagged’ TDP-43 (43 kDa) band, or **(ii)** ‘CRISPR-tagged’ TDP-43 (72-74 kDa) normalised to total protein signal for monoclonal **(A)** *TARDBP-mAvicFP1*, **(B)** *TARDBP-mCherry*, or **(C)** *TARDBP-mAv/-mCh* cell lines. **(D)** Formula for calculation of *TARDBP* editing ratios.

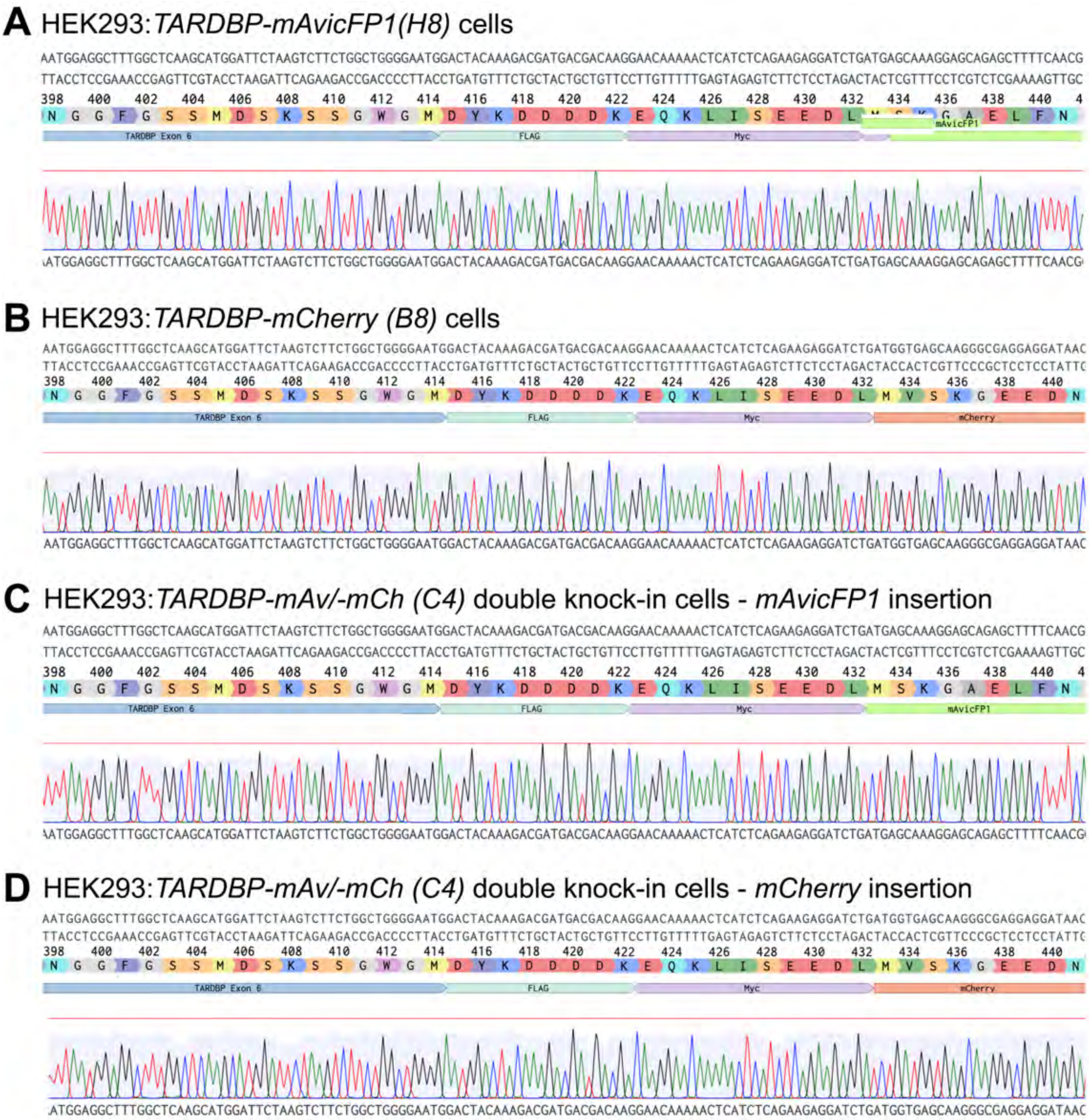

<u>Supplementary Fig. S3 (relation to Fig. 3):</u> Sanger sequencing indicates precise and complete homologous recombination of *mAvicFP1* or *mCherry* sequences at desired *TARDBP* C-terminal insertion sites. DNA sequences spanning *TARDBP* C-terminus, insertion site, and partially into *mAvicFP1* or *mCherry* fluorescent tags were PCR-amplified from genomic DNA extracted from top candidate CRISPR-edited **(A)** *TARDBP-mAvicFP1(H8)*, **(B)** *TARDBP-mCherry(B8)*, or **(C,D)** *TARDBP-mAv-mCh(C4)* monoclonal HEK293 cell lines. Top and bottom lines represent sequences amplified from forward and reverse primers, respectively. Sequence alignments performed with *Benchling*.

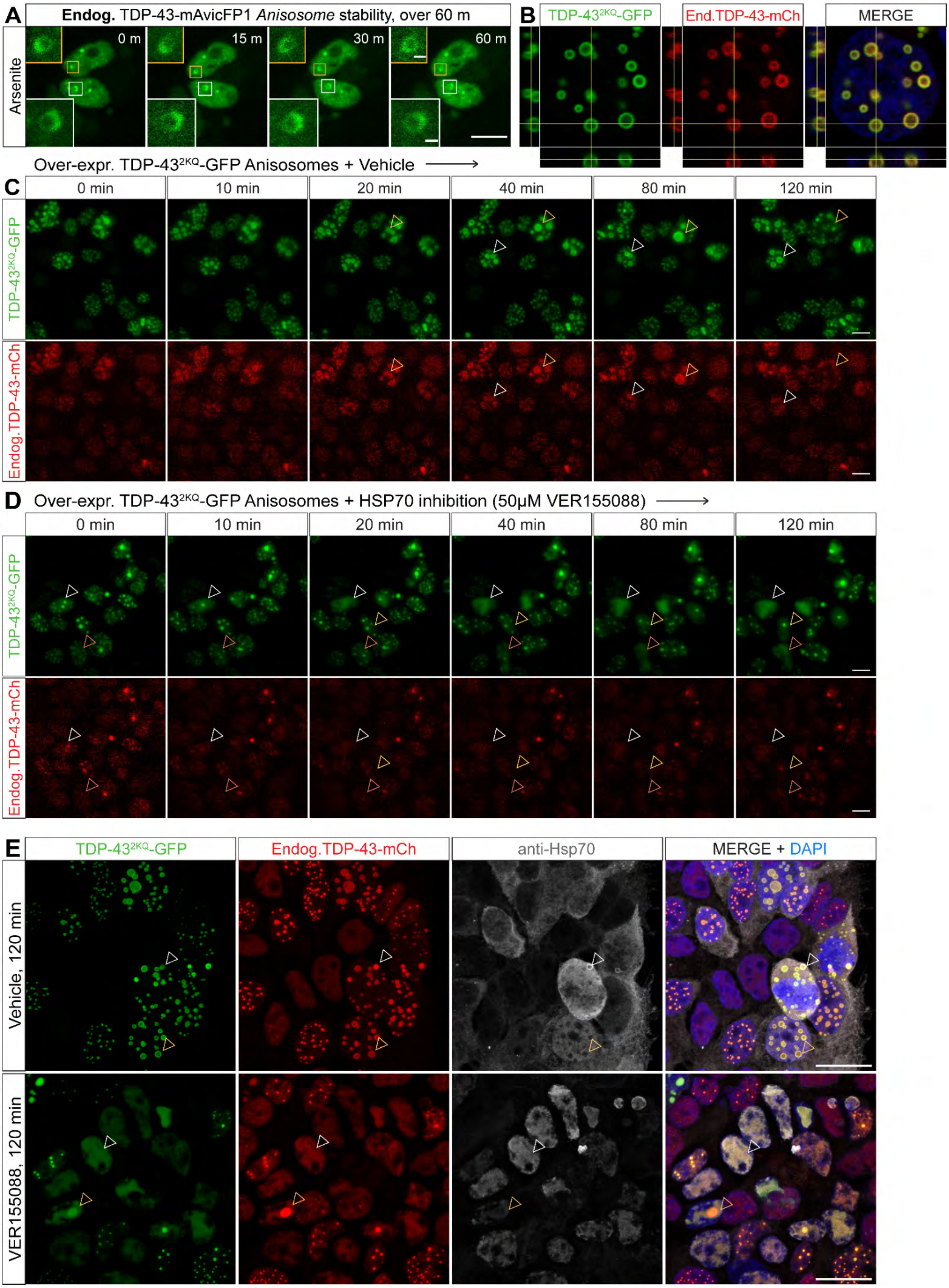
A
Endog. TDP-43-mAvicFP1 Anisosome stability, over 60 m
Arsenite
0 m
15 m
30 m
60 m
B
TDP-43^2KQ-GFP
End.TDP-43-mCh
MERGE
Over-expr. TDP-43^2KQ-GFP Anisosomes + Vehicle
C
0 min
10 min
20 min
40 min
80 min
120 min
TDP-43^2KQ-GFP
Endog.TDP-43-mCh
D
Over-expr. TDP-43^2KQ-GFP Anisosomes + HSP70 inhibition (50μM VER155088)
0 min
10 min
20 min
40 min
80 min
120 min
TDP-43^2KQ-GFP
Endog.TDP-43-mCh
E
TDP-43^2KQ-GFP
Endog.TDP-43-mCh
anti-Hsp70
MERGE + DAPI
Vehicle, 120 min
VER155088, 120 min

<u>Supplementary Fig. S4 (relating to Fig. 4):</u> Verification of HSP70-dependent formation anisosomes comprising endogenous under oxidative stress or with acetylation-mimicking mutation. (A) Arsenite-induced endogenous TDP-43-mAvicFP1 anisosomes under live-cell imaging were stable throughout treatment, over 60 min (scale: large panels = 10 μm, zoomed inset panels = 1 μm). **(B)** 3-dimensional orthogonal projection from confocal Z-stack images of HEK293:*TARDBP-mCh(B8)* cells demonstrates spherical shell-like structure of anisosomes formed by aceylation-mimicking mutant TDP-43^{2KQ}-GFP, which readily sequester endogenous TDP-43-mCherry. **(C)** Live-cell timelapse imaging demonstrating TDP-43^{2KQ}-GFP anisosome formation, fusion (white arrowheads), fission (yellow arrowheads) and endogenous TDP-43-mCherry recruitment under vehicle treatment for 120 min. **(D)** Timelapse of Hsp70-dependent disassembly of anisosomes (colour-coded arrowheads) following treatment with Hsp70 inhibitor, 50 μM VER155088 (scale = 10 μm). **(E)** Confocal Z-stack maximum projection of vehicle- (top panels) or Hsp70 inhibitor-treated (bottom panels) cells immunostained with anti-Hsp70 antibody, which could not penetrate anisosomes (arrowheads) to detect Hsp70 within the core of these assemblies, using a 1% Triton X-100 permeabilisation protocol. Hsp70-dependent anisosome disassembly results in mostly diffuse TDP-43 (white arrowheads), or can leave large condensed nuclear structures that still sequester endogenous TDP-43 (yellow arrowheads, scale = 20 μm).

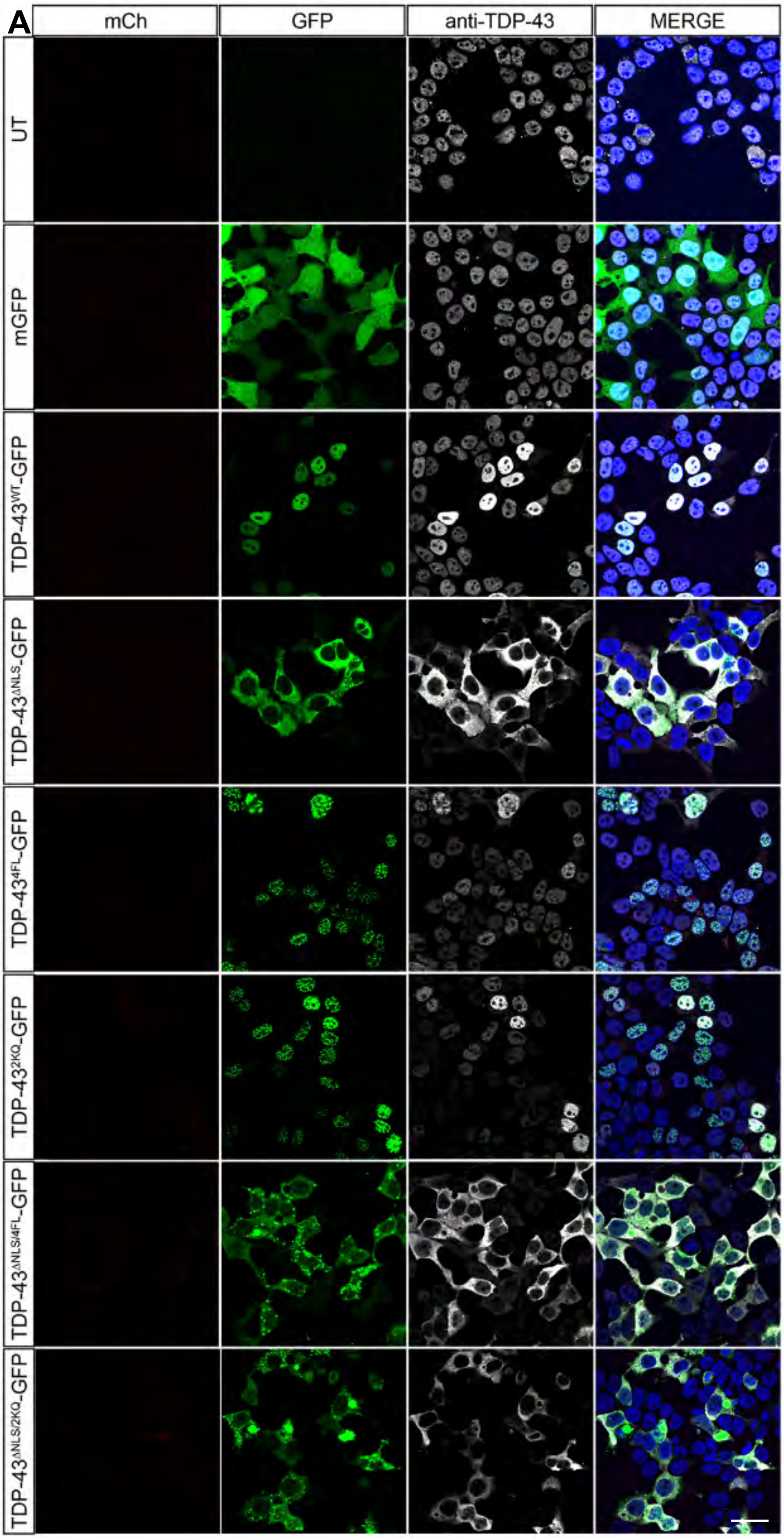

<u>Supplementary Fig. S5 (relating to Fig. 5):</u> Bleed-through control images of TDP-43-mCh-negative parental HEK293 cells over-expressing TDP-43-GFP variants. (A) Confocal imaging verified no bleed-through fluorescence of GFP signal into mCherry channel, used for image analysis thresholding of endogenous TDP-43-mCherry sequestration. Anti-TDP-43 antibody immunostaining is directed towards over-expressed TDP-43-GFP proteins, and does not penetrate inclusion structures (scale = 20 μm).

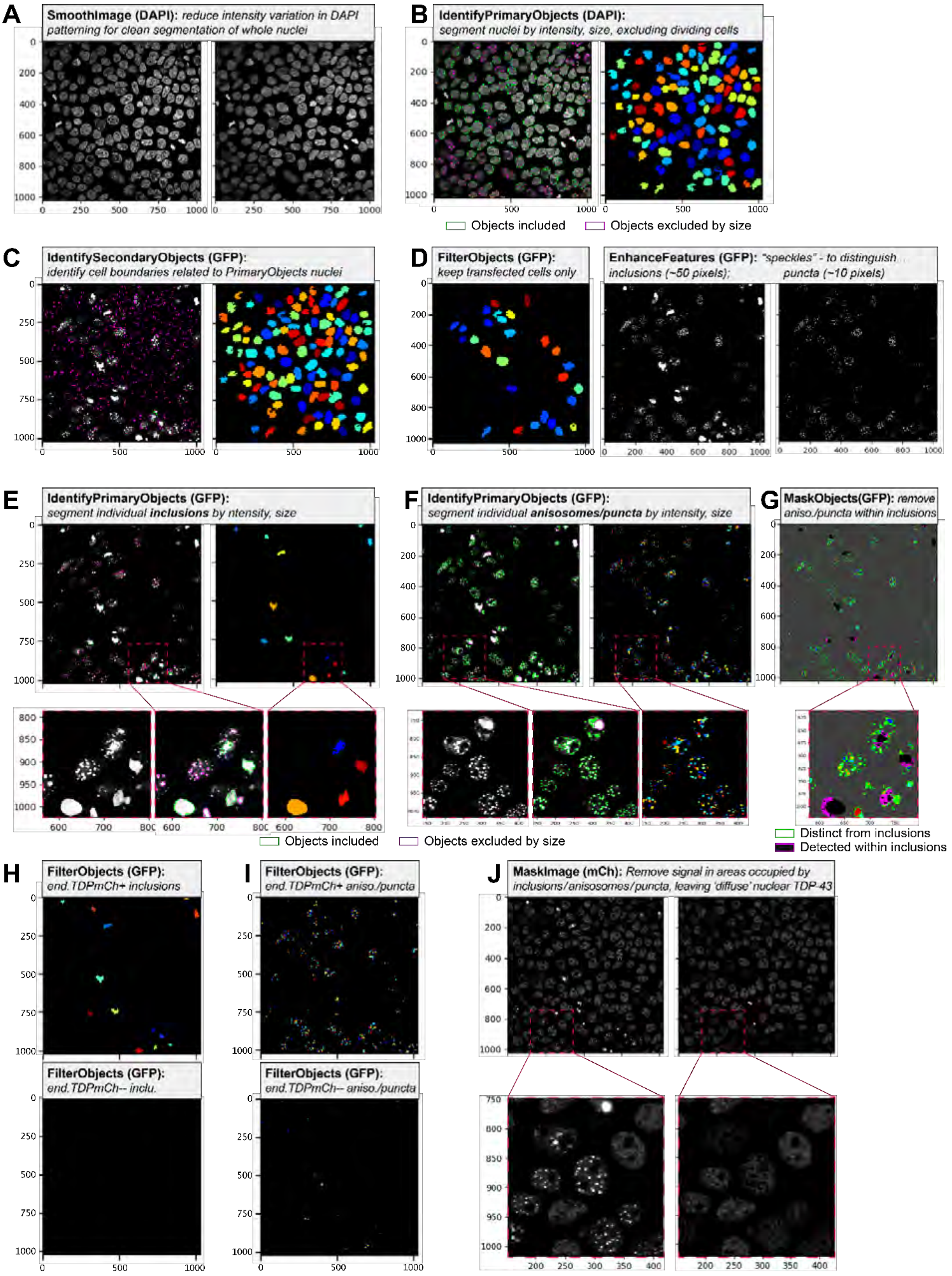

<u>**Supplementary Fig. S6 (relating to Fig. 5 & Supplementary Fig. S7):**</u> **CellProfiler pipeline for unbiased and automated segmentation of transfected cells, TDP-43-GFP assemblies, and intensity/morphology measurements. (A)** *SmoothImage* module over DAPI images for **(B)** *IdentifyPrimaryObjects* module to segment whole nuclei, filtered based on mean intensity and size to remove condensed nuclei of dividing cells. **(C)** *IdentifySecondaryObjects* to segment cell boundaries, anchored to primary nuclei. **(D)** Transfected cells filtered based on median GFP intensity. *EnhanceFeatures* module applied to GFP images to distinguish speckles of ~50 or ~10 pixels, representing 'inclusions' or 'anisosomes'/'puncta' respectively, segmented by **(E,F)** *IdentifyPrimaryObjects* modules, wherein inclusions were defined as large bright speckles with intensity above 60% of saturation point and minimum diameter of 4 μm. Anisosomes/puncta were defined as speckles with intensity above 25% of saturation point and diameter of 0.5-4 μm. **(G)** *MaskObjects* module to remove smaller assemblies falsely detected by segmentation of larger

inclusions. **(H)** *FilterObjects* modules to identify endogenous TDP-43-mCherry-positive or -negative inclusions or **(I)** puncta. **(J)** *MaskImage* module to distinguish sequestered from free diffuse nuclear endogenous TDP-43-mCherry.

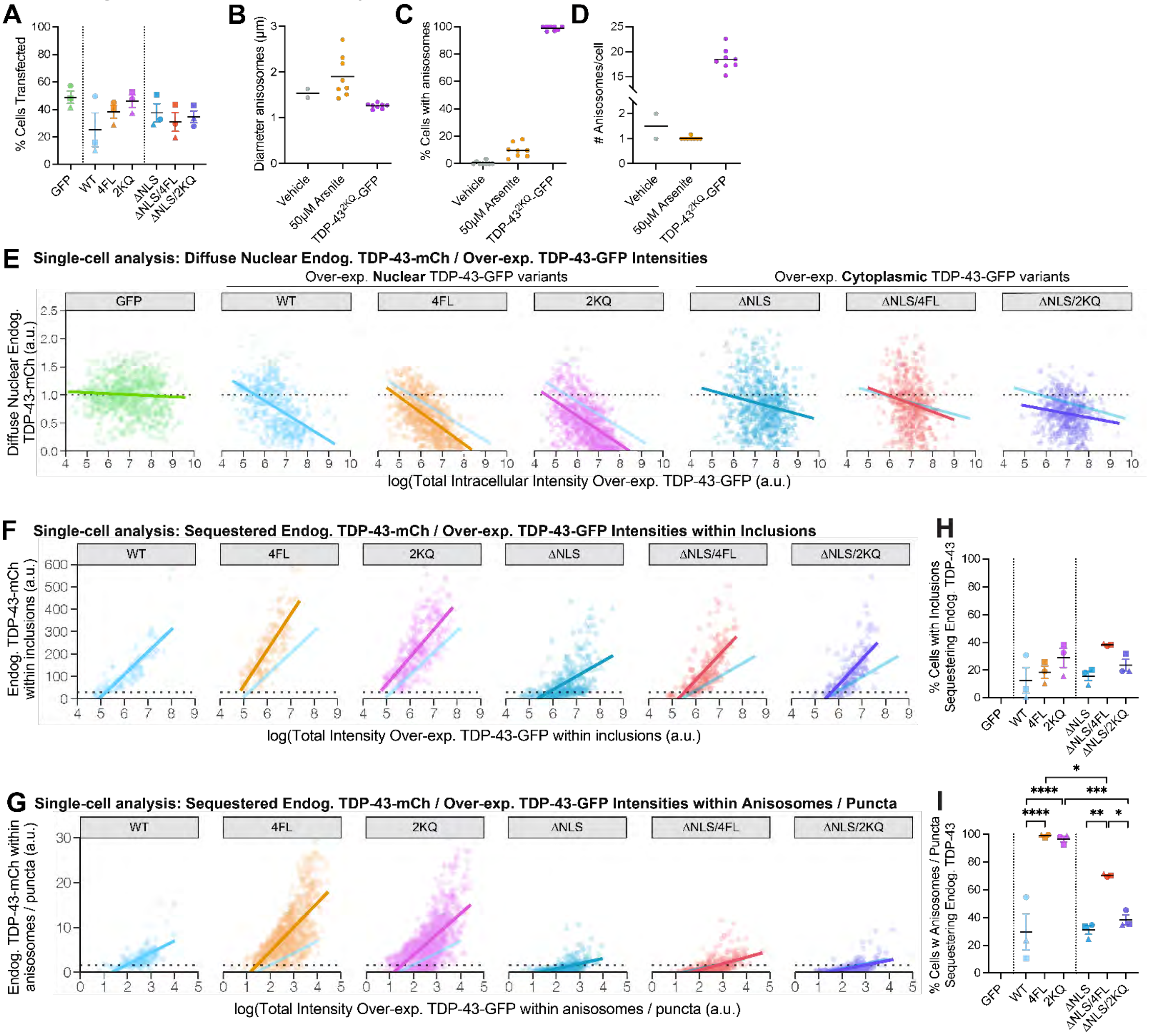

Supplementary Fig. S7 (relating to Fig. 5): Single-cell quantitative image analysis reveals concentration-dependent effect of mutant TDP-43-GFP of assemblies on sequestration and depletion of free nuclear endogenous TDP-43-mCherry. **(A)** Transfection rate for exogenous TDP-43-GFP variants measured by CellProfiler image segmentation analysis. **(B)** Diameter of anisosomes, **(C)** % of cells with anisosomes, and **(D)** number of anisosomes per cell formed by TDP-43^{2KQ}-GFP compared to arsenite-induced endogenous TDP-43 anisosomes. **(B-D)** Data points represent mean from 8 images from a single biological replicate. **(E)** Scatterplots illustrating concentration-dependent effects of over-expressed TDP-43-GFP protein levels (total intensity) on median intensity of diffuse nuclear endogenous TDP-43-mCherry (normalised to GFP-transfected controls), showing individual transfected cells. **(F)** Total intensity of endogenous TDP-43-mCherry sequestered to inclusions, or **(G)** anisosomes/puncta, against total intensity of TDP-43-GFP assemblies per cell. **(E-G)** Linear regressions demonstrate concentration-dependent effects of TDP-43-GFP over-expression on endogenous TDP-43 nuclear depletion or sequestration. Comparison of WT (light blue) or ΔNLS (teal) regression lines overlayed on nuclear or cytoplasmic mutants respectively demonstrates amplified effects of 4FL and 2KQ mutations on depletion and sequestration. Data points represent individual cells segmented with CellProfiler, sampled from 8 imaging regions from each of *n* = 3 biologically independent repeats. **(H)** % Cells with inclusions or **(I)** anisosomes/puncta sequestering endogenous TDP-43-mCherry. **(A,H,I)** Individual points represent mean values from 8 imaging regions of interest for each of *n* = 3 biologically independent

repeats. Black lines represent combined mean values, analysed by ordinary one-way ANOVA (* = *P* 0.05; ** = *P* < 0.01; *** = *P* < 0.001; **** = *P* < 0.0001).

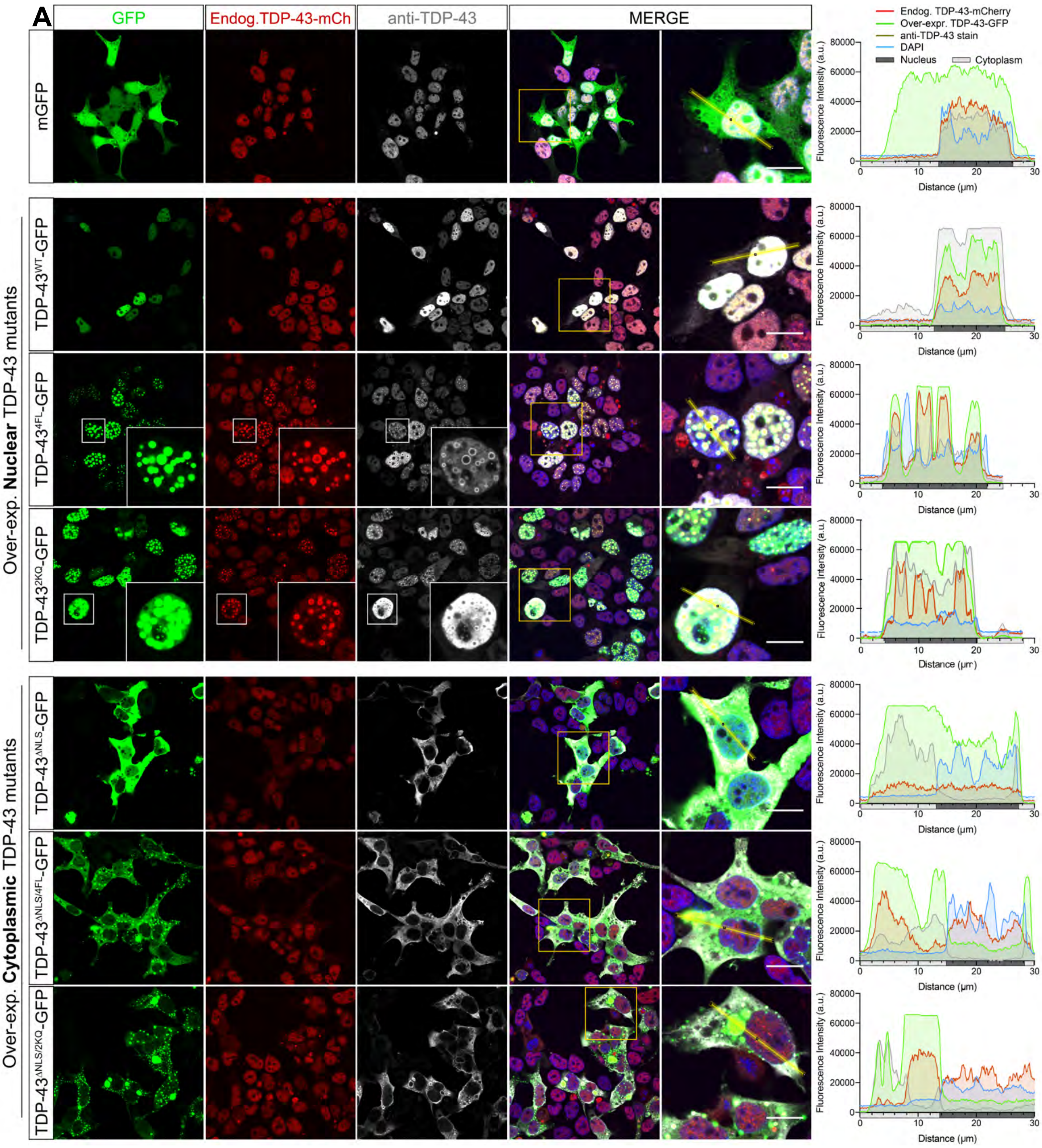

<u>Supplementary Fig. S8 (relating to Fig. 5):</u> Immunostaining with anti-TDP-43 antibody preferentially binds over-expressed TDP-43-GFP variants, necessitating CRISPR-tagging for visualising endogenous TDP-43 separately from pathological forms. (A) Confocal images depict the formation of exogenous TDP-43-GFP structures in the nucleus or cytoplasm and relative localisation of endogenous TDP-43-mCherry and anti-TDP-43 (scale = 10µm). Areas of colocalization indicated by intensity profiles across regions marked by yellow line in right-hand magnified view panels. Anisosomes formed by 4FL and 2KQ mutant TDP-43 variants are highlighted with additional magnified inset panels, demonstrating that anti-TDP-43 antibody only labels anisosomes peripherally to TDP-43-GFP or endogenous TDP-43-mCh signals, and may not penetrate such dynamic but condensed assemblies.

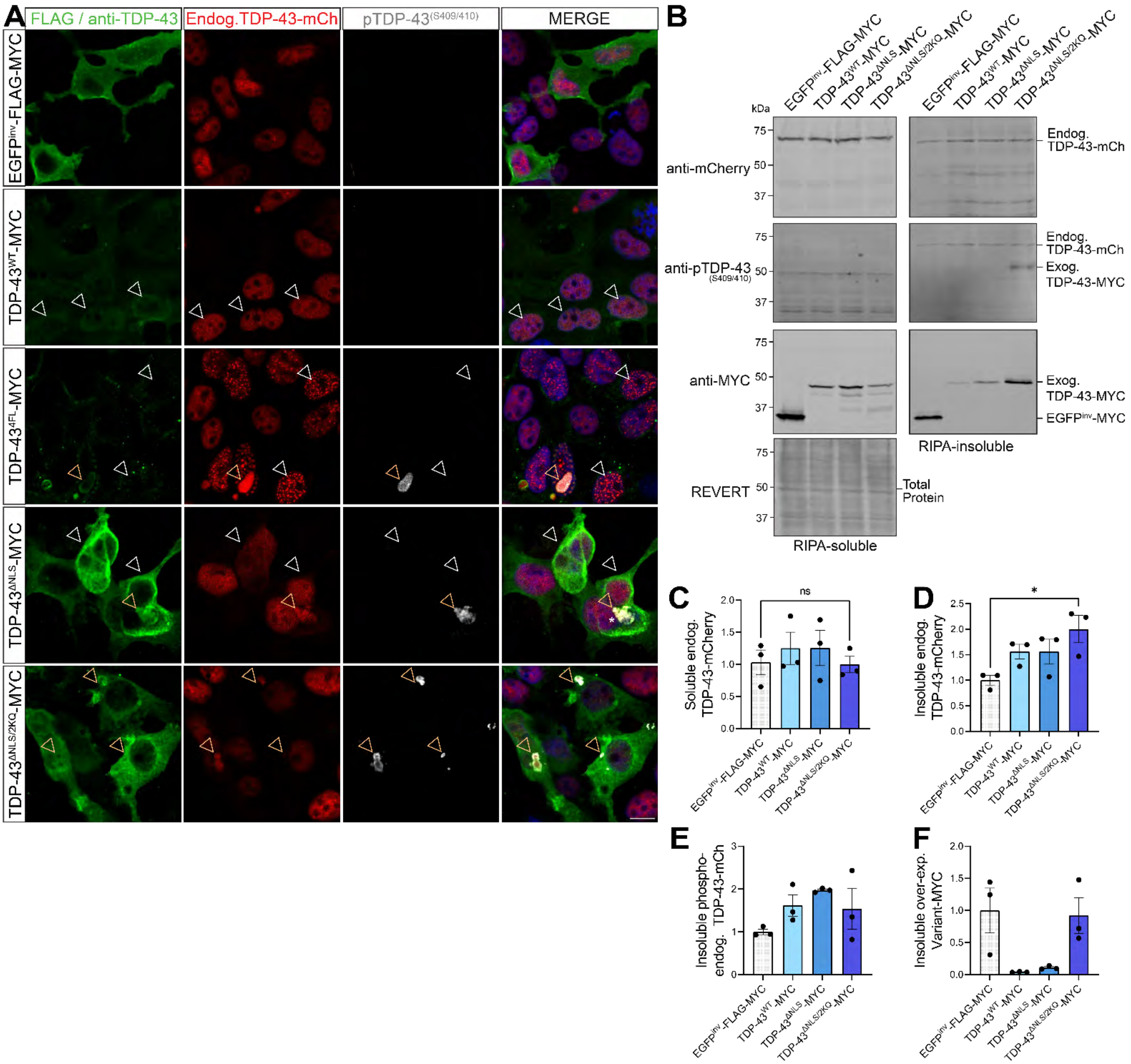

<u>Supplementary Fig. S9 (relating to Fig. 6):</u> Over-expression of aggregation-prone mutant TDP-43 variants lacking fluorescent tags replicates sequestration and insolubility of endogenous TDP-43 driven by TDP-43-GFP variants. (A) Confocal images of HEK293:*TARDBP-mCherry(B8)* cells over-expressing TDP-43-MYC variants (white arrowheads) and assemblies that are phosphorylated and sequester endogenous TDP-43-mCherry (yellow arrowheads). **(B)** Immunoblot of RIPA-soluble and -insoluble protein fractions for mCherry, pTDP-43$^{409/410}$, MYC, and REVERT Total Protein. (C) Densitometry analysis for soluble endogenous TDP-43-mCherry, (D) insoluble endogenous TDP-43-mCherry, (E) insoluble phospho-endogenous TDP-43-mCherry, (F) insoluble over-expressed TDP-43-MYC variant. Data points represent means of *n* = 3 biologically independent repeats, analysed by ordinary one-way ANOVA (* = *P* < 0.05).

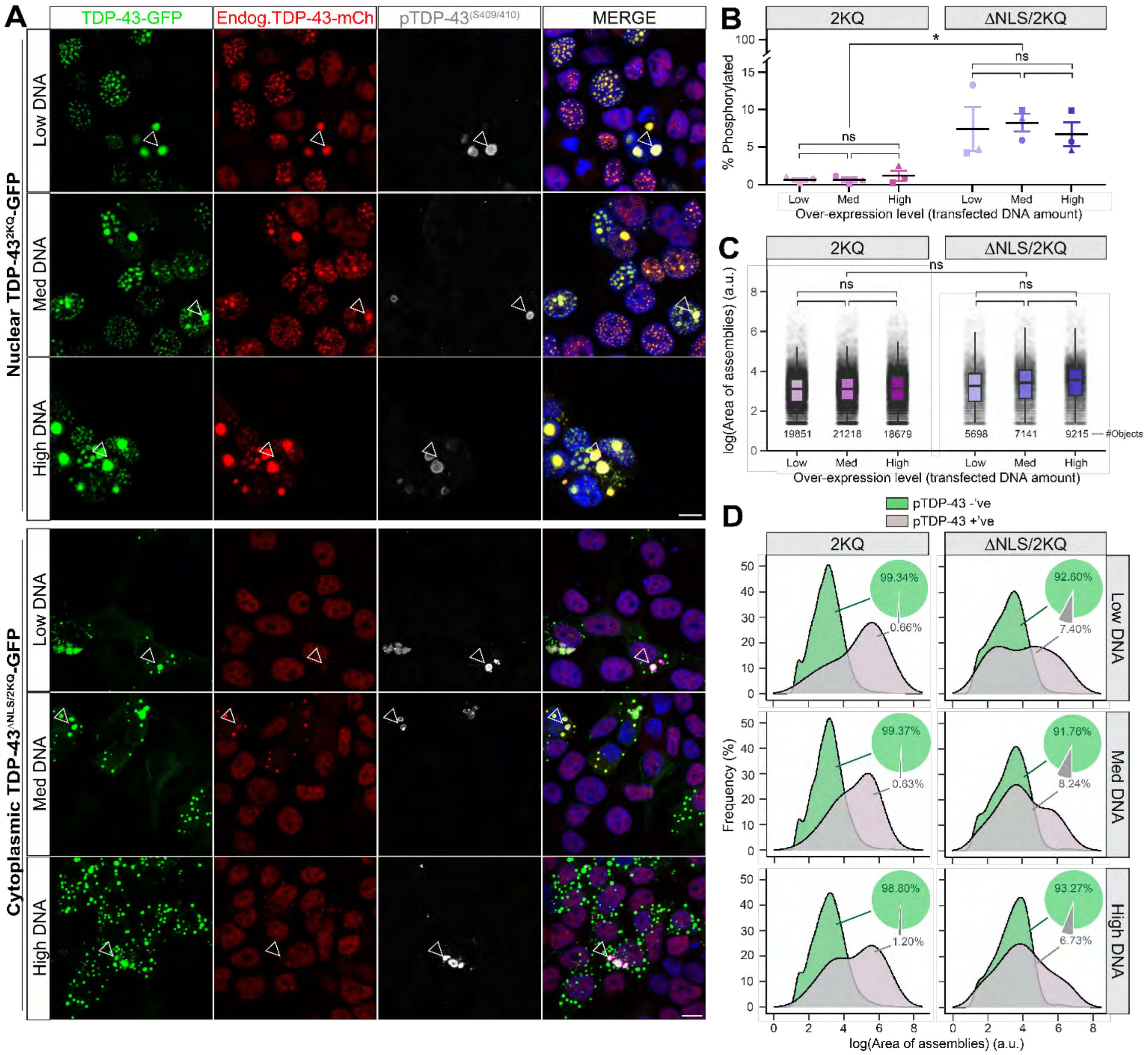

Supplementary Fig. S10 (relating to Fig. 6): Morphological characteristics and phosphorylation of individual mutant TDP-43-GFP assemblies, and their effects on endogenous TDP-43 sequestration, are dependent on the mutant construct and not level of over-expression. **(A)** Maximum intensity projection from confocal Z-stack images displaying TDP-43-GFP assemblies formed by different levels of over-expression in HEK293:*TARDBP-mCh(B8)* cells, immunostained with anti-pTDP-43$^{409/410}$ antibody. White arrowheads indicate phosphorylated assemblies. Transfected DNA amounts "Low", "Med", and "High" equate with 0.25, 0.5, or 1µg DNA per well in a 24-well plate. **(B)** Percentage of 2KQ or ΔNLS/2KQ mutant assemblies containing phospho-TDP-43, for each over-expression level. Black lines represent mean values for 8 imaging regions of interest, from *n* = 3 biologically independent repeats, shown as individual points. Data analysed by ordinary one-way ANOVA (* = $P < 0.05$). **(C)** Boxplot distribution of log-scaled area of assemblies formed by 2KQ or ΔNLS/2KQ mutants. Each point represents individual assemblies totalling from each of *n* = 3 biologically independent repeats (number per group indicated by text below). Statistical analysis completed by ordinary one-way ANOVA, comparing overall mean area of assemblies per group, taken from individual replicate means (ns = $P > 0.05$). **(D)** Frequency distributions of log-scaled area of pTDP-43-positive (grey) or pTDP-43-negative (green) assemblies, for each over-expression level. Pie charts indicate percentage of all assemblies categorised into phosphorylation states.

Other Supplementary Materials

Movie1 (relating to Figure 3)
Representative FRAP acquisition of diffuse nuclear protein under physiological conditions, for endogenous TDP-43-mAvicFP1, over-expressed TDP-43^{WT}-GFP, endogenous TDP-43-mCherry, and over-expressed TDP-43^{WT}-mCherry; scale = 5 μm.

Movie2 (relating to Figure 4)
Representative FRAP acquisition of dynamic TDP-43-mAvicFP1 protein assemblies, including physiological puncta without treatment, arsenite-induced puncta or arsenite-induced anisosomes; scale = 5 μm.

Movie3 (relating to Figure 6)
Representative FRAP acquisition of acetylated TDP-43 assemblies sequestering endogenous TDP-43-mCherry, including cytoplasmic TDP-43$^{\Delta NLS/2KQ}$-GFP inclusions (partial or complete bleaching), nuclear TDP-43^{2KQ}-GFP inclusions, or dynamic nuclear TDP-43^{2KQ}-GFP anisosomes; scale = 5 μm.

Table1 (relating to Figure 3, 4, 6)
Fluorescence recovery after photobleaching raw and normalised data for maximal recovery, time-to-half recovery, area-under-the-curve, and mobility fraction analyses.

Table2 (relating to Figure 5)
CellProfiler unbiased quantitative image analysis pipeline output, values representing per-image means of all transfected cells sampled from 8 regions of interest, imaged at 63x magnification.

Table3 (relating to Supplementary Figure 6)
CellProfiler unbiased quantitative image analysis pipeline output, values representing per-cell values for individual transfected cells sampled from 8 regions of interest, imaged at 63x magnification.

Table4 (relating to Figure 6)
Immunoblot densitometry analysis raw and normalised values. See below immunoblotting uncropped images for reference to the representative blots and cropped regions incorporated to Figure 6.

Uncropped Immunoblots

Fig. 6C: Endogenous TDP-43 is sequestered by nuclear acetylated TDP-43-GFP

RIPA n1 **REVERT** | RIPA n2 **REVERT**

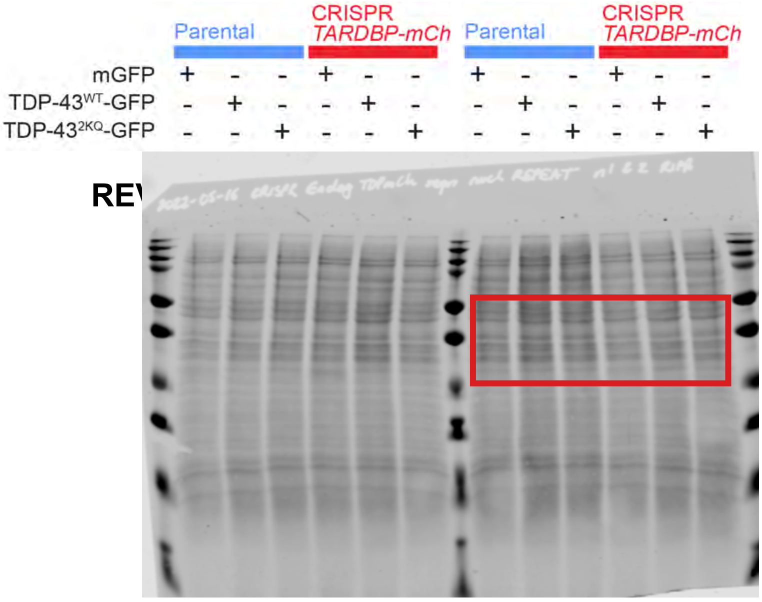

Urea n1 **REVERT** | Urea n2

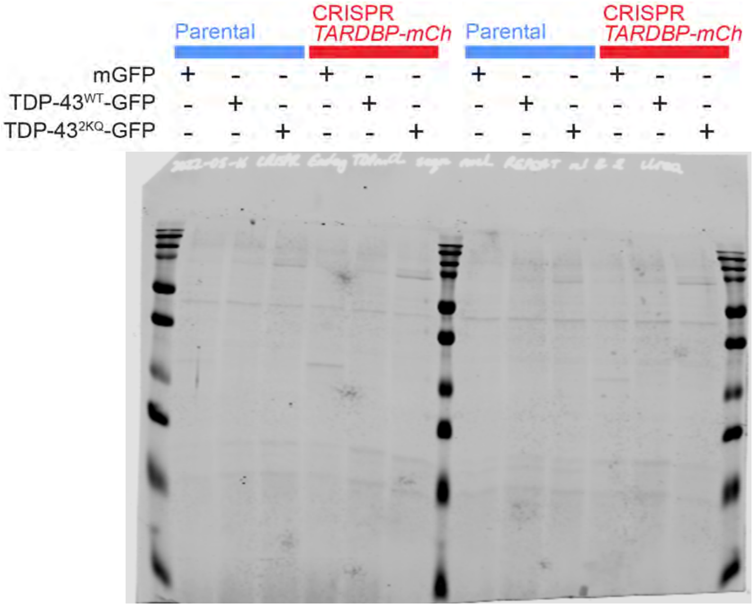

RIPA n3 **REVERT**

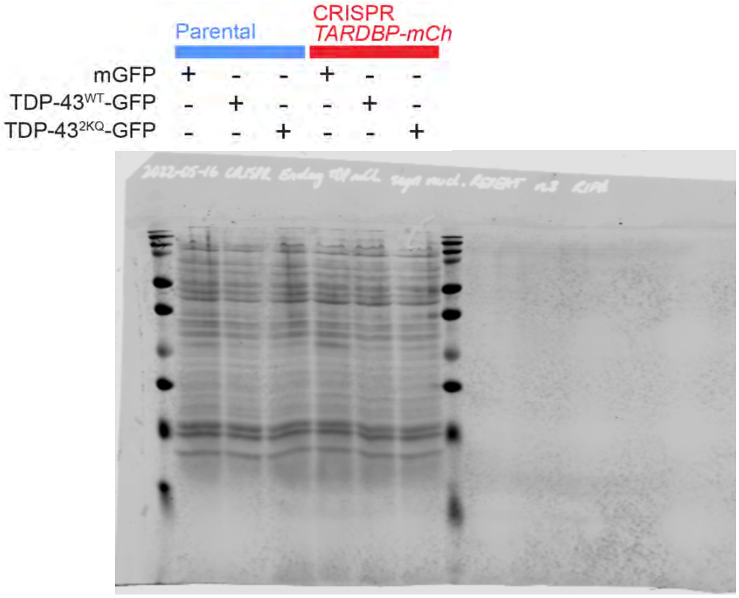

Urea n3 **REVERT**

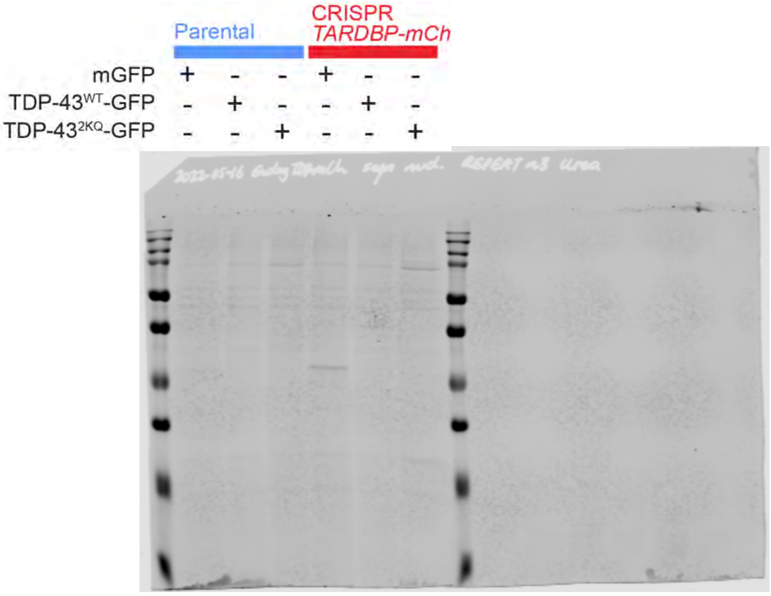

RIPA n1 **TDP-43** | RIPA n2 **TDP-43**

TDP-43

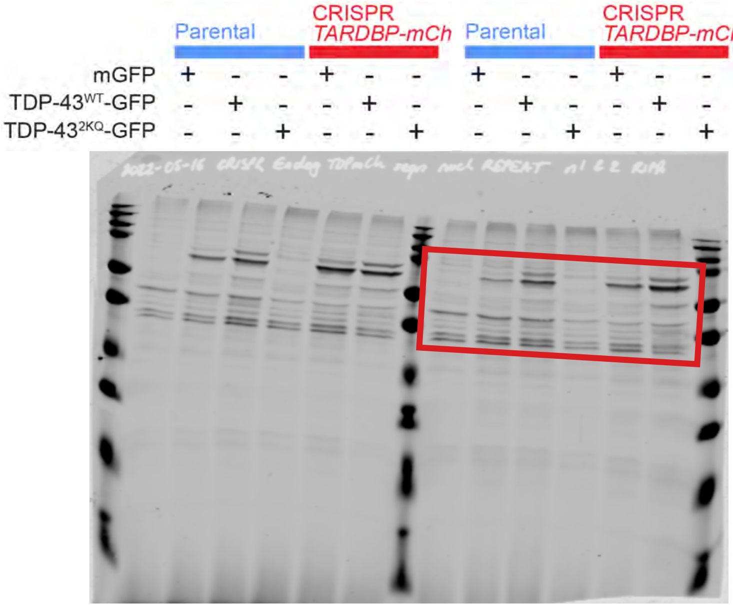

Urea n1 **TDP-43** | Urea n2

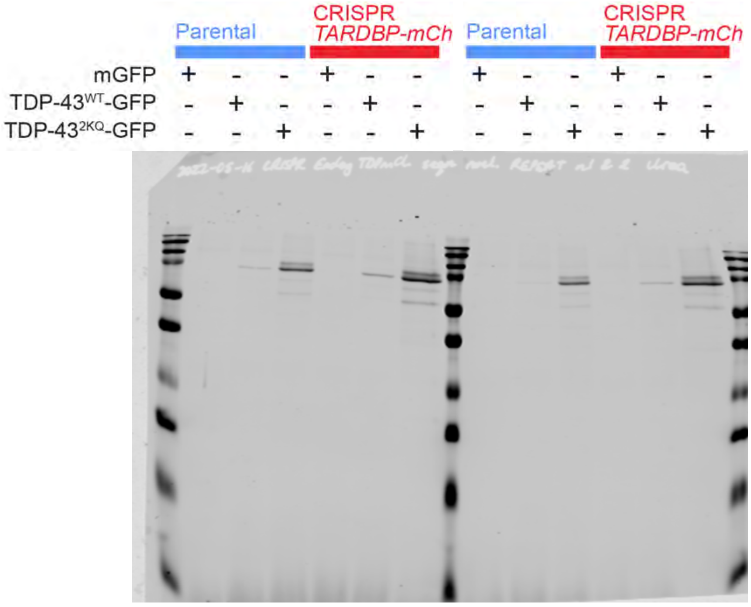

RIPA n3 **TDP-43**

	Parental			CRISPR *TARDBP-mCh*		
mGFP	+	-	-	+	-	-
TDP-43^{WT}-GFP	-	+	-	-	+	-
TDP-43^{2KQ}-GFP	-	-	+	-	-	+

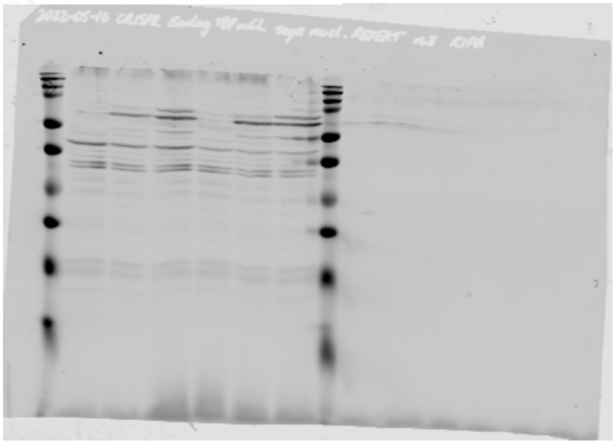

Urea n3 **TDP-43**

	Parental			CRISPR *TARDBP-mCh*		
mGFP	+	-	-	+	-	-
TDP-43^{WT}-GFP	-	+	-	-	+	-
TDP-43^{2KQ}-GFP	-	-	+	-	-	+

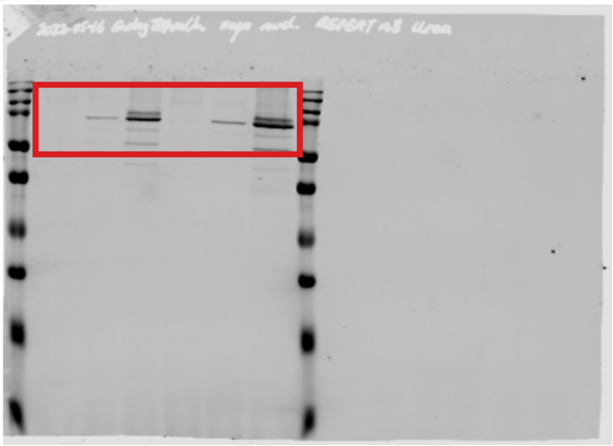

RIPA n1 **MYC** | RIPA n2 **MYC**

	Parental			CRISPR *TARDBP-mCh*			Parental			CRISPR *TARDBP-mCh*		
mGFP	+	-	-	+	-	-	+	-	-	+	-	-
TDP-43^{WT}-GFP	-	+	-	-	+	-	-	+	-	-	+	-
TDP-43^{2KQ}-GFP	-	-	+	-	-	+	-	-	+	-	-	+

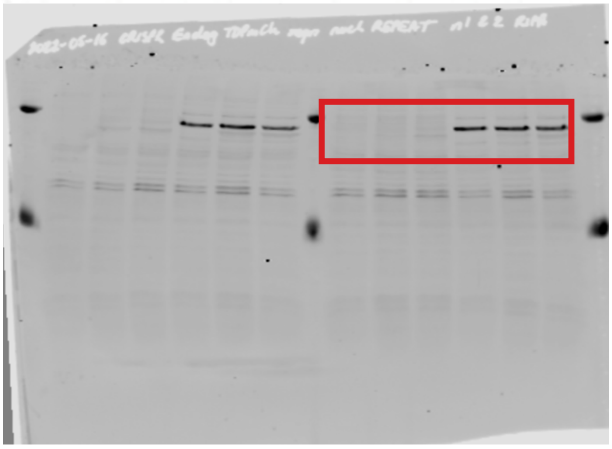

Urea n1 **MYC** | Urea n2

	Parental			CRISPR *TARDBP-mCh*			Parental			CRISPR *TARDBP-mCh*		
mGFP	+	-	-	+	-	-	+	-	-	+	-	-
TDP-43^{WT}-GFP	-	+	-	-	+	-	-	+	-	-	+	-
TDP-43^{2KQ}-GFP	-	-	+	-	-	+	-	-	+	-	-	+

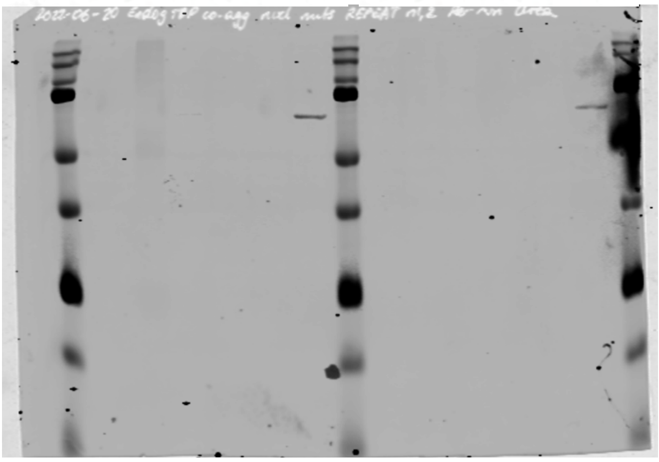

RIPA n3 **MYC**

	Parental			CRISPR *TARDBP-mCh*		
mGFP	+	-	-	+	-	-
TDP-43^{WT}-GFP	-	+	-	-	+	-
TDP-43^{2KQ}-GFP	-	-	+	-	-	+

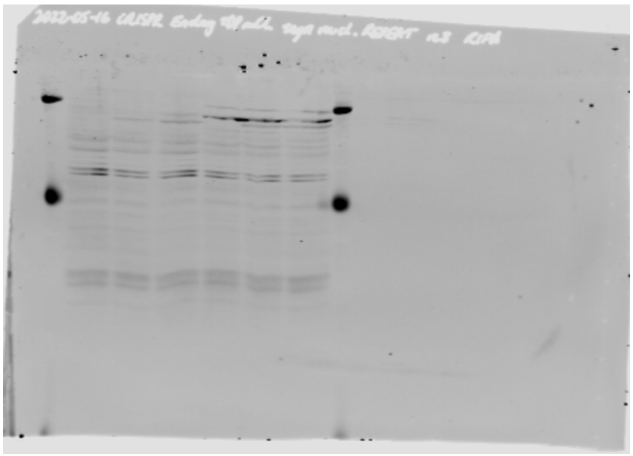

Urea n3 **MYC**

	Parental			CRISPR *TARDBP-mCh*		
mGFP	+	-	-	+	-	-
TDP-43^{WT}-GFP	-	+	-	-	+	-
TDP-43^{2KQ}-GFP	-	-	+	-	-	+

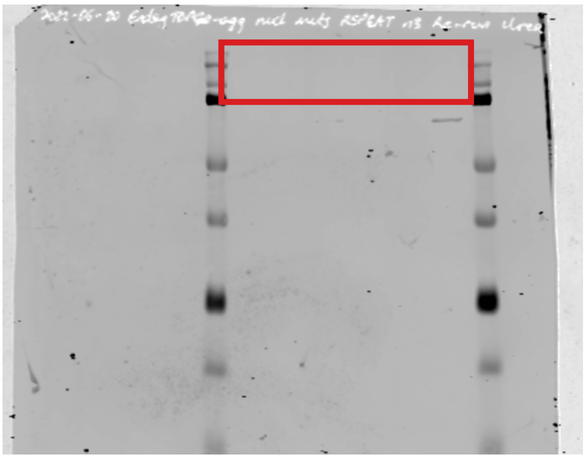

Urea n1 pTDP | Urea n2 pTDP

Parental | CRISPR *TARDBP-mCh* | Parental | CRISPR *TARDBP-mCh*

mGFP + - - + - - + - - + - -
TDP-43^{WT}-GFP - + - - + - - - + - - + -
TDP-43^{2KQ}-GFP - - + - - + - + - + - - +

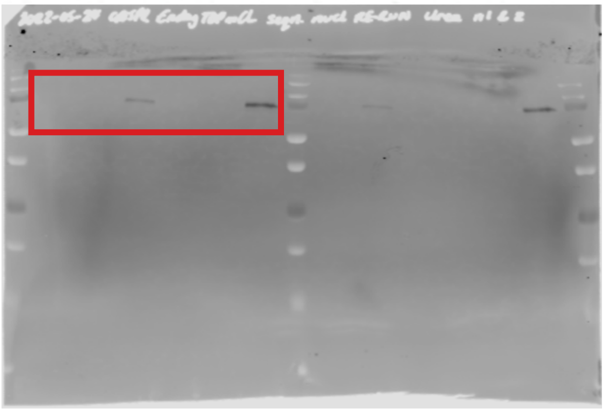

Urea n3 pTDP

Parental | CRISPR *TARDBP-mCh*

mGFP + - - + - -
TDP-43^{WT}-GFP - + - - + -
TDP-43^{2KQ}-GFP - - + - - +

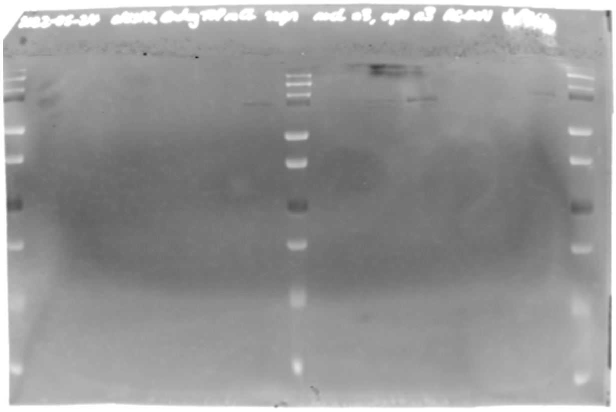

Fig. 6F: Endogenous TDP-43 is sequestered by cytoplasmic acetylated TDP-43-GFP

RIPA n1 **REVERT**

Parental | CRISPR *TARDBP-mCherry*

mGFP + - - + - -
TDP-43$^{\Delta NLS}$-GFP - + - - + -
TDP-43$^{\Delta NLS/2KQ}$-GFP - - + - - + Unrelated experiment

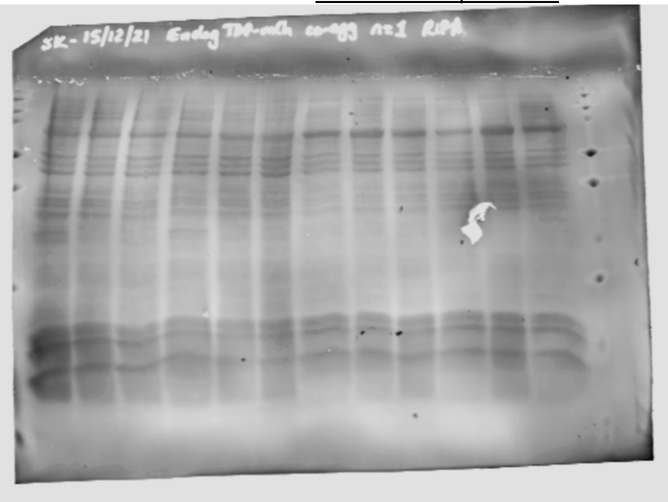

Parental | CRISPR *TARDBP-mCherry*

mGFP + - - + - -
TDP-43$^{\Delta NLS}$-GFP - + - - + -
TDP-43$^{\Delta NLS/2KQ}$-GFP - - + - - + Unrelated experiment

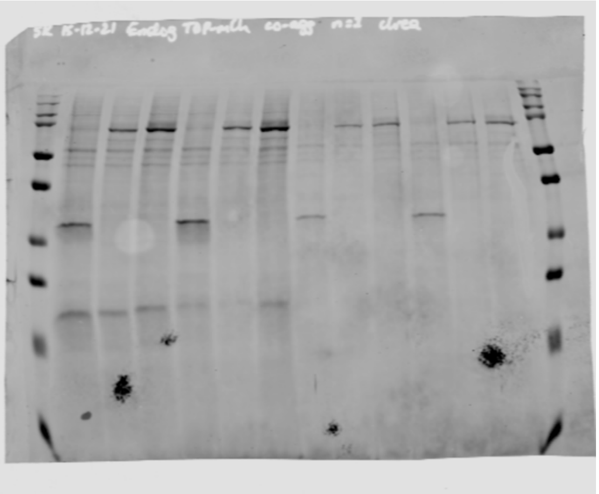

RIPA n2 **REVERT**

Parental | CRISPR *TARDBP-mCherry*

mGFP + - - + - -
TDP-43$^{\Delta NLS}$-GFP - + - - + -
TDP-43$^{\Delta NLS/2KQ}$-GFP - - + - - + Unrelated experiment

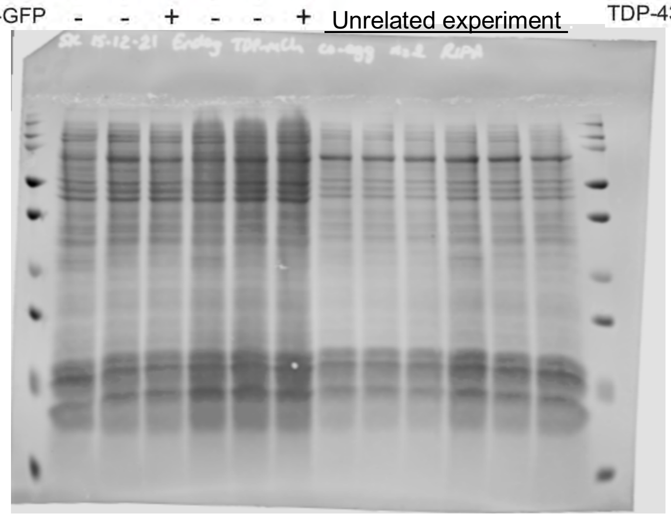

Urea n2 **REVERT**

Parental | CRISPR *TARDBP-mCherry*

mGFP + - - + - -
TDP-43$^{\Delta NLS}$-GFP - + - - + -
TDP-43$^{\Delta NLS/2KQ}$-GFP - - + - - + Unrelated experiment

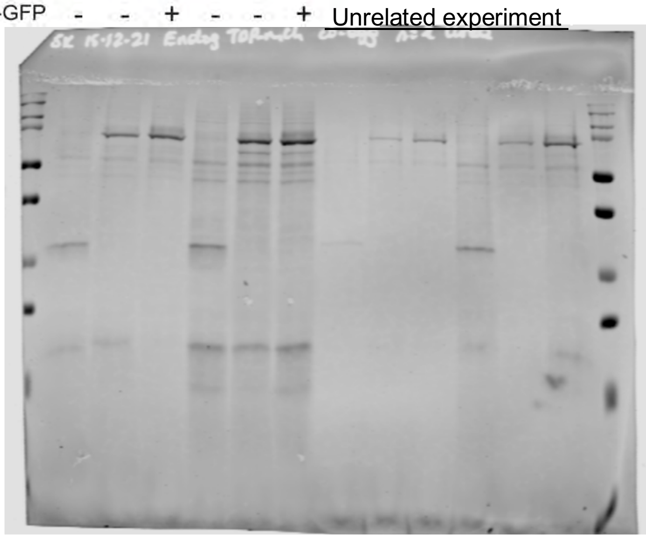

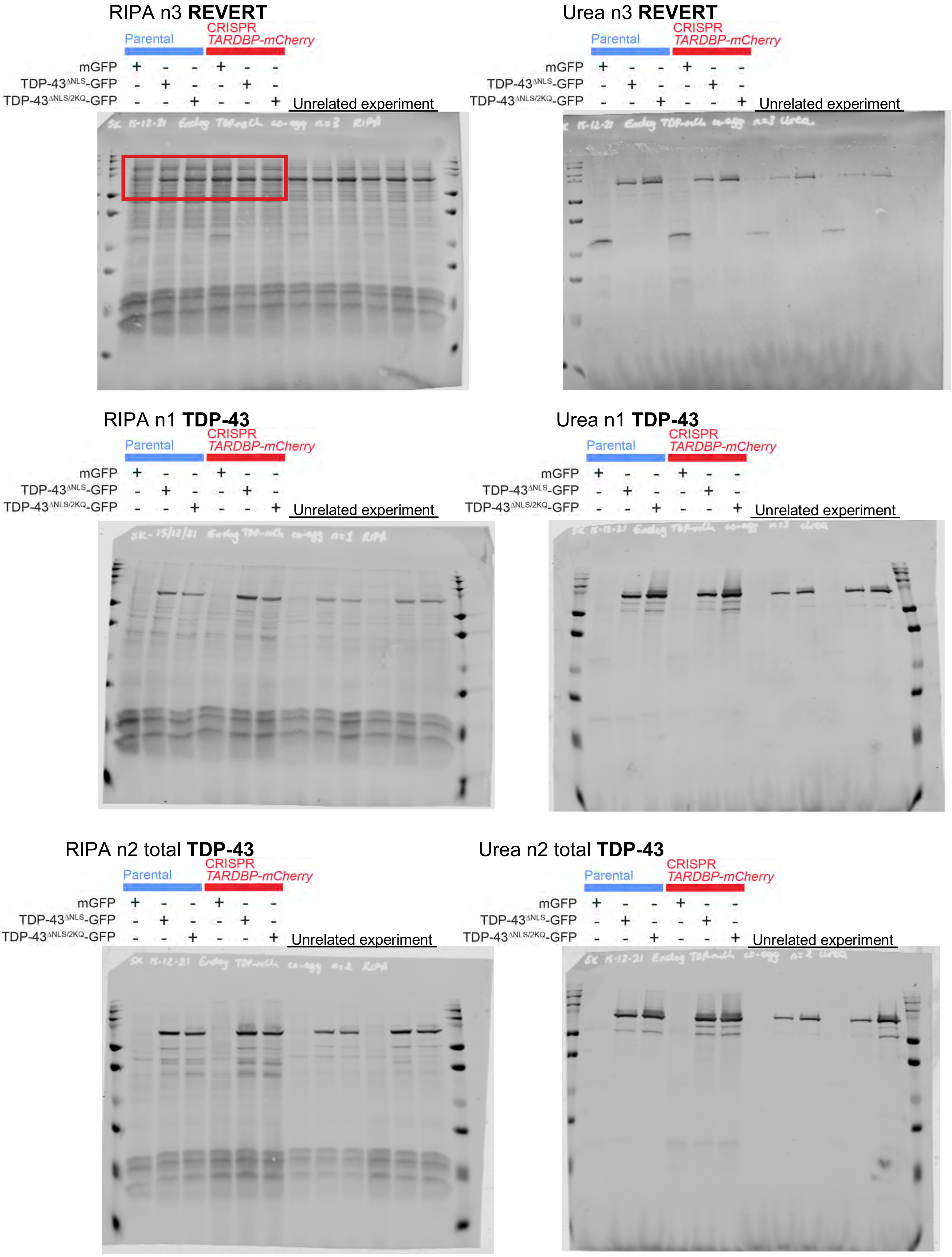
RIPA n3 REVERT
Parental
CRISPR TARDBP-mCherry
mGFP + - - + - -
TDP-43ΔNLS-GFP - + - - + -
TDP-43ΔNLS/2KQ-GFP - - + - - +
Unrelated experiment
Urea n3 REVERT
Parental
CRISPR TARDBP-mCherry
mGFP + - - + - -
TDP-43ΔNLS-GFP - + - - + -
TDP-43ΔNLS/2KQ-GFP - - + - - +
Unrelated experiment
RIPA n1 TDP-43
Parental
CRISPR TARDBP-mCherry
mGFP + - - + - -
TDP-43ΔNLS-GFP - + - - + -
TDP-43ΔNLS/2KQ-GFP - - + - - +
Unrelated experiment
Urea n1 TDP-43
Parental
CRISPR TARDBP-mCherry
mGFP + - - + - -
TDP-43ΔNLS-GFP - + - - + -
TDP-43ΔNLS/2KQ-GFP - - + - - +
Unrelated experiment
RIPA n2 total TDP-43
Parental
CRISPR TARDBP-mCherry
mGFP + - - + - -
TDP-43ΔNLS-GFP - + - - + -
TDP-43ΔNLS/2KQ-GFP - - + - - +
Unrelated experiment
Urea n2 total TDP-43
Parental
CRISPR TARDBP-mCherry
mGFP + - - + - -
TDP-43ΔNLS-GFP - + - - + -
TDP-43ΔNLS/2KQ-GFP - - + - - +
Unrelated experiment

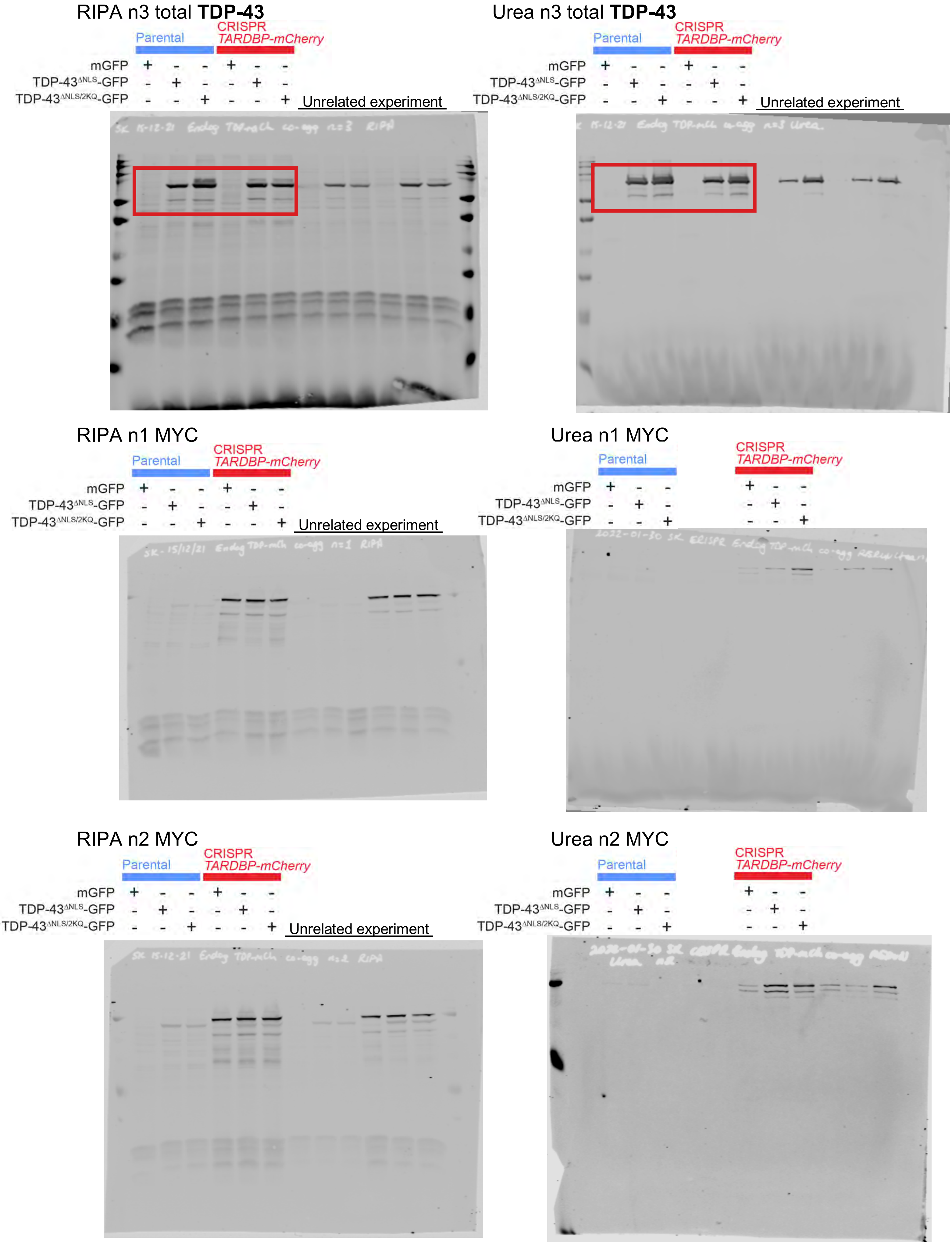

RIPA n3 total TDP-43
Parental
CRISPR TARDBP-mCherry
mGFP + - - + - -
TDP-43ΔNLS-GFP - + - - + -
TDP-43ΔNLS/2KQ-GFP - - + - - +
Unrelated experiment
Urea n3 total TDP-43
Parental
CRISPR TARDBP-mCherry
mGFP + - - + - -
TDP-43ΔNLS-GFP - + - - + -
TDP-43ΔNLS/2KQ-GFP - - + - - +
Unrelated experiment
RIPA n1 MYC
Parental
CRISPR TARDBP-mCherry
mGFP + - - + - -
TDP-43ΔNLS-GFP - + - - + -
TDP-43ΔNLS/2KQ-GFP - - + - - +
Unrelated experiment
Urea n1 MYC
Parental
CRISPR TARDBP-mCherry
mGFP + - - + - -
TDP-43ΔNLS-GFP - + - - + -
TDP-43ΔNLS/2KQ-GFP - - + - - +
RIPA n2 MYC
Parental
CRISPR TARDBP-mCherry
mGFP + - - + - -
TDP-43ΔNLS-GFP - + - - + -
TDP-43ΔNLS/2KQ-GFP - - + - - +
Unrelated experiment
Urea n2 MYC
Parental
CRISPR TARDBP-mCherry
mGFP + - - + - -
TDP-43ΔNLS-GFP - + - - + -
TDP-43ΔNLS/2KQ-GFP - - + - - +

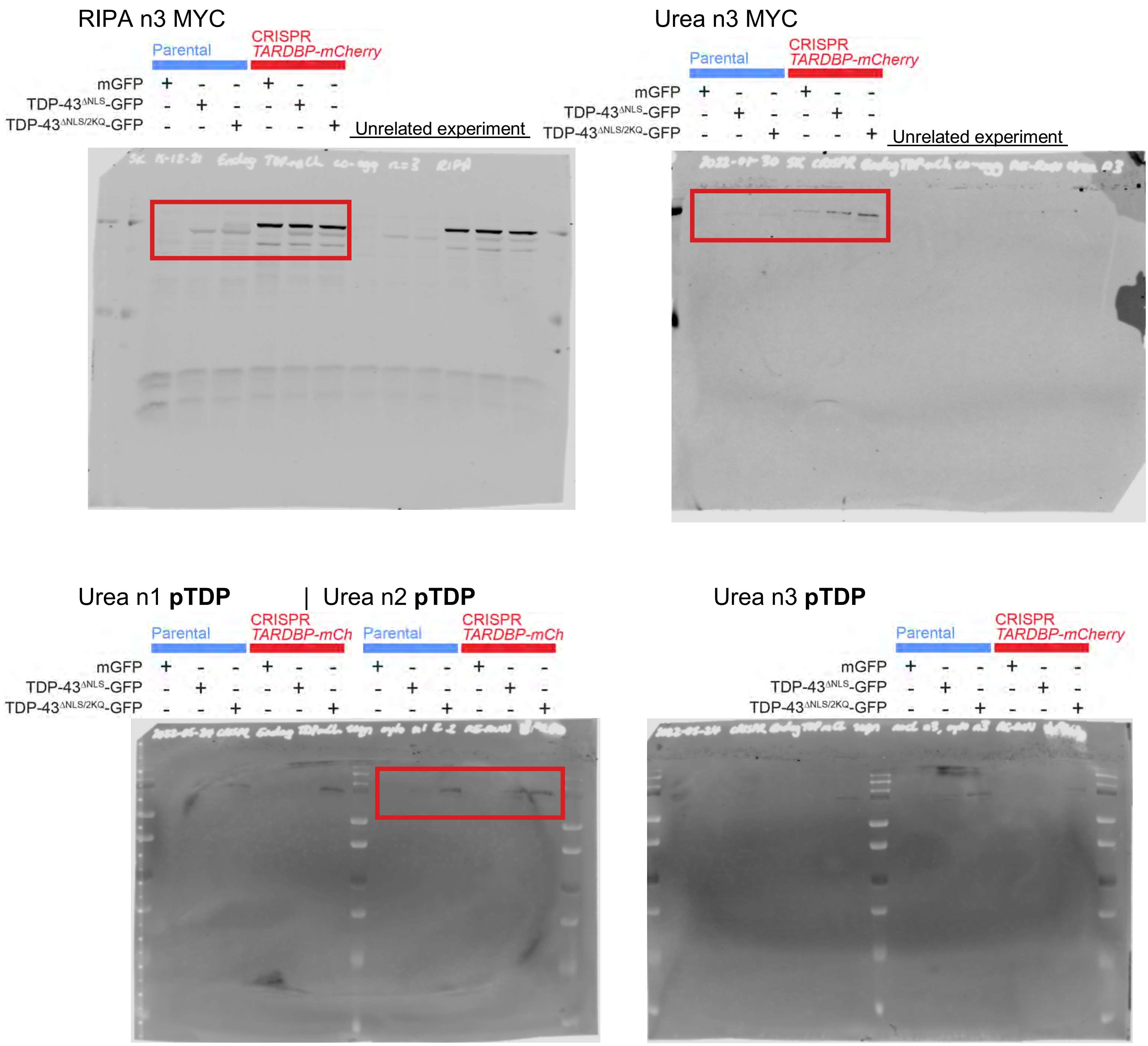
RIPA n3 MYC
Parental
CRISPR TARDBP-mCherry
mGFP + - - + - -
TDP-43ΔNLS-GFP - + - - + -
TDP-43ΔNLS/2KQ-GFP - - + - - +
Unrelated experiment
Urea n3 MYC
Parental
CRISPR TARDBP-mCherry
mGFP + - - + - -
TDP-43ΔNLS-GFP - + - - + -
TDP-43ΔNLS/2KQ-GFP - - + - - +
Unrelated experiment
Urea n1 pTDP | Urea n2 pTDP
Parental
CRISPR TARDBP-mCh
Parental
CRISPR TARDBP-mCh
mGFP + - - + - - + - - + - -
TDP-43ΔNLS-GFP - + - - + - - + - - + -
TDP-43ΔNLS/2KQ-GFP - - + - - + - - + - - +
Urea n3 pTDP
Parental
CRISPR TARDBP-mCherry
mGFP + - - + - -
TDP-43ΔNLS-GFP - + - - + -
TDP-43ΔNLS/2KQ-GFP - - + - - +

Supplementary Fig. S8: Endogenous TDP-43 is sequestered by cytoplasmic acetylated TDP-43

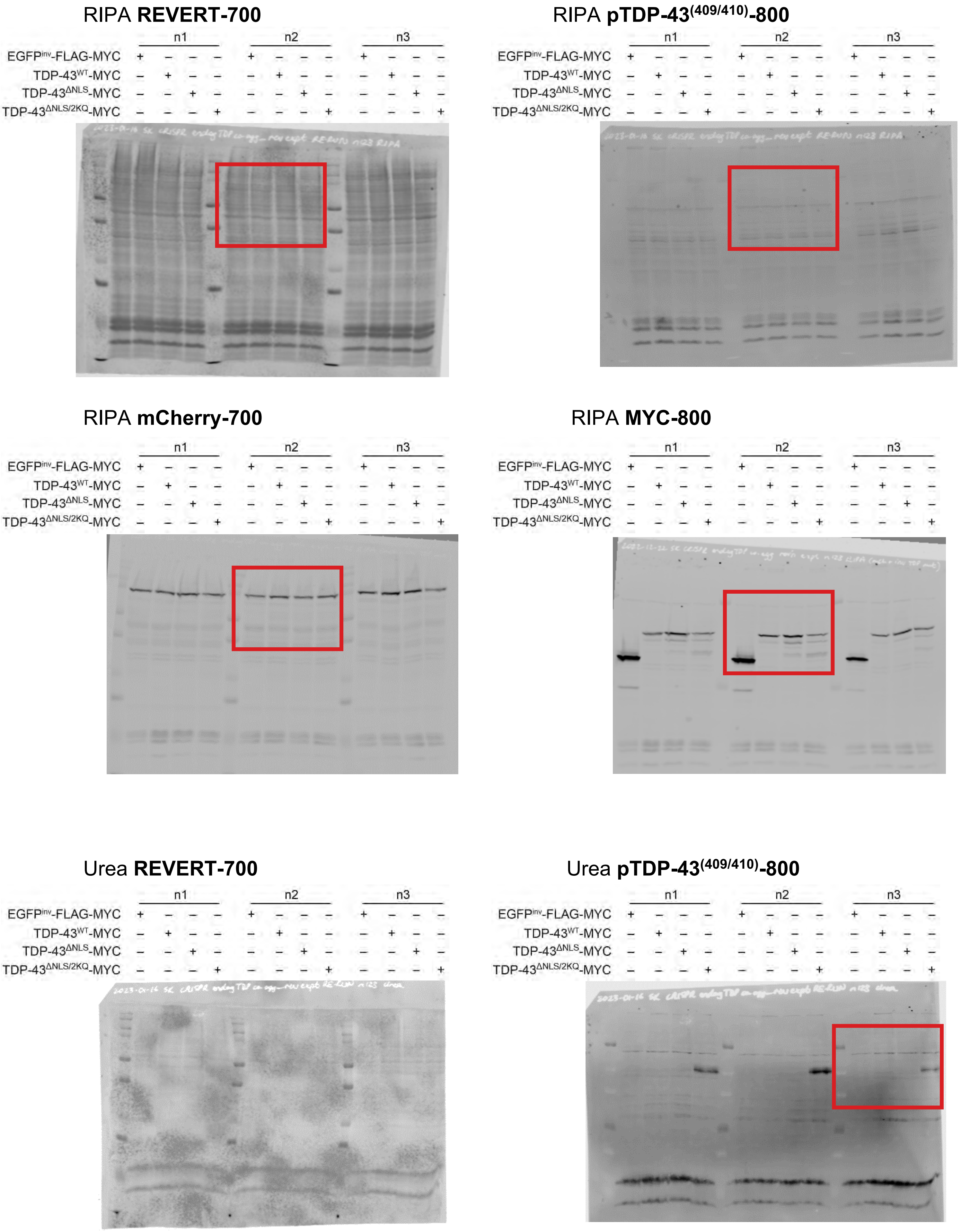

Urea mCherry-700

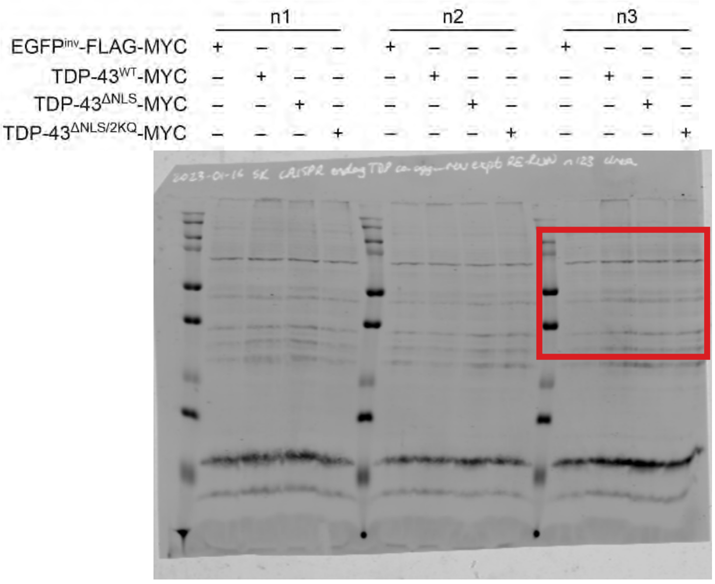

Urea MYC-800

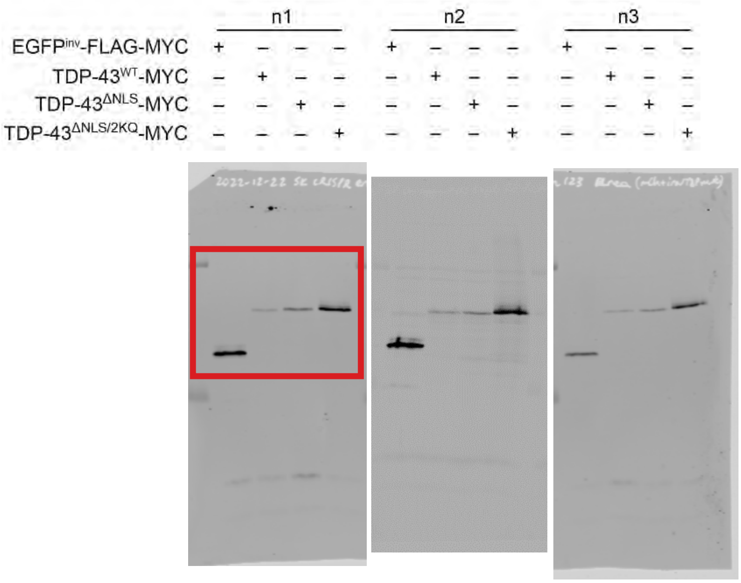

Supplement: Supplementary file 8 — Supplementary file8 (PDF 9613 KB) [file 18_2023_4739_MOESM8_ESM.pdf]
